# A Knowledge-Based Discovery Approach Couples Artificial Neural Networks With Weight Engineering to Uncover Immune-Related Processes Underpinning Clinical Traits of Breast Cancer

**DOI:** 10.3389/fimmu.2022.920669

**Published:** 2022-07-14

**Authors:** Cheng Zhang, Cristina Correia, Taylor M. Weiskittel, Shyang Hong Tan, Kevin Meng-Lin, Grace T. Yu, Jingwen Yao, Kok Siong Yeo, Shizhen Zhu, Choong Yong Ung, Hu Li

**Affiliations:** ^1^Department of Molecular Pharmacology and Experimental Therapeutics, Mayo Clinic College of Medicine and Science, Rochester, MN, United States; ^2^Department of Biochemistry and Molecular Biology, Mayo Clinic College of Medicine and Science, Rochester, MN, United States

**Keywords:** artificial intelligence, neural networks, weight engineering, gene–gene associations, clinical traits

## Abstract

Immune-related processes are important in underpinning the properties of clinical traits such as prognosis and drug response in cancer. The possibility to extract knowledge learned by artificial neural networks (ANNs) from omics data to explain cancer clinical traits is a very attractive subject for novel discovery. Recent studies using a version of ANNs called autoencoders revealed their capability to store biologically meaningful information indicating that autoencoders can be utilized as knowledge discovery platforms aside from their initial assigned use for dimensionality reduction. Here, we devise an innovative weight engineering approach and ANN platform called artificial neural network encoder (ANNE) using an autoencoder and apply it to a breast cancer dataset to extract knowledge learned by the autoencoder model that explains clinical traits. Intriguingly, the extracted biological knowledge in the form of gene–gene associations from ANNE shows immune-related components such as chemokines, carbonic anhydrase, and iron metabolism that modulate immune-related processes and the tumor microenvironment play important roles in underpinning breast cancer clinical traits. Our work shows that biological “knowledge” learned by an ANN model is indeed encoded as weights throughout its neuronal connections, and it is possible to extract learned knowledge *via* a novel weight engineering approach to uncover important biological insights.

## 1 Introduction

Immune responses are known to play important roles in broad disease etiology (1–3) and are often linked to prognosis in cancer (4). However, immunotherapy remains ineffective for most cancer patients (4). Thus, our ability to booster the detection of key genes (i.e., features) and gain knowledge on their relationships (i.e., gene–gene interactions) and roles in driving immune-related processes and clinical traits represents an applicable approach for the deployment of better immunotherapies. Genes act collectively through functional associations, and differentially expressed or mutated genes are not always the key players that underpin clinical traits. Interactions between genes also are nuanced by their cellular network ecosystem. An unbiased large-scale evaluation of genes and their positional functional associations in disease phenotypes can identify unknown gene associations that play critical roles in conferring clinical traits in cancer. Artificial intelligence (AI) approaches coupled with weight engineering methods can foster such discoveries. Here, our central tenet is that gene–gene associations that not only predict but also explain the properties of the clinical outcomes are key players in disease. We term such gene–gene associations as biological “knowledge” that is embedded within omics data and can be accessed through trained AI models.

How the human brain learns and stores knowledge has fascinated scientists for decades. Years of studies in neuroscience have revealed that connections between neurons are plastic and changes such as “pruning” occur during the learning process (5–7) to ensure efficient brain function and allow for memories to be reorganized at the systems level (8). In addition, numerous studies suggest that learned information is sparsely represented and distributed in the neocortex (9). This sparsely coded information is encapsulated as weight representations that can be found in auditory (10), visual (11), and somatosensory areas (12).

The creation of artificial neural networks (ANNs) was inspired by the neuronal architecture to model how the brain learns. The area of ANN research was first initiated by Warren McCulloch and Walter Pitts in 1943 (13). In 1949, Donald Hebb realized that it is the strength between neurons that forms the basis of learning (14). Since then, numerous versions of ANN models have been designed such as perceptron in 1958 (15) and neocognitron in 1979 (16). These models are based on adjusting neuronal weights during the learning process. Although there was a “dark age” of ANNs from the late 1960s to the early 1980s (17), the rise of ANNs especially deep neural network (18) and the development of various versions of ANN models since the late 1980s have revolutionized many learning tasks such as image, speech, and text recognitions (19); clinical diagnosis (20); Go game (21); and protein structure prediction (22) with unprecedented performance, making ANNs become the norm and mainstream in AI in recent years.

Aside from conventional recognition tasks, ANNs have been used in various discovery tasks such as disease gene and drug discoveries (23, 24). In addition, attempts to extract knowledge from biological data using ANN models have been conducted (25). For instance, using a gene expression compendium for the bacterium *Pseudomonas aeruginosa*, Tan et al. have recently demonstrated that hidden nodes of a trained denoising autoencoder were enriched with molecular representations that explained the biology of *P. aeruginosa (* 26*).* They also found that one of the hidden nodes was enriched by genes that were co-regulated in an operon and another hidden node was enriched with genes that signified different bacterium strains. Another work by Tan et al. showed that denoising autoencoders can help to construct features that contain clinical and molecular information with respect to normal and tumor including estrogen receptor status and molecular subtypes of tumors (27). All these studies indicate that autoencoders act as information encoders aside from their initial use as dimensionality reduction (28). Autoencoder-learned knowledge is stored using weight representations that compromise “weighted connectome patterns”. In other words, aside from their conventional use in recognition and discovery tasks, ANNs, in particular autoencoders, can be used as knowledge discovery platforms.

Our current study attempts to explore the feasibility of extracting knowledge from an ANN model using a novel weight engineering approach. Here, “knowledge” refers to the relationships between features (e.g., gene–gene associations), in particular associations involving immune-related genes that can explain properties of a disease system (e.g., a clinical trait in cancer). This study seeks to decode weight representations of learned information in an ANN model and reconstruct underlying relationships between input features (i.e., knowledge) that explain the behavior of a system. We use an autoencoder as a model of choice and design a weight engineering platform called artificial neural network encoder (ANNE) to explore how biological knowledge can be reconstructed from an ANN model. As a proof-of-concept study, we employ ANNE on a breast cancer gene expression dataset with known clinical outcomes to extract the roles of immune-related processes in underpinning clinical traits from knowledge learned by autoencoder models.

## 2 Materials and Methods

### 2.1 Data Preparation and Normalization

Gene expression data from a breast cancer cohort conducted from June 2000 to March 2010 at the MD Anderson Cancer Center and treated with neoadjuvant taxane–anthracycline chemotherapy (29) were downloaded from the Gene Expression Omnibus (Accession ID: GSE25066). Gene expressions were read using an Affymetrix Human Genome U133A Array platform with a total of 11,840 genes measured across 508 breast cancer patients. The expression profile of each sample was normalized individually using the single-channel array normalization (SCAN) method developed by Piccolo et al. (30) which is implemented in the SCAN function from R package SCAN.UPC (31). This allows our models to easily incorporate new samples. SCAN-processed gene expression data were further normalized into Gaussian distribution *N*(0, 1) for each gene.

#### 2.1.1 Chemosensitivity Models

Pathological complete response (pCR) indicating no invasive or metastatic breast cancer was used to define patients who were sensitive to neoadjuvant chemotherapy. Residual disease (RD) defined as a surrogate for residue cancer cells in a patient during treatment was used to classify patients who were resistant to chemotherapeutics. Expression data from pCR and RD were used to build models that were sensitive and resistant to chemotherapy, respectively.

#### 2.1.2 Prognosis Models

We used information for disease relapse-free survival (DRFS) that indicates the length of time after primary treatment that a patient survives without any signs of relapse of breast cancer to define prognostic outcomes. Median DRFS (in days) was used as a cutoff to separate patients belonging to either good or poor prognosis.

### 2.2 Hyperparameter Selection

To select the combination of hyperparameters that can achieve the best performance, a grid scan was performed for six different types of activation functions, namely, linear, exponential linear (ELU), rectified linear (RELU), parameterized rectified linear (PRELU), sigmoid, and hyperbolic tangent (tan*h*), in combination with different numbers of hidden layer nodes of 30, 50, 100, 300, 500, and 1,000. The performance of each combination of hyperparameters was assessed using a 10-fold cross-validation procedure with a holdout set. Here, 10% of the samples were first randomly selected and kept as a holdout set, and the remaining samples were randomly split into 10 portions. For each hyperparameter combination, one model was trained with one portion as the validation set and the rest of the 9 portions as the training set, and this process was repeated 10 times each with a different split as validation. Training is complete when the loss function stops improving, or training has reached 10,000 epochs. Validation performance was used to select hyperparameter combinations, and performance on the holdout set was reported as model performance. We selected a linear activation function with 50 hidden layer nodes with consideration of performance, computation cost, and ease of interpretation. With selected hyperparameters, samples of each of the four subsets, pCR, RD, good prognosis, and poor prognosis, were used to build a full model for subsequent node annotation and network generation.

### 2.3 Model Training Details

Models were trained using an autoencoder algorithm, which has the structure of a feedforward neural network, while it is an unsupervised learning method that takes each of its input vectors as the training target (28). The autoencoder models were built and trained using the Python library Keras. All autoencoder models shared the same network architecture that consists of three layers: an input layer, a hidden layer, and an output layer. The numbers of nodes in the input and output layers are the same as the total number of genes (i.e., the input and output layers have the same number of nodes). Each node in the hidden layer is fully connected to all nodes in the input layer, and each node in the output layer is in turn fully connected to all nodes in the hidden layer. The goal of the training process is to minimize the difference between the reconstructed output to the input.

The weight and bias terms for each neuron in the hidden layer and output layer are initialized according to He et al. (32) so that they conform to a Gaussian distribution $N\left(0,\sqrt{2/n_{l}}\right)$ where *n_l_* is the number of nodes in layer *l*. During the feedforward phase, each sample, in the form of gene expression vectors, is fed into the input layer. The activations of each node in the hidden and output layer nodes are calculated as the weighted sum of activations of all nodes from a previous layer plus a bias term as follows:


[1] $$a_{i,l}=\sum_{j\in \left[1,\;n_{l-1}\right]}w_{i,j,l-1}*a_{j,l-1}+b_{i,l}$$

where *a_i,l_* is the activation value of node *i* in layer *l*, *n_l_*_–1_ is the total number of nodes in layer *l* − 1, *w_i,j,l_*_–1_ is the weight between node *i* in layer *l* and node *j* in layer *l* − 1, and *b_i,l_* is the bias term of node *i* in layer *l*.

To train the model, we used mean-squared error (MSE) as the loss function:


[2] $$MSE=\frac{1}{K}\sum_{k\in \left[1,\;K\right]}\sum_{i\in \left[1,\;I\right]}{\left(a_{i,o,\;k}-g_{i,\;k}\right)}^{2}$$

where *K* is the total number of samples, *I* is the total number of genes, *a_i,o,k_* is the activation value of node *i* in the output layer for sample *k*, and *g_i,k_* is the expression value of gene *i* of sample *k*.

### 2.4 Prediction of Clinical Phenotypes

To predict sample phenotypes, i.e., resistance or sensitivity to chemotherapy, or whether a sample belongs to good or poor prognosis, all samples were again split into 10 portions and stratified according to chemotherapy sensitivity or prognosis. By training models in each fold with the selected hyperparameter combinations described in previous sections, we can predict and assign to which group a new sample belongs by comparing the reconstruction MSE to each trained model. Specifically, a sample is assigned to a good prognosis group if the model for good prognosis has better reconstruction accuracy and vice versa. Misclassification rates of the test samples were calculated at selected training epochs.

To visualize the separation of the prognosis groups, information on DRFS was plotted as Kaplan–Meier overall survival curves, where the *x*-axis is the time (days) from the start of the study and the *y*-axis is the percentage of patients with DRFS. The prognosis groups were either assigned using median actual DRFS as cutoff or using predicted prognosis class. *P*-values for group separation were determined using the Cox proportional hazards regression model (33, 34). Kaplan–Meier plots were generated using the R package survival.

### 2.5 Gene Set Enrichment Analysis on Hidden Nodes

For each hidden node input, genes were ranked by their weights and the gene set enrichment analysis (GSEA) algorithm was performed on these ranked gene lists according to Subramanian et al. (35) and Mootha et al. (36) using gene sets from MSigDB version 3.0 (37).

### 2.3 Weight Engineering Function to Extract Encoded Gene–Gene Associations Within an Autoencoder Model

We designed a novel weight engineering approach with an association scoring scheme to decode meaningful information (in this case, gene–gene associations) encoded in a learned autoencoder model. We used the sum of cumulative weights from every input node to every output node as a score to access the association strength of every gene as input to every gene as output. Our devised association score *S_i,o_* of gene *I* at input layer *I* to gene *o* at output layer *O* is given in the equation below:


[3] $$S_{i,o}=\sum_{o\in O}\sum_{h\in H}w_{o,\;h}w_{h,\;i}$$

where *O* and *H* denote the output layer and the hidden layer, respectively. *w_h,i_* denotes the trained weight from node *i* in the input layer to node *h* in the hidden layer, and similarly, *w_o,h_* denotes the trained weight from node *o* in the output layer to node *h* in the hidden layer. The association scores of all gene pairs connect the genes into a network where the vertices are genes, and each edge has the direction of the association score of the gene pair which the edge connects. The magnitude of the association score (or edge weight) for a gene pair is represented for visualization purposes by the thickness of its edge. In the current study, subsets of the top 200 gene pairs with the largest absolute edge weight are considered. The decoded networks for all the proposed models can be explored at www.anne.hulilab.org.

## 3 Source Code

The source code for ANNE, along with sample datasets and installation instructions, can be downloaded at www.anne.hulilab.org and on GitHub at https://github.com/HuLiLab/ANNE.

## 4 Results

### 4.1 Basic Concepts on Weight Engineering to Extract ANN Weight-Encoded Biological Knowledge

**Figure 1:**
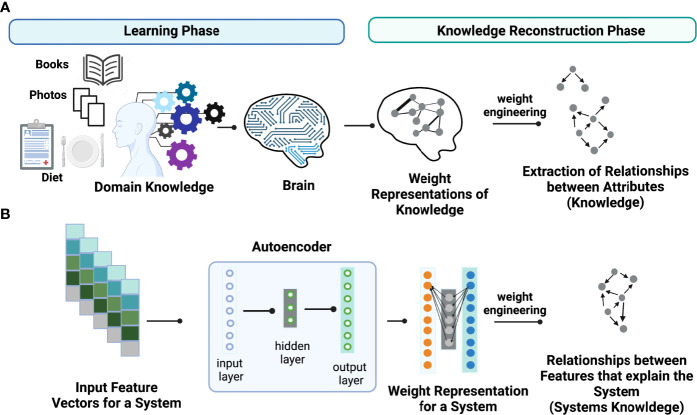
Conceptual illustration of learned knowledge. Weight engineering is utilized to extract meaningful learned knowledge by a learner such as **(A)** the human brain and **(B)** an artificial neural network (ANN) model including an autoencoder. During the learning process, the learner (brain or ANN) translates the learned associations between attributes or features that explain the property of a system, that we call “knowledge”, into weight representations that are stored as synaptic strengths in the brain or interneuronal weights in an ANN model. Weight engineering, a mathematical approach that manipulates learned weights stored from a learner, reconstructs the learned knowledge by decoding associations between features (i.e., gene-gene interactions) that can explain the properties of a desired system. Figure generated with BioRender.com.

The key idea of weight engineering to extract encoded knowledge from ANN models is based on findings in neuroscience showing that the brain learns *via* adjusting synaptic strengths between neurons. In other words, the learned knowledge is stored in the form of “weight representations” between synapses across distinct regions in the brain (**Figure 1A**). Under this context, “knowledge” is defined as relationships between attributes (i.e., events or objects of learning) that explain the property of systems learned. In principle, it is possible to reconstruct learned knowledge to a certain extent in an individual if we can map the full connectome of the brain and know their synaptic strengths. This task can be accomplished *via* “weight engineering”, a mathematical approach to manipulate these weights to extract stored knowledge. Although extracting stored knowledge in the brain is still infeasible, it might be possible on simpler ANN models such as autoencoders (**Figure 1B**). Just as in the brain, ANNs store learned knowledge in the form of weight representations throughout the models. Hence, it is possible to employ a weight engineering approach to decode stored knowledge in ANNs.

### 4.2 The Design of the Artificial Neural Network Encoder Platform

We designed a computational platform called ANNE to decode learned knowledge using a novel weight engineering method. Among ANNs, we chose an autoencoder as a model of choice as it has previously been demonstrated to possess the power to store known knowledge (25–27). In addition, the architectures of autoencoders are symmetric in “bowtie-shape” form with similar dimensionality of input and output layers. Such symmetric architectures enable us to design a weight engineering function (see *Materials and Methods*) to access the association strength of each input to output gene. Next, we used gene expression data from a breast cancer cohort conducted from June 2000 to March 2010 at the MD Anderson Cancer Center for patients subjected to neoadjuvant (preoperative) taxane–anthracycline chemotherapy with known clinical phenotypes (29) as study cases.

**Figure 2:**
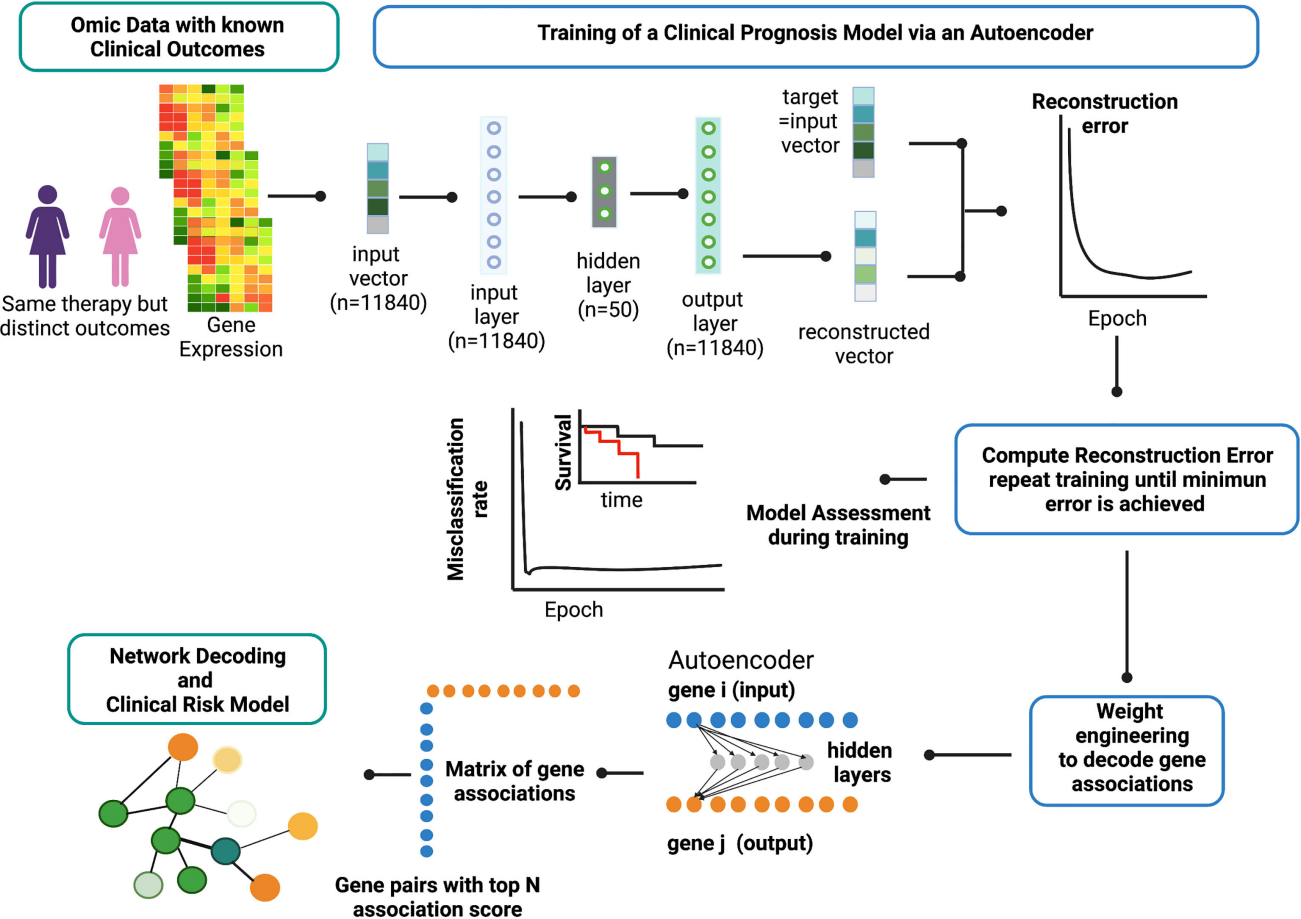
Overview of Artificial Neural Network Encoder (ANNE). The ANNE algorithm uses gene expression profiles from breast cancer patients with known prognostic outcomes to simultaneously decode gene-gene associations, networks and derive clinical risk models. Patient samples are assigned to either good or poor prognosis according to disease relapse-free survival (DRFS). Next, an autoencoder algorithm is used to train the models, with an input vector that represents all genes (features) present in transcriptomics data and each gene corresponds to a node in the input layer. The dimensionality of the output layer (i.e., number of nodes) is the same as the input layer. The autoencoder has an architecture of one input layer, one hidden layer and one output layer. During the training phase the autoencoder reconstruct values from input layer into an output layer and the output vector is compared with input vector to compute the reconstruction error. The training process is repeated by updating weights connecting nodes (or neurons) from input layer to hidden layer and from hidden layer to output layer via a backpropagation algorithm. The training process is repeated by feeding input through the neural network to the output layer to calculate the training loss, and updating the weights connecting nodes from the output layer to hidden layer and to the input layer via a backpropagation algorithm. Training process is deemed complete when no further improvements on the reconstruction error is achieved, or training has reached certain number epochs. Next, the learned weights connecting all nodes from input to output layers in a trained autoencoder model are used to decode meaningful gene-gene associations using an association scoring scheme. Computed association scores for all gene pairs are aggregated to an association score matrix and the top gene pairs (n=200) with highest absolute scores are selected to build ANNE-decoded networks. Genes that occur multiple times will serve as “anchors” to agglomerate gene pairs into a network. Figure generated with BioRender.com.

**Figure 3:**
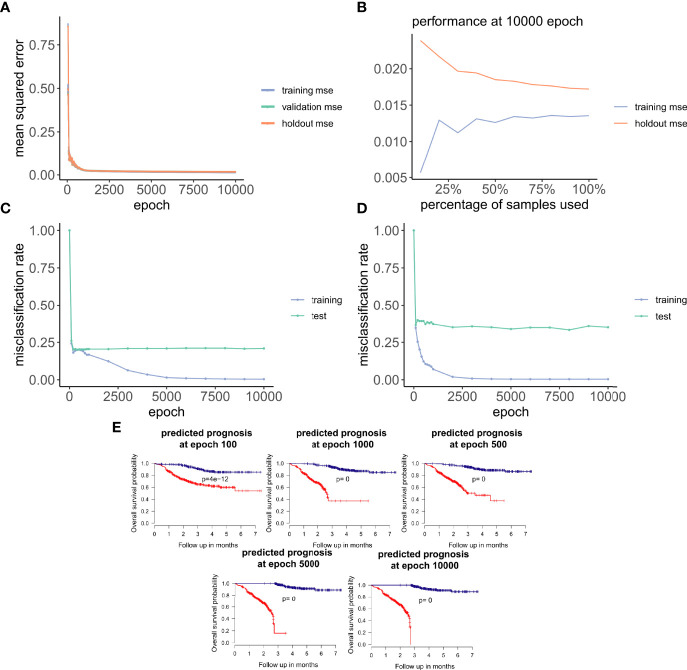
ANNE model training and evaluation for models trained with a linear activation function. **(A)** Training curve showing the loss function of mean-squared-error (MSE) with epochs during training, validation (10-fold cross-validation), and holdout (excluded 10% of the data) sets with a linear activation function. **(B)** Change of performance for training and holdout sets with 10000 training epochs and by varying batch size. During model validation MSE improves as more samples are used for training, indicating that the model is learning from its input gaining information which can be extended to unknown samples. **(C, D)** Performance class prediction (misclassification rates) for chemo-sensitive and resistant (pCR and RD) and prognosis (good and poor) models improves with epochs trained. **(E)** Kaplan-Meier overall survival plots for poor and good breast cancer prognosis groups at different epochs trained. (100, 500, 1000, 5000,

**Figure 2** provides an overview of the ANNE platform using a breast cancer prognosis model as an illustrative example. The basic architecture of the autoencoder contains one visible input layer, one hidden layer, and an output layer. As illustrated in **Figure 2**, when gene expression profiles for patients with known prognostic outcomes are used, the training goal for an autoencoder is to minimize the reconstruction error between the input vector (i.e., original vector data of gene expression values from patients) and output (i.e., reconstructed input) for example for good and poor prognosis models. The training process is repeated until no further improvement for data reconstruction is achieved as reflected and measured by MSE or reaching 10,000 training epochs (**Figure 3A**). Weight engineering functions are expressed by an association scoring scheme where cumulative products of weights of all possible paths connecting nodes of a gene pair *i*–*j* from input to output layers *via* hidden nodes are then computed. The resulting cumulative association score matrix contains the information on the extent (i.e., strength) and directionality (i.e., positive or negative) of gene–gene associations. Gene pairs that exhibit strong informational associations can be extracted, and genes recurrent in multiple gene pairs will serve as “anchors” to allow us to agglomerate gene pairs into a network. The resulting network represents the reconstructed “knowledge” where gene–gene associations can help explain the prognostic behavior in breast cancer (the full reconstructed networks are given at www.anne.hulilab.org).

### 4.3 Model Construction and Training

We build two sets of models in this study based on chemosensitivity (chemosensitive and chemoresistant) and prognosis (good and poor). In total, we constructed four autoencoder-based models (chemosensitive, chemoresistant, good prognosis, and poor prognosis), and the training procedure was conducted as outlined in **Figure 2** for each model.

**Figure 4:**
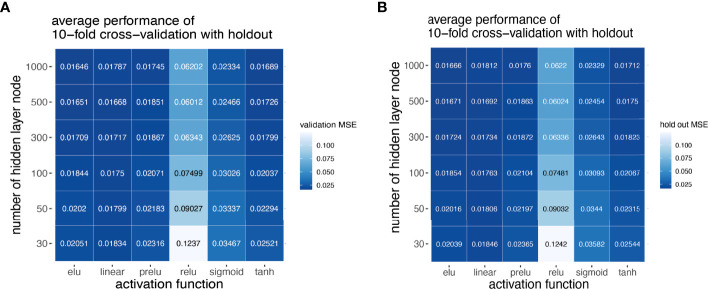
Model selection using grid scan for combination of hyper-parameters. Different activation functions and number of hidden nodes using 10-fold cross-validation with holdout set. Hyper-parameter combinations were evaluated with validation MSE **(A)**, and model performance reported in holdout MSE **(B)**.

**Figure 5:**
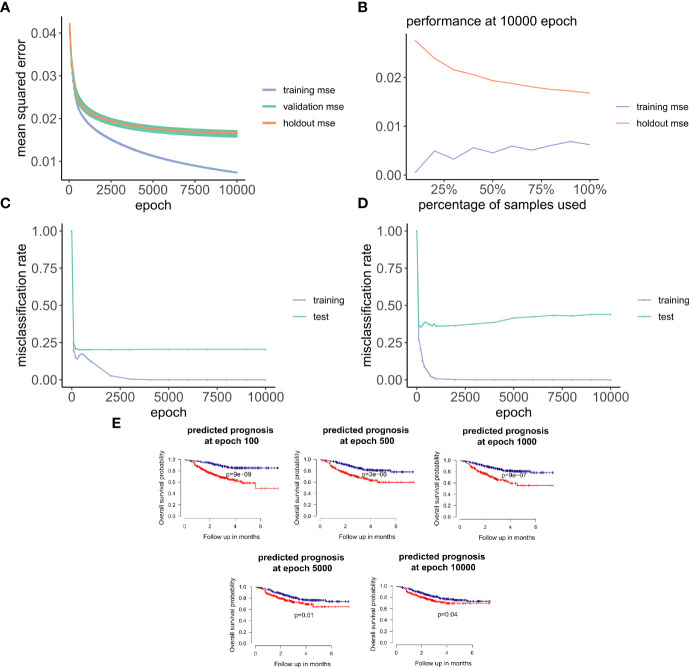
Training and evaluation for models trained with nonlinear ELU activation function. **(A)** Training curve showing that the mean-squared-error (MSE) of training, and holdout sets improves with training. **(B)** Change of training and holdout set performance with different training sample sizes, at 10000 epochs. **(C, D)** Performance of class prediction in misclassification rates for chemo-sensitive and resistant (pCR and RD) and prognosis (good and poor) improves within training. **(E)** Kaplan-Meier overall survival plots for poor and good breast cancer prognosis groups with varying number of trained epochs:100, 500, 1000, 5000, and 10000.

Next, we explored how hyperparameters, i.e., the number of hidden layer nodes (30, 50, 100, 300, 500, and 1,000) and six types of activation functions (linear, ELU, PRELU, RELU, sigmoid, and tan*h*), affected the performance of these autoencoder models. We found that the activation function ELU with 1,000 hidden layer nodes showed the best validation performance (i.e., the least mean-squared errors) with 10-fold cross-validations (**Figure 4A**) and holdout dataset (**Figure 4B**). Furthermore, the performance on the holdout dataset (27) also improves while the training performance improves, showing that no overfitting occurs during model training (**Figure 5A**). Interestingly, we observed that models trained *via* a linear activation (Equation 1) showed comparable performance with the ELU-derived models (**Figure 4**). In addition, the models trained with linear and ELU activation functions have robust and comparable performance using MSE as loss function during training, validation, and holdout test (**Figures 3A**, **5A**). Also, the sample sizes that increased the training set led to better holdout performance (**Figures 3B**, **5B**) as well as performance of class prediction for pCR–RD (**Figures 3C**, **5C**) and prognosis (**Figures 3D**, **5D**). We next tested the models trained with ELU and a linear activation function for their capability to separate prognosis groups. Here, we found that the linear models separate the prognosis groups well (**Figure 3E**) but not the ELU models (**Figure 5E**). We therefore selected models trained with a linear activation function for the subsequent analyses. To reduce the computation cost and enhance the interpretability of the trained models such that pathway enrichment analyses can be performed and analyzed on each of the hidden nodes, we choose models with 50 hidden nodes that possess comparable performance instead of the best performing models with 1,000 hidden nodes.

Based on the above criteria, all four sets of models (chemosensitive, chemoresistant, good prognosis, and poor prognosis) shared a similar neural network architecture, with 11,840 nodes at the input layer (corresponding to 11,840 genes in the input vector), 50 nodes at the hidden layer, and 11,840 nodes at the output layer (corresponding to 11,840 genes of the reconstructed output vector), and were trained using the linear activation function.

### 4.4 Hidden Nodes Encoded Insightful Biological Information

Earlier studies have demonstrated that nodes within the hidden layer of an autoencoder are capable to learn appropriate compressed representations that describe a dataset (25–27). As such, we reason that hidden nodes in our trained models are also capable to capture meaningful molecular representations (genes and their associations) that signify different molecular aspects of breast cancer biology.

**Figure 6:**
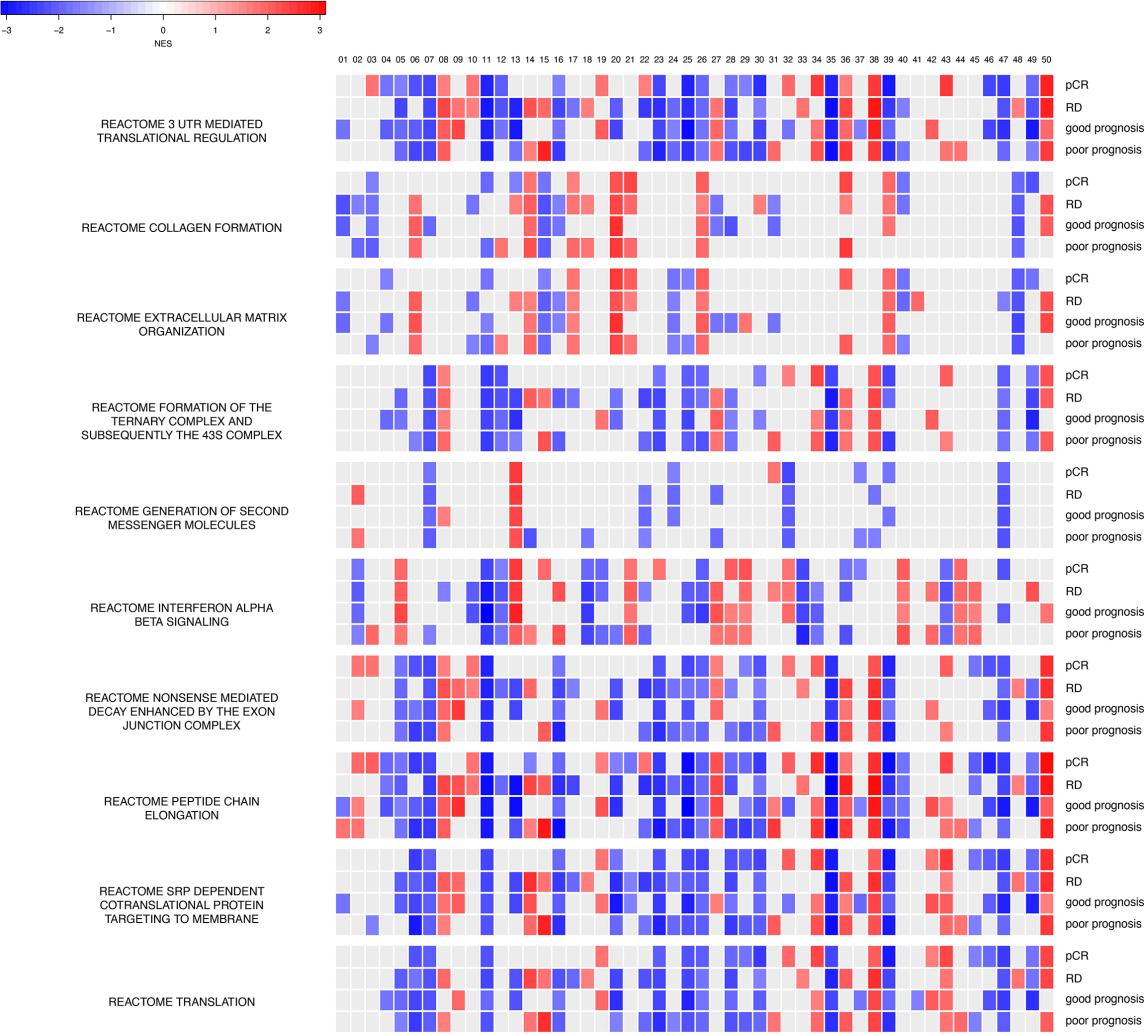
Hidden nodes store biologically meaningful information. GSEA enrichment analyses for protein-protein interactions using the REACTOME database for top hidden nodes in ANNE trained model. Enriched interactions with normalized enrichment scores with absolute values greater than 1.5 and false discovery rate (FDR) < 0.05 are indicated. pCR, chemosensitive model; RD, chemoresistant model. The order of the 50 hidden nodes is indicated at top of the figure.

To investigate what information has been learned and decoded in our trained models, we performed GSEA for each hidden node (a total of 50 hidden nodes) for gene sets in the MSigDB database (**Figure 6**). Since all four models (chemosensitive, chemoresistant, good prognosis, poor prognosis) share the same neural network architecture and training parameters, we can directly overlay the enrichment results, node-by-node in the hidden layer. Enrichment results provided in **Figure 6** not only revealed the intimate functional similarity between chemosensitivity and prognosis but also highlighted the differential functional connections between the two clinical setups. For instance, REACTOME Extracellular Matrix Organization, REACTOME Interferon Alpha Beta Signaling, and REACTOME Nonsense-mediated Decay Enhanced by the Exon Junction Complex (**Figure 6**) encapsulate the protein interactions that exhibit the most differential functional profiles between the good and poor prognosis models in their hidden nodes. As described below, the extracellular matrix (ECM) and collagen are reported to play a role in determining chemosensitivity and prognosis in breast cancers, indicating that our learned autoencoder models indeed encode meaningful information enriched with relevant biological functions that explain the clinical-relevant properties in breast cancer.

Next, we assessed the impact of different sets of initialization parameters on trained weights and converged modes. We used 10 different random seeds to train the models (linear activation function with 50 hidden nodes) and investigated whether meaningful information still can be encoded for the models trained. Thus, we built 50 GSEA models (with respect to each hidden node) for pCR, RD, good prognosis, and poor prognosis states. We found that the models trained with different initialization parameters gave rise to different GSEA results compared to our “reference” models (**Supplementary Data 1**). Nonetheless, all these enriched pathways are indeed pertinent to cancer etiology (e.g., telomere maintenance and respiratory electron transport) with processes related to protein synthesis (e.g., 3′-UTR-mediated translation regulation and peptide chain elongation) being the most commonly shared enriched processes. Such observations further support that our models indeed encode biologically meaningful information, but each model explores and encodes the weight space into knowledge of a system in a slightly different “perspective”.

### 4.5 Knowledge Extraction From ANNE-Encoded Networks

In our work, we defined learned “knowledge” as ANNE-decoded gene–gene associations that explain the clinical properties of breast cancer. This is because these gene–gene associations not only indicate which genes play more important roles in model prediction but also provide mechanistic insights that help characterize the property of a system. We reasoned that weights of all possible paths connecting gene *i* at the input layer to gene *j* at the output layer (where *i* ≠ *j*) *via* nodes at the hidden layer of an ANN model encode information that indicates the importance of the functional association between genes *i* and *j*. We developed an association score scheme by computing the collective sum of products of weights connecting all possible paths from gene *i* at the input layer to gene *j* at the output layer to encode their functional associations (Equation 3). The magnitude of association scores provides an estimate of predictability of expression outcome for gene *j* given the expression of gene *i* (denoted as *i* → *j*). In some scenarios, both genes *i* and *j* show mutual predictability of expression outcome to one another (denoted as *i* ↔ *j*). Furthermore, the cumulative association score for gene pair *i*–*j* can be either positive or negative, indicative of a positive or negative predictive effect of gene *i* to gene *j*, respectively. Gene pairs of the top 200 absolute association scores were used to decode chemosensitive (pCR), chemoresistant (RD), good prognosis, and poor prognosis networks, as depicted in **Supplementary Figures S1**-**S4**.

**Figure 7:**
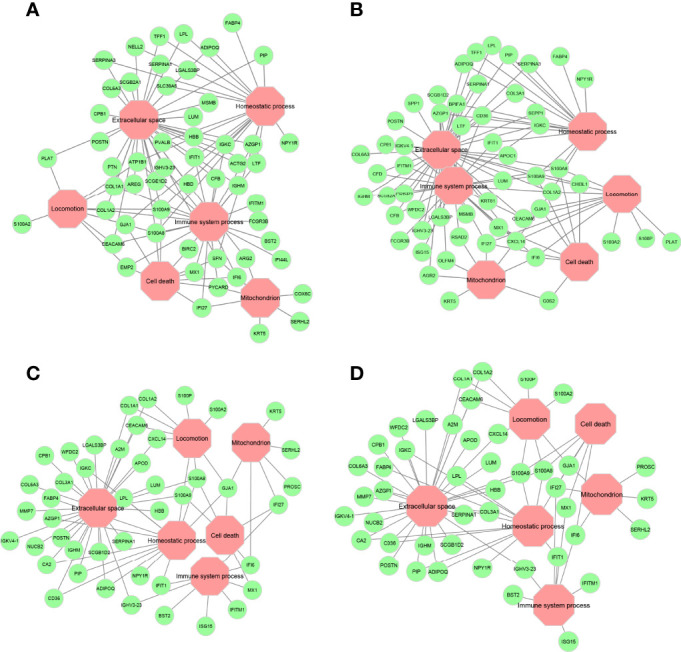
Major gene ontology (GO) terms for ANNE decoded networks. **(A)** Good prognosis. **(B)** Poor prognosis. **(C)** Chemosensitive (pCR). **(D)** Chemoresistant (RD). Octagonal nodes (pink) represent GO processes. Circular nodes (green) are genes detected in encoded networks derived from top 200 gene pairs with the largest absolute association score.

Overlay of the functional annotations from Gene Ontology (GO) indicates that extracellular space, immune system process, homeostatic process, cell death, mitochondrion, and locomotion are the major biological processes where most genes are found to be residing in these encoded networks (**Supplementary Figures S1**-**S4**). These processes, especially extracellular space and immune system process, are pertinent to determine chemosensitivity and prognosis in breast cancers, as discussed below. Key genes that connect these major biological processes are given in **Figure 7**. Calcium-binding proteins S100A8 and S100A9 are hub genes that connect to all major biological processes except the mitochondrion in all four encoded networks, suggesting that these genes might be important in mediating chemosensitivity and prognosis, since dysregulated expression of the members of the S100 family is a common feature of human cancers (38).

To better understand the biological and clinical implications of ANNE-encoded networks, modules were assigned based on network connectivity to hub genes. **Supplementary Figures S5**, **S6** provide common key modules for chemosensitivity and prognosis networks, respectively. The results highlighted in these figures indicate integrated yet marked functional associations between genes that play similar biological roles. Furthermore, distinct functional associations were also detected. For instance, both KRT14 and S100A8 modules are found in chemosensitive and chemoresistant networks (**Supplementary Figure S5**) but show distinct gene–gene associations. These differential gene–gene associations provide important clues that warrant further investigations to understand molecular mechanisms leading to variations in clinical response.

Next, we analyze the expression of hub genes and their associated genes to investigate to what extent these genes show differential expression profiles between different clinical phenotypes. **Supplementary Figure S7** shows that many hub genes and their associated genes in all four encoded networks are not differentially expressed. This indicates that it is the product of weights connecting gene *i* and *j* rather than a differential expression of these genes that determines the importance of their associations. Association score implemented in ANNE is therefore capable to decode knowledge of meaningful gene–gene associations encoded by learned autoencoder models that capture the property of a given clinical phenotype (e.g., prognosis).

### 4.6 Decoded Gene–Gene Associations From ANNE-Encoded Networks Revealed the Importance of Immune-Related Processes in Cancer Clinical Traits

To illustrate the trained autoencoder models encode meaningful information from breast cancer expression data with known clinical outcomes, we next investigated whether the decoded gene–gene associations especially those that correspond to immune-related processes from ANNE-encoded networks can explain the biology of chemosensitivity and prognosis for breast cancers. Indeed, we found numerous reported studies supporting the clinical roles of these genes in chemosensitivity and prognosis, and more importantly, all this supporting evidence pointed to different molecular aspects found in the encoded network modules (**Supplementary Figures S5**, **S6**) as outlined below.

#### 4.6.1 Chemokine Responses

The immune surveillance theory asserts that tumors rise due to failure of anticancer immune responses (39). Both innate and adaptive immune responses are known to have a crucial contribution to outcomes of conventional chemotherapy-based anticancer treatments (40). Meta-analyses of transcriptomic data revealed that chemokine (C-X-C module) ligand 13 (CXCL13) within the CXCL9 module of the chemosensitive (pCR) model (**Supplementary Figure S5**) is one of the most robust predictors of favorable disease outcome for breast cancer patients treated with neoadjuvant chemotherapy (41). In the context of neoadjuvant chemotherapy, the expression levels of multiple immune-related genes including *CXCL13* constitute positive prognostic factors for pCR (42), and it has been demonstrated to be associated with improved disease-free and overall survival after tumor resection (43). Also, *CXCL9*, the hub gene in this module (**Supplementary Figure S5**) within the chemosensitive network, was found to be significantly differentially expressed in the recurrence versus the non-recurrence group, and its elevated expression level was associated with prolonged disease-free survival (DFS) (44).

#### 4.6.2 Extracellular Matrix

The ECM not only controls many cellular events of cancer cells such as gene expression, proliferation, and metastatic invasiveness but also plays a crucial role in regulating cancer immunity (45, 46). It is also known that changes in ECM affect drug sensitivity in tumor cells (47). Furthermore, the expression of several cytokeratin genes such as *KRT5*, *KRT6B*, *KRT14*, and *KRT17* was associated with basal-like breast cancer, a breast cancer subtype that is highly associated with poor treatment outcome (48). Intriguingly, the KRT14 module of the chemosensitive network captures both *KRT5* and *KRT17* among these keratin genes (**Supplementary Figure S5**). The results were also consistent with KRT14 modules of both good and poor prognosis networks (**Supplementary Figure S6**). Moreover, the matrix Gla protein (*MGP*), which is a hub gene in the MGP module, uniquely found in the poor prognosis network (**Supplementary Figure S6**), has been reported to be among the genes that were upregulated in breast cancer cases where the prognosis was poor (49). Furthermore, other studies also provided evidence for lumican (LUM) in the COL3A1 module in the chemoresistant network (**Supplementary Figure S5**) and in the COL1A2-COL3A1 module in both good and poor prognosis networks (**Supplementary Figure S6**), where reduced expression of LUM was associated with dismal outcomes in node-negative invasive breast cancer (50). Also, carcinoembryonic antigen-related cell adhesion molecule (CEACAM5) included in the CEACAM6 module in the chemoresistant network (**Supplementary Figure S5**) and in the SCGB2A2–SCGB1D2 modules in the poor prognosis network (**Supplementary Figure S6**) was reported to be prognostic in both ER‐positive and ER‐negative breast cancer patients (51).

#### 4.6.3 Tumor Microenvironment

The tumor microenvironment (TME) is a milieu that affects the behaviors of cancer cells as well as the efficacy of antitumor treatments (52). Also, the microenvironment such as pH is known to affect the cellular activities of cancer cells. For instance, the hypoxic microenvironment of cancer cells could be one of the parameters that compromise chemosensitivity. Carbonic anhydrases that catalyze the reversible hydration of carbon dioxide to bicarbonate and a proton are enzymes essential to maintain pericellular pH homeostasis. Carbonic anhydrase 9 (CA9) was reported to be associated with chemosensitivity and prognosis in breast cancer patients treated with taxane and anthracycline (53). In our case, we found that CA2, another form of carbonic anhydrase, was found to be associated with the CPB1 module in the chemoresistant network (**Supplementary Figure S5**).

#### 4.6.4 Lipid Metabolism

Lipid intake, transport, and metabolism are known to play important roles in tumorigenesis and tumor immunity (54). For instance, lipid droplet formation was reported to be associated with prolonged breast cancer survival (55). Fatty acid-binding protein (FABP4) included in the FABP4 module in both chemoresistant (**Supplementary Figure S5**) and poor prognosis networks (**Supplementary Figure S6**) was detected in our encoded networks. Support for the role of FABP4 in chemoresistance and prognosis is implicated with the recent finding that the lipid droplet-associated protein perilipin 1 (PLIN1), which is found specifically associated with FABP4 modules in both the poor prognosis network (**Supplementary Figure S6**) and the chemoresistant network (**Supplementary Figure S5**), acts as a prognostic factor in breast cancer (56).

#### 4.6.5 Hemoglobin Status

Hemoglobin (Hb) levels had been reported by a number of studies to be associated with treatment outcomes and survival of patients of various cancer types (57–59) including breast cancer (60). It was reported that anemia in cancer patients is a prognostic factor associated with shorter survival (61). Further support for the role of Hb came from a study by Ikeda et al. which showed that hemoglobin subunit-β (HBB) was negatively associated with tumor reduction (62). Consistent with these findings, HBB was found as hub-specific to the chemosensitive (HBB-FABP4 module, **Supplementary Figure S5**) and good prognosis networks (NPY1R-HBB-FABP4 module, **Supplementary Figure S6**).

#### 4.6.6 Iron Metabolism

Iron is an essential element for heme biosynthesis and can act as an enzyme cofactor, for example, for ribonucleotide reductase that is involved in converting ribonucleotides to deoxyribonucleotides during DNA synthesis. High levels of iron are reported to be associated with tumor development (63), and a low iron intake diet has been suggested to reduce spontaneous mammary tumors in rats (64). Iron also can promote the production of reactive oxygen species during malignant transformation and affect tumor immunosurveillance (65). Miller et al. reported a 16-gene list of an iron regulatory gene signature that could predict outcome in breast cancer (66). Lactotransferrin (*LTF*), which was one of those genes, was found as a hub in the LTF-PIP and LTF modules in the good and poor prognosis networks (**Supplementary Figure S6**), respectively.

### 4.7 ANNE-Encoded Networks Also Uncover Novel Gene–Gene Associations

High expression levels of the protein G0/G1 Switch 2 (G0S2) which regulates lipid metabolism *via* peroxisome proliferator-activated receptor-α (PPARα) were recently reported to associate with a decrease in breast cancer recurrence rates (67). Interestingly, we found G0S2 to be associated with the apoptotic regulator BCL2A1 (also known as BFL1) *via* CXCL8 at the CXCL8 module specific to the chemosensitive network (**Supplementary Figure S5**). However, the functional association of G0S2 to the apoptotic regulator is lost in the poor prognosis network and instead is rewired to the lipid-binding protein FABP4 in the FABP4 module (**Supplementary Figure S6**). Our study, thus, suggests a novel mechanistic link between G0S2 and chemokine CXCL8 to promote drug-induced apoptosis.

Iseri et al. demonstrated that the ECM-associated genes *ITGA6*, *COL4A1*, *COL4A2*, *COL6A1*, *COL6A2*, *LAMA1*, *FN1*, *CLDN1*, *GPC6*, *SDC2*, *FBN1*, and *FBLN1* were significantly upregulated in doxorubicin-resistant MCF-7 breast cancer cell line (68). Intriguingly, our encoded chemosensitive network indicates negative functional associations between hemoglobin subunit-β (HBB) with COL3A1 and COL11A1 at the HBB-FABP4 module (**Supplementary Figure S5**), consistent with the need for high Hb and low level of collagen in promoting chemosensitivity. However, these Hb–collagen associations are not seen for the decoded chemoresistant network.

LGALS3BP, a member of the beta-galactoside-binding protein family implicated in modulating cell–cell and cell–matrix interactions, is associated with IFI27-related modules, which in turn is associated with several interferon-inducible proteins in all four ANNE-encoded networks (**Supplementary Figures S5**, **S6**). High levels of LGALS3BP in serum or tumor tissue of cancer patients were previously reported to be correlated with a poor survival or a more advanced disease in breast cancer (69). Our results suggest a general functional association of LGALS3BP to interferon-inducible proteins that regulate cell-mediated interactions in immune responses.

The above findings suggest that using the weight engineering approach, it is possible to decode novel cross-functional associations encoded as weight representations in autoencoders. Finding such novel functional gene–gene associations can shed new light in uncovering molecular crosstalk that regulates chemosensitivity and prognosis of breast cancers for future experimental endeavors to design novel therapeutics to better combat cancers.

## 5 Discussion

Studies from neuroscience revealed that the brain adjusts synaptic strengths between neurons during the learning process and stores information as memory in the form of a weighted connectome (70, 71). Knowledge is therefore stored as weight representations in the brain. ANNs learn by a similar mechanism and their applications have been deeply embedded in our daily life. Intriguingly, works from the past few years had revealed that ANNs, particularly autoencoders, are capable of storing meaningful information within hidden nodes (25–27). Such studies open a new avenue that ANNs can be used as knowledge discovery platforms. However, the promise of ANNs as knowledge discovery platforms and the use of the weight engineering approach to extract knowledge from encoded weight representations in ANNs are not explored.

In this study, we utilized an ANN autoencoder AI model and designed a novel weight engineering function to develop a knowledge discovery platform called ANNE. We used a breast cancer expression dataset with known clinical outcomes as our study case. We built and trained autoencoder models for a specific task and sought to decode learned weights into knowledge. To ensure that the reconstructed knowledge is meaningful, we robustly trained and explored various hyperparameters (activation function, number of nodes). Our models were also rigorously validated in a holdout procedure to ensure that there is no overfitting of the data. Overall, our procedures can ascertain that our platform has the desired functionalities for knowledge reconstruction.

Indeed, we found that several reconstructed gene–gene associations decoded by our weight engineering function have been previously reported in several studies. This demonstrates that the reconstructed gene associations and their respective networks indeed capture the clinical properties of breast cancer and hence can be considered as “knowledge” that explains the system (in this case, clinical traits of breast cancer). Furthermore, the directionality of reconstructed gene associations indicates the contribution of specific genes to the predictivity of the model which in turn provides mechanistic insights to understand which genes (or features) play more important roles in model performance. As such, we reason that these key gene associations can have an important contribution in conferring a given clinical trait (e.g., prognosis) in breast cancer.

Interestingly, we found that well-performing models trained with different sets of initialization parameters also encode biologically meaningful information. The stochastic nature of ANNs warrants some randomness, and like the human brain, no two individuals share exactly the same learning outcomes. This suggests that it is possible to construct multiple models that represent individualized learning and compile each of these “views” to generate a comprehensive picture of a system.

Although our current work uses an autoencoder with one hidden layer as a proof-of-concept study, the applicability of weight engineering to extract encoded knowledge is not limited to this autoencoder architecture. The gene–gene association scores of our weight engineering function (Equation 3) can be easily extended to multiple hidden layers.

It is important to note that the concept of knowledge discovery is distinct from model interpretability. ANNs are well-known for their black box properties because it is unknown how ANNs make predictions (72). Numerous efforts had been spent to develop explainable artificial intelligence (XAI) where the reasonings behind the predictions of AI algorithms can be traced and understood by humans (72, 73). Rule-based algorithms and decision trees are among the models that possess explanatory capabilities. Nonetheless, a few works had been conducted to modify ANN algorithms by incorporating specific knowledge domains to construct ANN architectures. Examples are visible neural networks (74) and biologically informed deep neural network (75) where connections from input to hidden nodes are guided by biological knowledge. Hence, it can be seen here that model interpretability is the inherent property of the design of the model. In contrast, knowledge discovery or knowledge extraction pertains to uncovering what has been learned by the model regardless of whether we understand how the models make a prediction. Here, knowledge refers to relationships between features that characterize the property of the subjects learned. One of the salient examples is our own brains. Although we still do not fully understand how our brains work to arrive at making a decision based on learned knowledge, we trust our brains for their learning capabilities, and in general, we know how knowledge is represented in the brains.

In conclusion, our work illustrates that meaningful knowledge is indeed stored as weight representations in autoencoders. This concept alone is novel because interneuronal weights in ANNs are not just mere learned parameters to fit the data for a predictive task but possess a meaningful representation of knowledge pertaining to the data. Unlike conventional knowledge extraction which focuses on identifying important features, knowledge reconstructed from weight engineering approaches reveals how these features are associated, and to a certain extent, provides deeper mechanistic insights that can explain the properties of a disease. Knowledge on associations between gene–gene pairs can in turn offer novel insights on how a biological system can be better manipulated to devise novel therapeutic options. We show that ANNE has the power to uncover immune-related processes that underpin breast cancer clinical traits where ANNE-reconstructed gene association networks can provide mechanistic insights into their actions. In summary, our weight engineering approach has vast applicability and opens new avenues to employ AI-based knowledge discovery platforms.

## Data Availability Statement

Publicly available datasets were analyzed in this study. This data can be found here: GSE25066; https://www.ncbi.nlm.nih.gov/geo/query/acc.cgi?acc=gse25066.

## Author Contributions

CZ, CC, CU, and HL contributed to the conception and design of the study. CZ, CU, and HL contributed to the acquisition of data. CZ, CC, TW, ST, KM-L, GY, JY, KY, SZ, CU, and HL contributed to the analysis and interpretation of data. CZ, CC, CU, and HL drafted the manuscript. CU and HL supervised the study. All authors contributed to the article and approved the submitted version.

## Funding

This work was supported by grants from the National Institutes of Health (NIH) (R01AG056318, R01AG61796, R01CA240323, P50CA136393, P01CA229100), the Glenn Foundation for Medical Research, Mayo Clinic DERIVE Office and Mayo Clinic Center for Biomedical Discovery, Mayo Clinic Center for Individualized Medicine, Mayo Clinic Cancer Center (P30CA015083), and the David F. and Margaret T. Grohne Cancer Immunology and Immunotherapy Program.

## Conflict of Interest

The authors declare that the research was conducted in the absence of any commercial or financial relationships that could be construed as a potential conflict of interest.

## Publisher’s Note

All claims expressed in this article are solely those of the authors and do not necessarily represent those of their affiliated organizations, or those of the publisher, the editors and the reviewers. Any product that may be evaluated in this article, or claim that may be made by its manufacturer, is not guaranteed or endorsed by the publisher.

## Supplementary Material

Supplementary Figure 1 Encoded chemosensitive (pCR) network from top 200 gene pairs of absolute association scores. Major biological processes from Gene Ontology are highlighted with their respective colors.

Supplementary Figure 2 Encoded chemoresistant (RD) network from top 200 gene pairs of absolute association scores. Major biological processes from Gene Ontology are highlighted with their respective colors.

Supplementary Figure 3 Encoded good prognosis network from top 200 gene pairs of absolute association scores. Major biological processes from Gene Ontology are highlighted with their respective colors.

Supplementary Figure 4 Encoded poor prognosis network from top 200 gene pairs of absolute association scores. Major biological processes from Gene Ontology are highlighted with their respective colors.

Supplementary Figure 5 Common network modules for encoded chemosensitive and chemoresistant networks. Modules are named based on their respective hub genes. Chemosensitive modules are shown with nodes of green borders and chemoresistant modules are shown with nodes of grey borders. Red edges between nodes indicate the positive cumulative association weights. Direction of arrows shown in the edges indicates predictability of a gene to its counterpart. For instance, X→Y indicate expression of X from the input layer predicts the expression of Y at the output layer.

Supplementary Figure 6 Common network modules for encoded good and poor prognosis networks. Modules are named based on their respective hub genes. Modules of good prognosis network are shown with nodes of green borders and modules of poor prognosis network are shown with nodes of grey borders. Red and blue edges between nodes indicate positive and negative cumulative association weights, respectively. Direction of arrows shown in the edges indicate predictability of a gene to its counterpart.

Supplementary Figure 7 Gene expression profiles (z-scores) for hub genes and their associated genes in ANNE-encoded networks. **(A)** Chemosensitivity networks (pCR: chemosensitive; RD: chemoresistant). **(B)** Prognosis networks.

Supplementary Data Sheet 1 Gene Set Enrichment Analyses (GSEA) for models trained with different sets of initialization parameters. Models were trained with linear activation function and 50 hidden layer nodes for each of the sample subsets pCR, RD, good, and poor prognosis. For each subset, 10 models initialized with different random seeds were trained and analyzed. For each model, 50 ranked gene lists containing the weight between the hidden layer nodes to input layer were generated and used as input for GSEA against the MSigDB database. Genesets with false discovery rate (FDR) < 0.05 were deemed enriched pathways. Tables 1A through 1d indicate the genesets that appeared in at least 1 hidden layer node of all 10 models with different random seeds as well as in the model further discussed in this work (the “reference model”), i.e., these gene sets are common to all these models. Tables 2A through 2d show the gene sets that appear in the “reference model”, and in at least 1 of the 10 models with different random seeds. The number in the cells indicates the number of occurrences of hidden layer nodes enriched for a given pathway.

## References

[1] Graw F. Deciphering the Triad of Infection, Immunity and Pathology. Elife (2021) 10:1–3. doi: 10.7554/eLife.72379 PMC8410069 34468313

[2] Jevtic S Sengar AS Salter MW McLaurin J. The Role of the Immune System in Alzheimer Disease: Etiology and Treatment. Ageing Res Rev (2017) 40:84–94. doi: 10.1016/j.arr.2017.08.005 28941639

[3] Blagih J Buck MD Vousden KH. P53, Cancer and the Immune Response. J Cell Sci (2020) 133:1–13. doi: 10.1242/jcs.237453 32144194

[4] Hiam-Galvez KJ Allen BM Spitzer MH. Systemic Immunity in Cancer. Nat Rev Cancer (2021) 21:345–59. doi: 10.1038/s41568-021-00347-z PMC8034277 33837297

[5] Lamprecht R LeDoux J. Structural Plasticity and Memory. Nat Rev Neurosci (2004) 5:45–54. doi: 10.1038/nrn1301 14708003

[6] Caroni P Donato F Muller D. Structural Plasticity Upon Learning: Regulation and Functions. Nat Rev Neurosci (2012) 13:478–90. doi: 10.1038/nrn3258 22714019

[7] Caroni P Chowdhury A Lahr M. Synapse Rearrangements Upon Learning: From Divergent-Sparse Connectivity to Dedicated Sub-Circuits. Trends Neurosci (2014) 37:604–14. doi: 10.1016/j.tins.2014.08.011 25257207

[8] Frankland PW Bontempi B. The Organization of Recent and Remote Memories. Nat Rev Neurosci (2005) 6:119–30. doi: 10.1038/nrn1607 15685217

[9] Barth AL Poulet JFA. Experimental Evidence for Sparse Firing in the Neocortex. Trends Neurosci (2012) 35:345–55. doi: 10.1016/j.tins.2012.03.008 22579264

[10] Hromadka T Deweese MR Zador AM. Sparse Representation of Sounds in the Unanesthetized Auditory Cortex. PloS Biol (2008) 6:e16. doi: 10.1371/journal.pbio.0060016 18232737 PMC2214813

[11] Vinje WE Gallant JL. Sparse Coding and Decorrelation in Primary Visual Cortex During Natural Vision. Science (2000) 287:1273–6. doi: 10.1126/science.287.5456.1273 10678835

[12] Crochet S Poulet JF Kremer Y Petersen CC. Synaptic Mechanisms Underlying Sparse Coding of Active Touch. Neuron (2011) 69:1160–75. doi: 10.1016/j.neuron.2011.02.022 21435560

[13] McCulloch WS Pitts W. A Logical Calculus of the Ideas Immanent in Nervous Activity 1943. Bull Math Biol (1990) 52:99–115. doi: 10.1016/S0092-8240(05)80006-0 2185863

[14] Hebb DO. The Organization of Behavior. A Neuropschychological Theory. United States: John Wiley & Sons (1949).

[15] Rosenblatt F. The Perceptron: A Probabilistic Model for Information Storage and Organization in the Brain. Psychol Rev (1958) 65:386–408. doi: 10.1037/h0042519 13602029

[16] Fukushima K. Neocognitron: A Self Organizing Neural Network Model for a Mechanism of Pattern Recognition Unaffected by Shift in Position. Biol Cybern (1980) 36:193–202. doi: 10.1007/BF00344251 7370364

[17] Eberhart RC Dobbins RW. Early Neural Network Development History: The Age of Camelot. IEEE Eng Med Biol Mag (1990) 9:15–8. doi: 10.1109/51.59207 18238341

[18] LeCun Y Bengio Y Hinton G. Deep Learning. Nature (2015) 521:436–44. doi: 10.1038/nature14539 26017442

[19] Tao W Wu DJ Coates A Ng AY. (2012). End-To-End Text Recognition With Convolutional Neural Networks, in: IEEE Proceedings of the 21st International Conference on Pattern Recognition ICPR2012, 3304-3308. End-to-End Text Recognition With Convolutional Neural Networks, Japan: IEEE Proceedings of the 21st International Conference on Pattern Recognition ICPR2012.

[20] Esteva A Robicquet A Ramsundar B Kuleshov V DePristo M Chou K. A Guide to Deep Learning in Healthcare. Nat Med (2019) 25:24–9. doi: 10.1038/s41591-018-0316-z 30617335

[21] Silver D Schrittwieser J Simonyan K Antonoglou I Huang A Guez A. Mastering the Game of Go Without Human Knowledge. Nature (2017) 550:354–9. doi: 10.1038/nature24270 29052630

[22] Senior AW Evans R Jumper J Kirkpatrick J Sifre L Green T. Improved Protein Structure Prediction Using Potentials From Deep Learning. Nature (2020) 577:706–10. doi: 10.1038/s41586-019-1923-7 31942072

[23] Luo P Li Y Tian LP Wu FX. Enhancing the Prediction of Disease-Gene Associations With Multimodal Deep Learning. Bioinformatics (2019) 35:3735–42. doi: 10.1093/bioinformatics/btz155 30825303

[24] Chen H Engkvist O Wang Y Olivecrona M Blaschke T. The Rise of Deep Learning in Drug Discovery. Drug Discov Today (2018) 23:1241–50. doi: 10.1016/j.drudis.2018.01.039 29366762

[25] Way GP Greene CS. Extracting a Biologically Relevant Latent Space From Cancer Transcriptomes With Variational Autoencoders. Pac Symp Biocomput (2018) 23:80–91. doi: 10.1142/9789813235533_0008 29218871 PMC5728678

[26] Tan J Hammond JH Hogan DA Greene CS. ADAGE-Based Integration of Publicly Available Pseudomonas Aeruginosa Gene Expression Data With Denoising Autoencoders Illuminates Microbe-Host Interactions. mSystems (2016) 1:1–17. doi: 10.1128/mSystems.00025-15 PMC5069748 27822512

[27] Tan J Ung M Cheng C Greene CS. Unsupervised Feature Construction and Knowledge Extraction From Genome-Wide Assays of Breast Cancer With Denoising Autoencoders. Pac Symp Biocomput (2015) 132–43. doi: 10.1142/9789814644730_0014 PMC4299935 25592575

[28] Hinton GE Salakhutdinov RR. Reducing the Dimensionality of Data With Neural Networks. Science (2006) 313:504–7. doi: 10.1126/science.1127647 16873662

[29] Hatzis C Pusztai L Valero V Booser DJ Esserman L Lluch A. A Genomic Predictor of Response and Survival Following Taxane-Anthracycline Chemotherapy for Invasive Breast Cancer. JAMA (2011) 305:1873–81. doi: 10.1001/jama.2011.593 PMC5638042 21558518

[30] Piccolo SR Sun Y Campbell JD Lenburg ME Bild AH Johnson WE. A Single-Sample Microarray Normalization Method to Facilitate Personalized-Medicine Workflows. Genomics (2012) 100:337–44. doi: 10.1016/j.ygeno.2012.08.003 PMC3508193 22959562

[31] Piccolo SR Withers MR Francis OE Bild AH Johnson WE. Multiplatform Single-Sample Estimates of Transcriptional Activation. Proc Natl Acad Sci USA (2013) 110:17778–83. doi: 10.1073/pnas.1305823110 PMC3816418 24128763

[32] He K Zhang X Ren S Sun J. Delving Deep Into Rectiﬁers: Surpassing Human-Level Performance on ImageNet Classiﬁcation. arXiv (2015)arXiv:1502.01852v1.

[33] Andersen P Gill RD. Cox's Regression Model for Counting Processes, a Large Sample Study. Ann Stat (1982) 10:1100–20. doi: 10.1214/aos/1176345976

[34] Therneau T Grambsch P. Modeling Survival Data: Extending the Cox Model. Germany: Springer-Verlag (2000).

[35] Subramanian A Tamayo P Mootha VK Mukherjee S Ebert BL Gillette MA. Gene Set Enrichment Analysis: A Knowledge-Based Approach for Interpreting Genome-Wide Expression Profiles. Proc Natl Acad Sci USA (2005) 102:15545–50. doi: 10.1073/pnas.0506580102 PMC1239896 16199517

[36] Mootha VK Lindgren CM Eriksson K Subramanian A Sihag S Lehar J. PGC-1alpha-Responsive Genes Involved in Oxidative Phosphorylation are Coordinately Downregulated in Human Diabetes. Nat Genet (2003) 34:267–73. doi: 10.1038/ng1180 12808457

[37] Liberzon A Subramanian A Pinchback R Thorvaldsdóttir H Tamayo P Mesirov JP. Molecular Signatures Database MSigDB 3.0. Bioinformatics (2011) 27:1739–40. doi: 10.1093/bioinformatics/btr260 PMC3106198 21546393

[38] Bresnick AR Weber DJ Zimmer DB. S100 Proteins in Cancer. Nat Rev Cancer (2015) 15:96–109. doi: 10.1038/nrc3893 25614008 PMC4369764

[39] Kroemer G Senovilla L Galluzzi L Andre F Zitvogel L. Natural and Therapy-Induced Immunosurveillance in Breast Cancer. Nat Med (2015) 21:1128–38. doi: 10.1038/nm.3944 26444637

[40] Zitvogel L Apetoh L Ghiringhelli F Kroemer G. Immunological Aspects of Cancer Chemotherapy. Nat Rev Immunol (2008) 8:59–73. doi: 10.1038/nri2216 18097448

[41] Stoll G Enot D Mlecnik B Galon J Zitvogel L Kroemer G. Immune-Related Gene Signatures Predict the Outcome of Neoadjuvant Chemotherapy. Oncoimmunology (2014) 3:e27884. doi: 10.4161/onci.27884 24790795 PMC4004621

[42] Denkert C von Minckwitz G Darb-Esfahani S Lederer B Heppner BI Weber KE. Tumor-Infiltrating Lymphocytes and Response to Neoadjuvant Chemotherapy With or Without Carboplatin in Human Epidermal Growth Factor Receptor 2-Positive and Triple-Negative Primary Breast Cancers. J Clin Oncol (2015) 33:983–91. doi: 10.1200/JCO.2014.58.1967 25534375

[43] Rastogi P Anderson SJ Bear HD Geyer CE Kahlenberg MS Robidoux A. Preoperative Chemotherapy: Updates of National Surgical Adjuvant Breast and Bowel Project Protocols B-18 and B-27. J Clin Oncol (2008) 26:778–85. doi: 10.1200/JCO.2007.15.0235 18258986

[44] Specht K Harbeck N Smida J Annecke K Reich U Naehrig J. Expression Profiling Identifies Genes That Predict Recurrence of Breast Cancer After Adjuvant CMF-Based Chemotherapy. Breast Cancer Res Treat (2009) 118:45–56. doi: 10.1007/s10549-008-0207-y 18925433

[45] Gordon-Weeks A Yuzhalin AE. Cancer Extracellular Matrix Proteins Regulate Tumour Immunity. Cancers Basel (2020) 12:1–25. doi: 10.3390/cancers12113331 PMC7696558 33187209

[46] He Y Liu T Dai S Xu Z Wang L Luo F. Tumor-Associated Extracellular Matrix: How to Be a Potential Aide to Anti-Tumor Immunotherapy? Front Cell Dev Biol (2021) 9:739161. doi: 10.3389/fcell.2021.739161 34733848 PMC8558531

[47] Morin PJ. Drug Resistance and the Microenvironment: Nature and Nurture. Drug Resist Update (2003) 6:169–72. doi: 10.1016/s1368-76460300059-1 12962682

[48] Rakha EA Reis-Filho JS Ellis IO. Basal-Like Breast Cancer: A Critical Review. J Clin Oncol (2008) 26:2568–81. doi: 10.1200/JCO.2007.13.1748 18487574

[49] Yoshimura K Takeuchi K Nagasaki K Ogishima S Tanaka H Iwase T. Prognostic Value of Matrix Gla Protein in Breast Cancer. Mol Med Rep (2009) 2:549–53. doi: 10.3892/mmr_00000135 21475864

[50] Troup S Njue C Kliewer EV Parisien M Roskelley C Chakravarti S. Reduced Expression of the Small Leucine-Rich Proteoglycans, Lumican, and Decorin is Associated With Poor Outcome in Node-Negative Invasive Breast Cancer. Clin Cancer Res (2003) 9:207–14. 12538471

[51] Ring BZ Seitz RS Beck R Shasteen WJ Tarr SM Cheang MCU. Novel Prognostic Immunohistochemical Biomarker Panel for Estrogen Receptor-Positive Breast Cancer. J Clin Oncol (2006) 24:3039–47. doi: 10.1200/JCO.2006.05.6564 16809728

[52] Labani-Motlagh A Ashja-Mahdavi M Loskog A. The Tumor Microenvironment: A Milieu Hindering and Obstructing Antitumor Immune Responses. Front Immunol (2020) 11:940. doi: 10.3389/fimmu.2020.00940 32499786 PMC7243284

[53] Aomatsu N Yashiro M Kashiwagi S Kawajiri H Takashima T Ohsawa M. Carbonic Anhydrase 9 is Associated With Chemosensitivity and Prognosis in Breast Cancer Patients Treated With Taxane and Anthracycline. BMC Cancer (2014) 14:400. doi: 10.1186/1471-2407-14-400 24893880 PMC4058694

[54] Prendeville H Lynch L. Diet, Lipids, and Antitumor Immunity. Cell Mol Immunol (2022) 19:432–44. doi: 10.1038/s41423-021-00781-x PMC8891265 34983949

[55] Pucer A Brglez V Payré C Pungerčar J Lambeau G Petan T. Group X Secreted Phospholipase A2 Induces Lipid Droplet Formation and Prolongs Breast Cancer Cell Survival. Mol Cancer (2013) 12:111. doi: 10.1186/1476-4598-12-111 24070020 PMC3852912

[56] Jung YY Kim HM Koo JS. Expression of Lipid Metabolism-Related Proteins in Metastatic Breast Cancer. PloS One (2015) 10:e0137204. doi: 10.1371/journal.pone.0137204 26334757 PMC4559312

[57] Tarnawski R Skladowski K Maciejewski B. Prognostic Value of Hemoglobin Concentration in Radiotherapy for Cancer of Supraglottic Larynx. Int J Radiat Oncol Biol Phys (1997) 38:1007–11. doi: 10.1016/s0360-30169700308-8 9276366

[58] Hamai Y Hihara J Taomoto J Yamakita I Ibuki Y Okada M. Hemoglobin Level Influences Tumor Response and Survival After Neoadjuvant Chemoradiotherapy for Esophageal Squamous Cell Carcinoma. World J Surg (2014) 38:2046–51. doi: 10.1007/s00268-014-2486-2 24615604

[59] Ye X Liu J Chen Y Wang N Lu R. The Impact of Hemoglobin Level and Transfusion on the Outcomes of Chemotherapy in Gastric Cancer Patients. Int J Clin Exp Med (2015) 8:4228–35. PMC4443168 26064334

[60] Lee CL Tsai CH Yeh DC Lin CS Li YF Tzeng HE. Hemoglobin Level Trajectories in the Early Treatment Period are Related With Survival Outcomes in Patients With Breast Cancer. Oncotarget (2017) 8:1569–79. doi: 10.18632/oncotarget.13679 PMC5352078 27906669

[61] Caro JJ Salas M Ward A Goss G. Anemia as an Independent Prognostic Factor for Survival in Patients With Cancer: A Systemic, Quantitative Review. Cancer (2001) 91:2214–21. doi: 10.1002/1097-0142(20010615)91:12<2214::AID-CNCR1251>3.0.CO;2-P 11413508

[62] Ikeda T Jinno H Shirane M. Chemosensitivity-Related Genes of Breast Cancer Detected by DNA Microarray. Anticancer Res (2007) 27:2649–55. 17695428

[63] Stevens RG Graubard BI Micozzi MS Neriishi K Blumberg BS. Moderate Elevation of Body Iron Level and Increased Risk of Cancer Occurrence and Death. Int J Cancer (1994) 56:364–9. doi: 10.1002/ijc.2910560312 8314323

[64] Hann HW Stahlhut MW Menduke H. Iron Enhances Tumor Growth. Observation on Spontaneous Mammary Tumors in Mice. Cancer (1991) 68:2407–10. doi: 10.1002/1097-01421991120168:11<2407::aid-cncr2820681113>3.0.co;2-n 1657354

[65] Sacco A Battaglia AM Botta C Aversa I Mancuso S Costanzo F. Iron Metabolism in the Tumor Microenvironment-Implications for Anti-Cancer Immune Response. Cells (2021) 10:1–17. doi: 10.3390/cells10020303 PMC7913036 33540645

[66] Miller LD Coffman LG Chou JW Black MA Bergh J D'Agostino R Jr. An Iron Regulatory Gene Signature Predicts Outcome in Breast Cancer. Cancer Res (2011) 71:6728–37. doi: 10.1158/0008-5472.CAN-11-1870 PMC3206152 21875943

[67] Yim CY Sekula DJ Hever-Jardine MP Liu X Warzecha JM Tam J. G0S2 Suppresses Oncogenic Transformation by Repressing a MYC-Regulated Transcriptional Program. Cancer Res (2016) 76:1204–13. doi: 10.1158/0008-5472.CAN-15-2265 PMC4775337 26837760

[68] Iseri OD Kars MD Arpaci F Gunduz U. Gene Expression Analysis of Drug-Resistant MCF-7 Cells: Implications for Relation to Extracellular Matrix Proteins. Cancer Chemother Pharmacol (2010) 65:447–55. doi: 10.1007/s00280-009-1048-z 19543729

[69] Tinari N Lattanzio R Querzoli P Natoli C Grassadonia A Alberti S. High Expression of 90K Mac-2 BP is Associated With Poor Survival in Node-Negative Breast Cancer Patients Not Receiving Adjuvant Systemic Therapies. Int J Cancer (2009) 124:333–8. doi: 10.1002/ijc.23970 18942707

[70] Eichler K Li F Litwin-Kumar A Park Y Andrade I Schneider-Mizell CM. The Complete Connectome of a Learning and Memory Centre in an Insect Brain. Nature (2017) 548:175–82. doi: 10.1038/nature23455 PMC5806122 28796202

[71] Jarrell TA Wang Y Bloniarz AE Brittin CA Xu M Nichol Thomson J. The Connectome of a Decision-Making Neural Network. Science (2012) 337:437–44. doi: 10.1126/science.1221762 22837521

[72] Castelvecchi D. Can We Open the Black Box of AI? Nature (2016) 538:20–3. doi: 10.1038/538020a 27708329

[73] Došilović FK Brčić M Hlupić N. Explainable Artificial Intelligence: A Survey. IEEE MIPRO (2018), 210–15.

[74] Ma J Yu MK Fong S Ono K Sage E Demchak B. Using Deep Learning to Model the Hierarchical Structure and Function of a Cell. Nat Methods (2018) 15:290–8. doi: 10.1038/nmeth.4627 PMC5882547 29505029

[75] Elmarakeby HA Hwang J Arafeh R Crowdis J Gang S David L. Biologically Informed Deep Neural Network for Prostate Cancer Discovery. Nature (2021) 598:348–52. doi: 10.1038/s41586-021-03922-4 PMC8514339 34552244

