# Supplementary figures and images for: A Knowledge-Based Discovery Approach Couples Artificial Neural Networks With Weight Engineering to Uncover Immune-Related Processes Underpinning Clinical Traits of Breast Cancer
Source: Front Immunol. 2022 Jul 14;13:920669. doi: 10.3389/fimmu.2022.920669 (PMC9330471; doi:10.3389/fimmu.2022.920669)

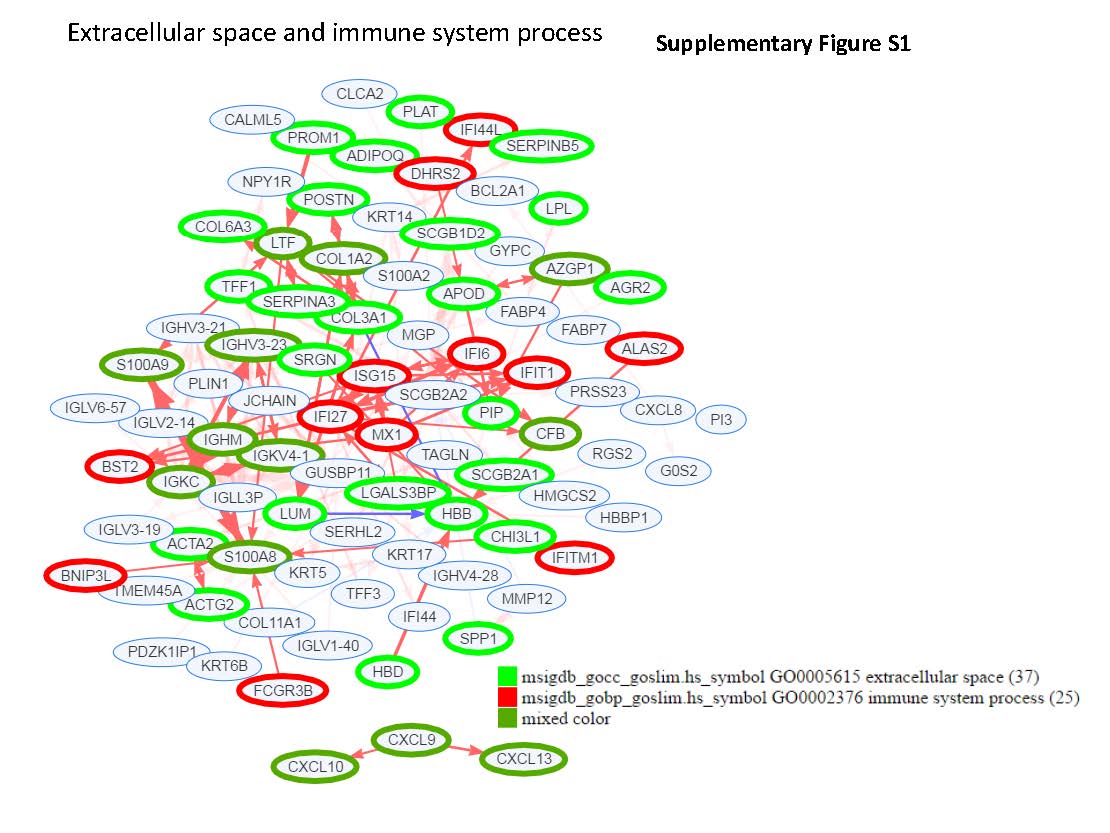

Supplement: Supplementary Figure 1 — Encoded chemosensitive (pCR) network from top 200 gene pairs of absolute association scores. Major biological processes from Gene Ontology are highlighted with their respective colors. [file DataSheet_2.zip › ANNE_Supplementary Figures S1-S7_Page_01.jpg]

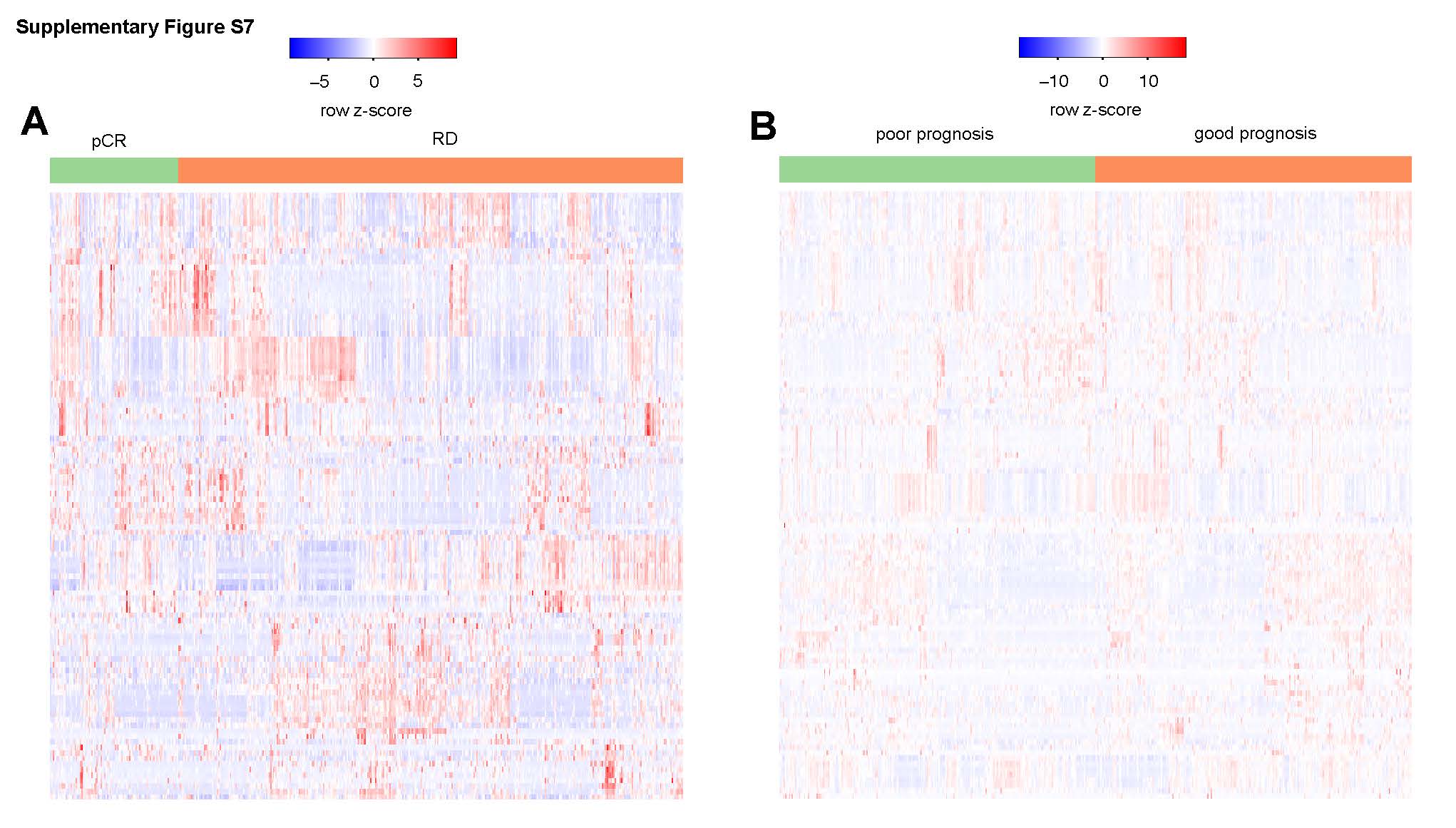

Supplement: Supplementary Figure 1 — Encoded chemosensitive (pCR) network from top 200 gene pairs of absolute association scores. Major biological processes from Gene Ontology are highlighted with their respective colors. [file DataSheet_2.zip › ANNE_Supplementary Figures S1-S7_Page_63.jpg]

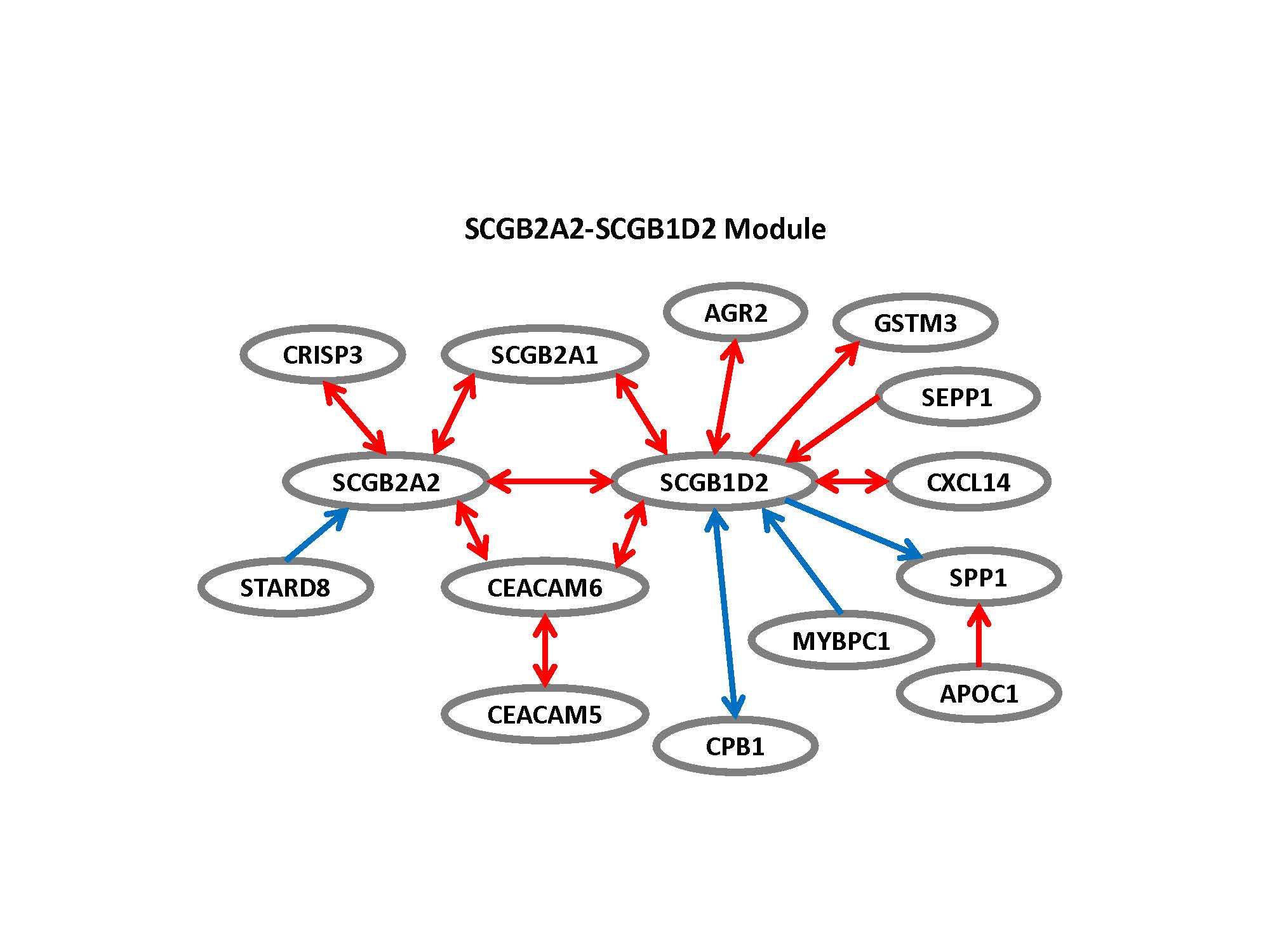

Supplement: Supplementary Figure 1 — Encoded chemosensitive (pCR) network from top 200 gene pairs of absolute association scores. Major biological processes from Gene Ontology are highlighted with their respective colors. [file DataSheet_2.zip › ANNE_Supplementary Figures S1-S7_Page_62.jpg]

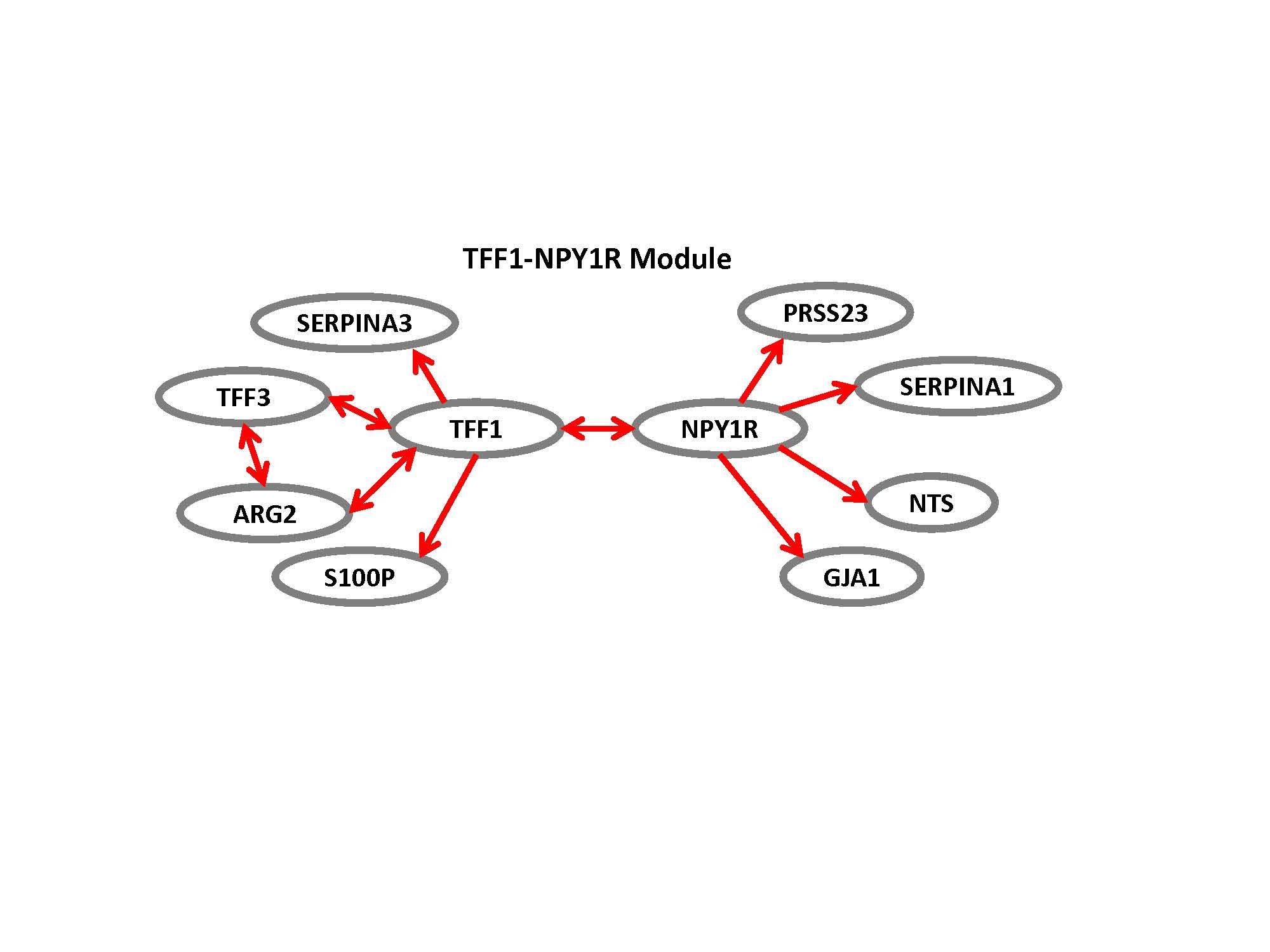

Supplement: Supplementary Figure 1 — Encoded chemosensitive (pCR) network from top 200 gene pairs of absolute association scores. Major biological processes from Gene Ontology are highlighted with their respective colors. [file DataSheet_2.zip › ANNE_Supplementary Figures S1-S7_Page_61.jpg]

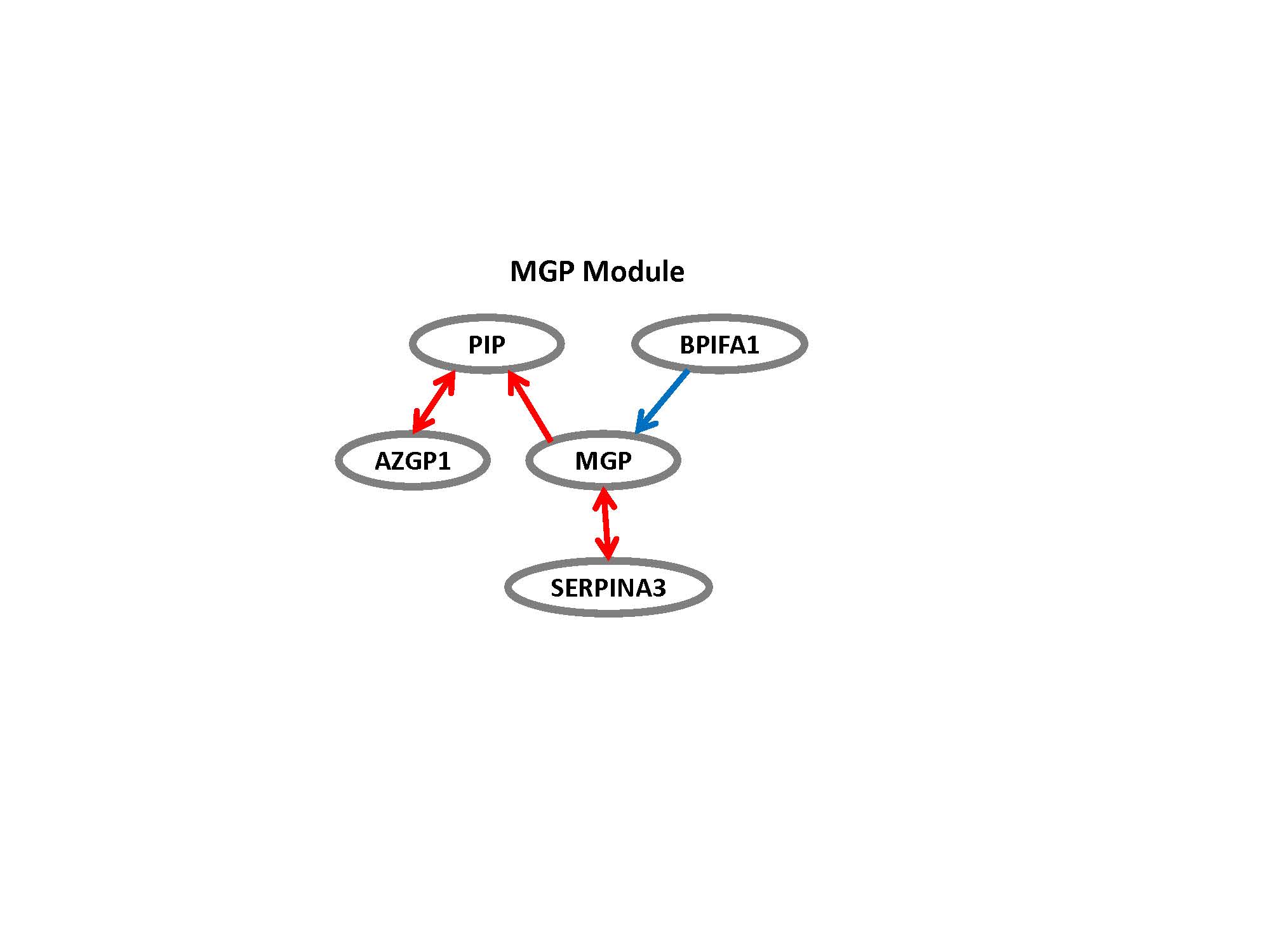

Supplement: Supplementary Figure 1 — Encoded chemosensitive (pCR) network from top 200 gene pairs of absolute association scores. Major biological processes from Gene Ontology are highlighted with their respective colors. [file DataSheet_2.zip › ANNE_Supplementary Figures S1-S7_Page_60.jpg]

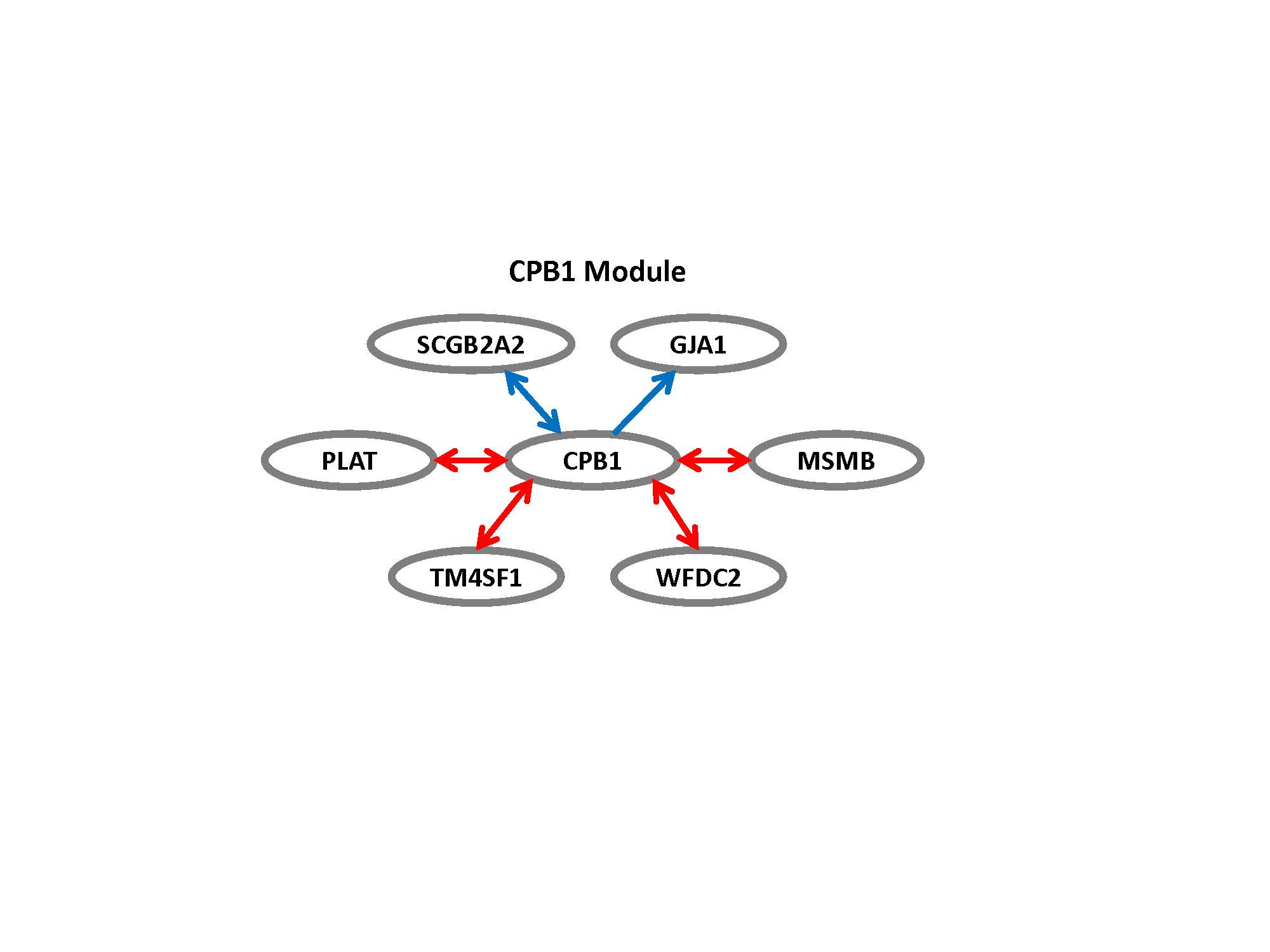

Supplement: Supplementary Figure 1 — Encoded chemosensitive (pCR) network from top 200 gene pairs of absolute association scores. Major biological processes from Gene Ontology are highlighted with their respective colors. [file DataSheet_2.zip › ANNE_Supplementary Figures S1-S7_Page_59.jpg]

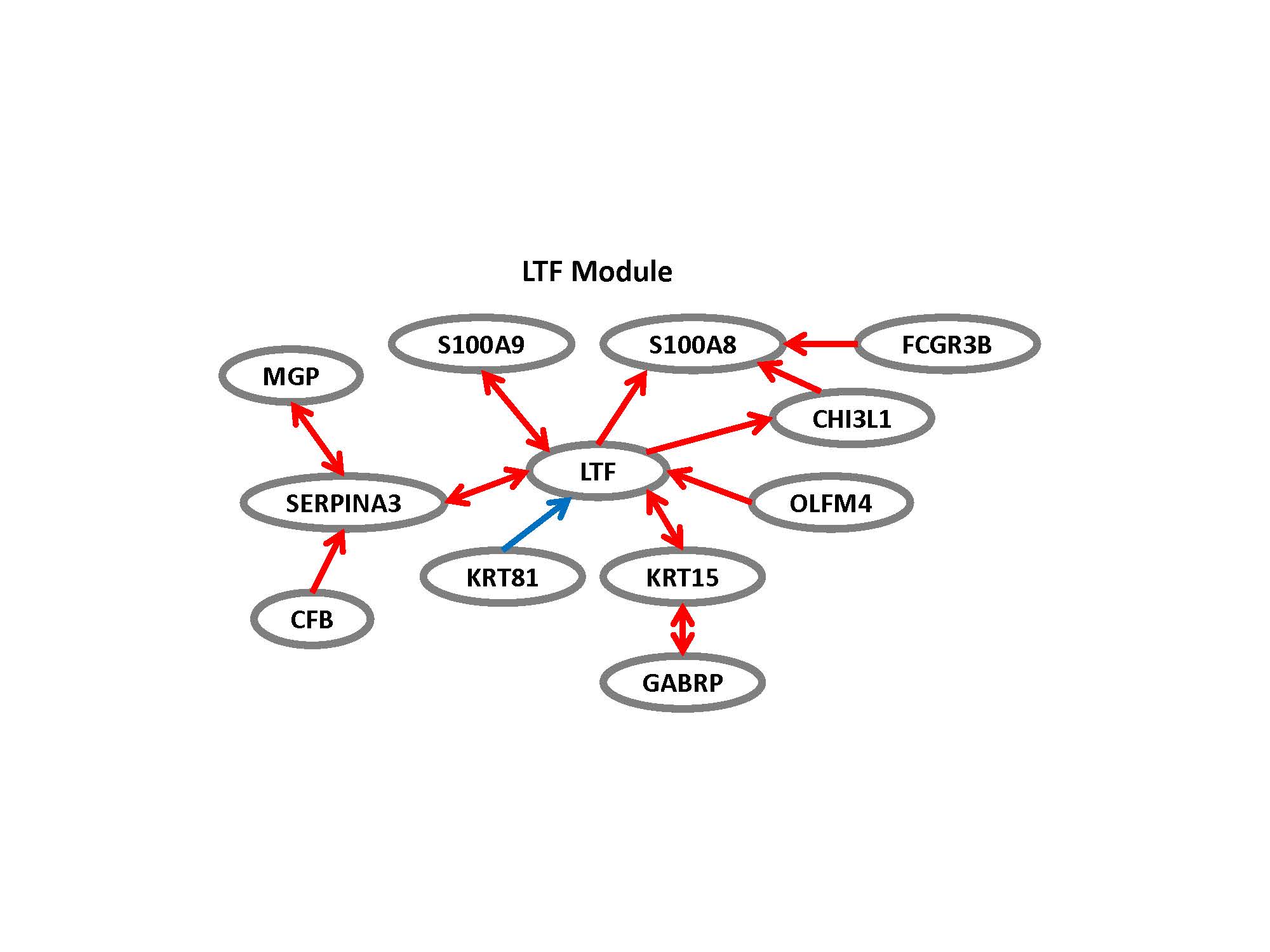

Supplement: Supplementary Figure 1 — Encoded chemosensitive (pCR) network from top 200 gene pairs of absolute association scores. Major biological processes from Gene Ontology are highlighted with their respective colors. [file DataSheet_2.zip › ANNE_Supplementary Figures S1-S7_Page_58.jpg]

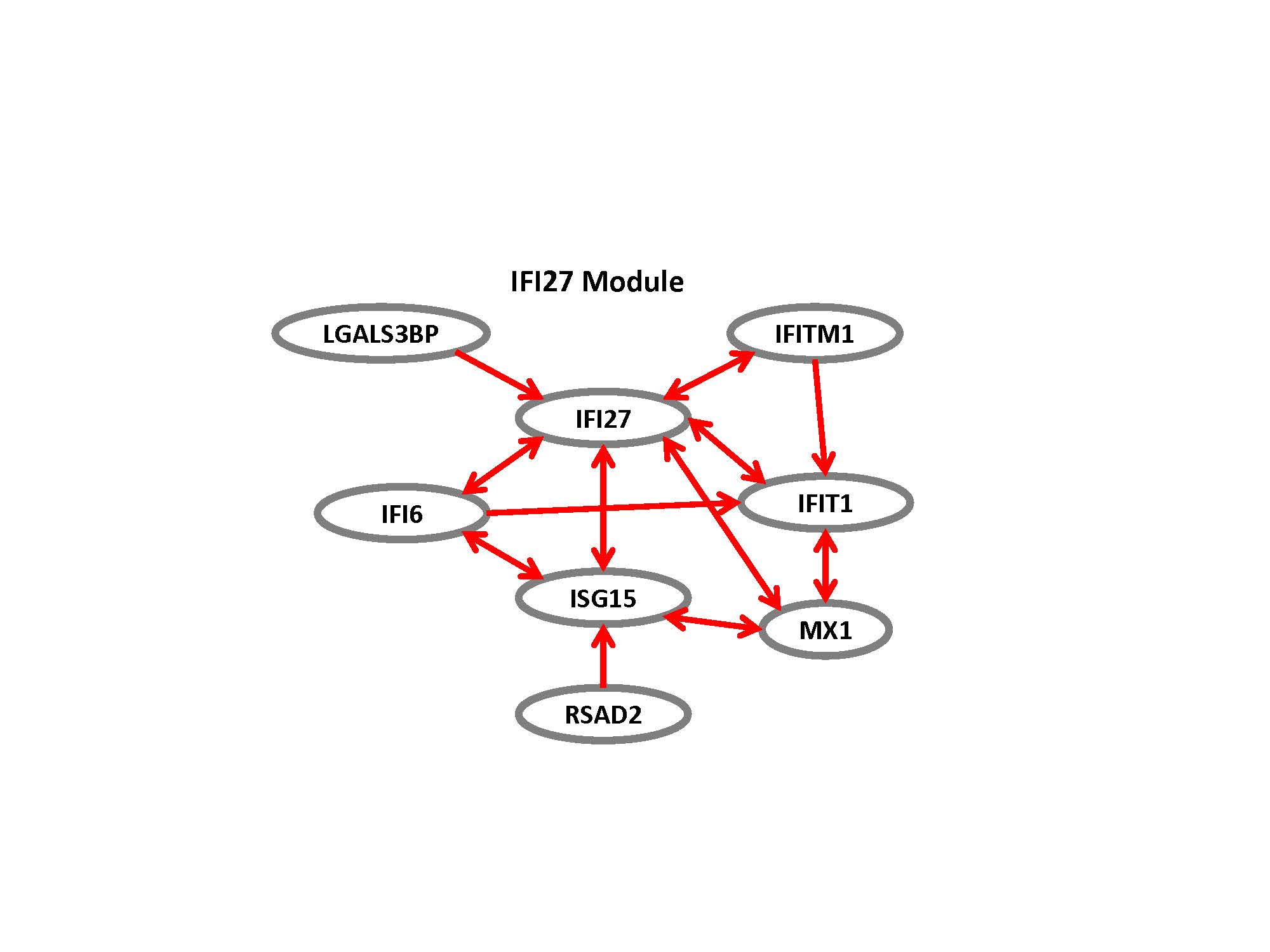

Supplement: Supplementary Figure 1 — Encoded chemosensitive (pCR) network from top 200 gene pairs of absolute association scores. Major biological processes from Gene Ontology are highlighted with their respective colors. [file DataSheet_2.zip › ANNE_Supplementary Figures S1-S7_Page_57.jpg]

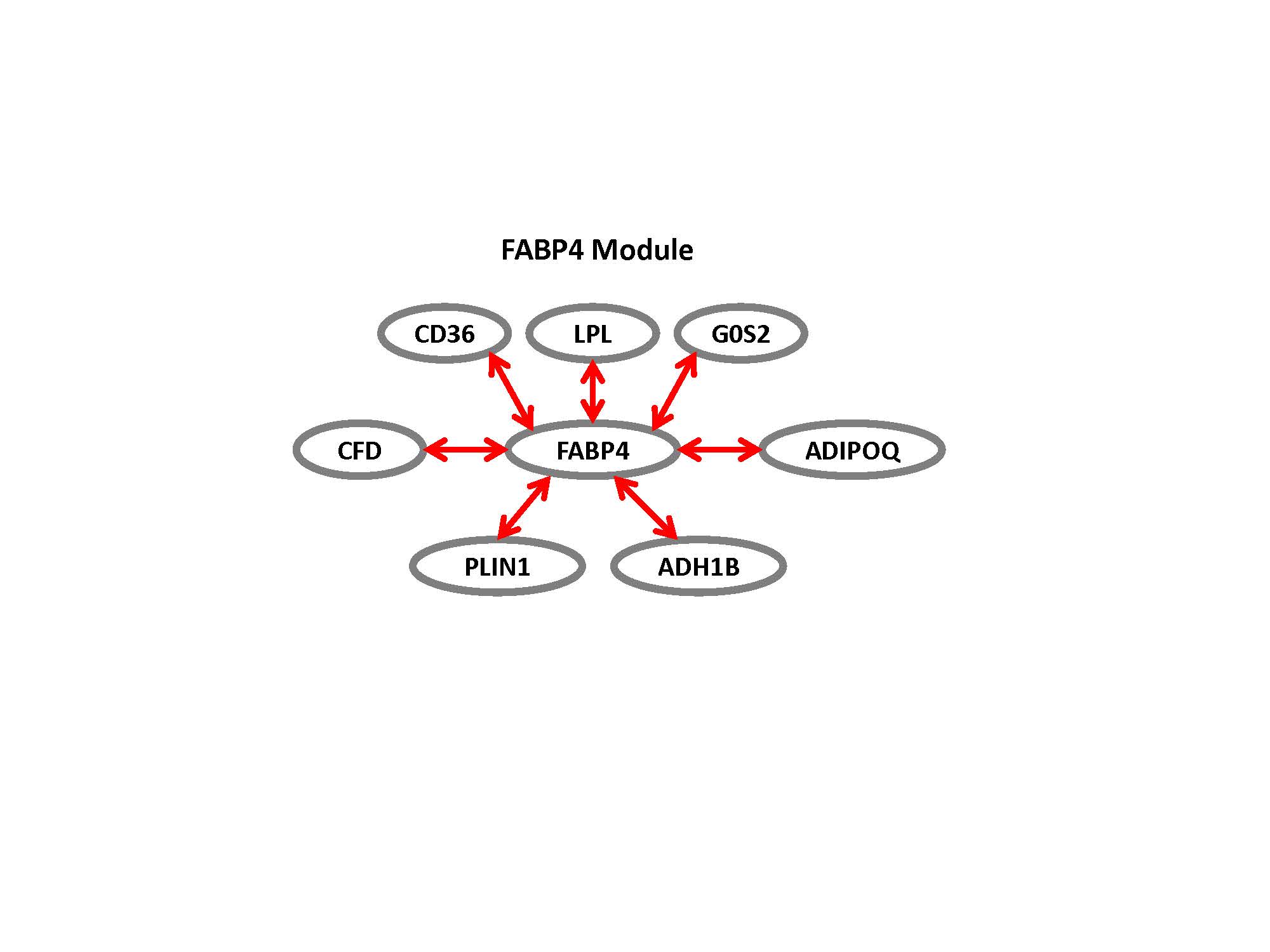

Supplement: Supplementary Figure 1 — Encoded chemosensitive (pCR) network from top 200 gene pairs of absolute association scores. Major biological processes from Gene Ontology are highlighted with their respective colors. [file DataSheet_2.zip › ANNE_Supplementary Figures S1-S7_Page_56.jpg]

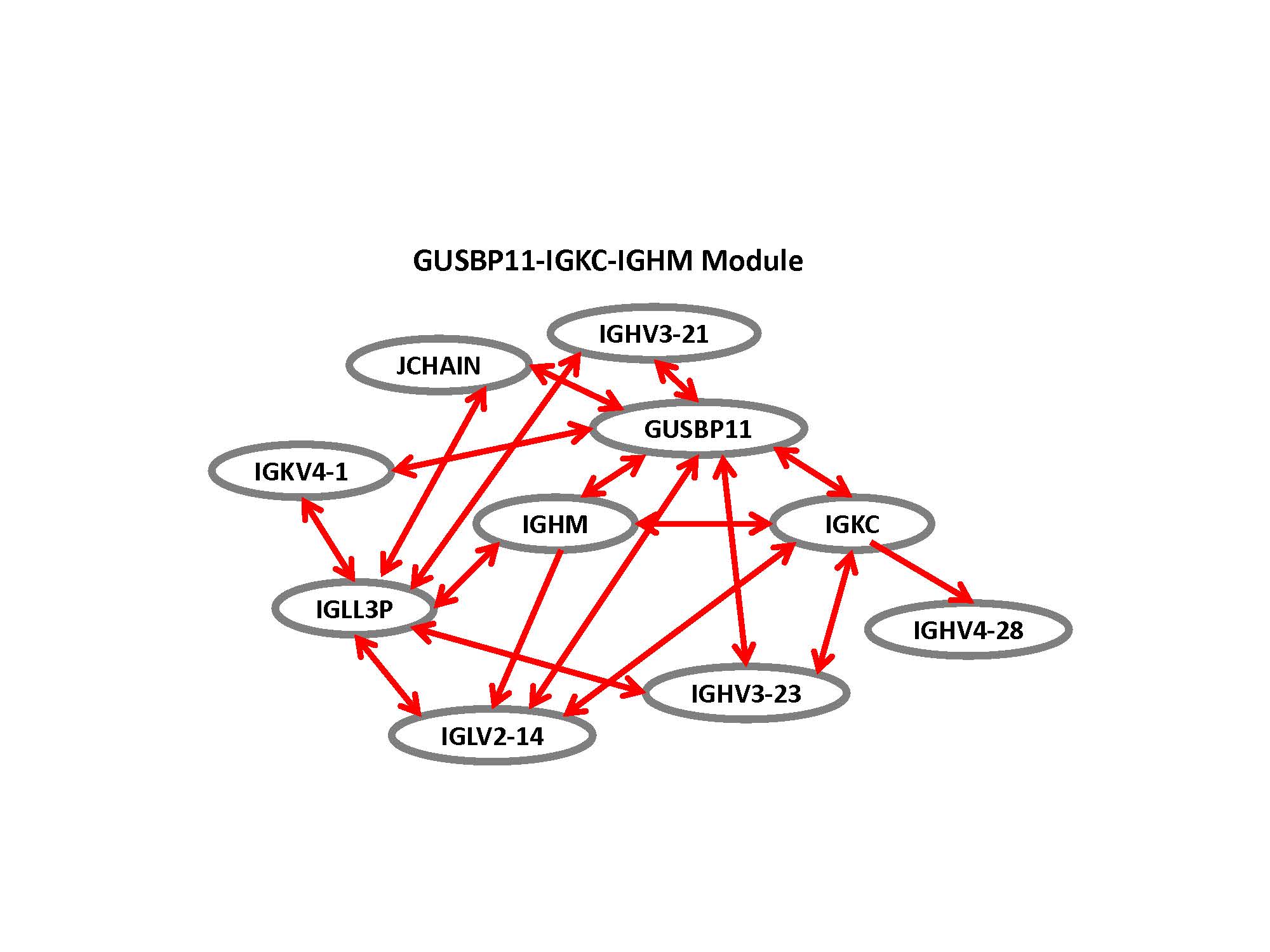

Supplement: Supplementary Figure 1 — Encoded chemosensitive (pCR) network from top 200 gene pairs of absolute association scores. Major biological processes from Gene Ontology are highlighted with their respective colors. [file DataSheet_2.zip › ANNE_Supplementary Figures S1-S7_Page_55.jpg]

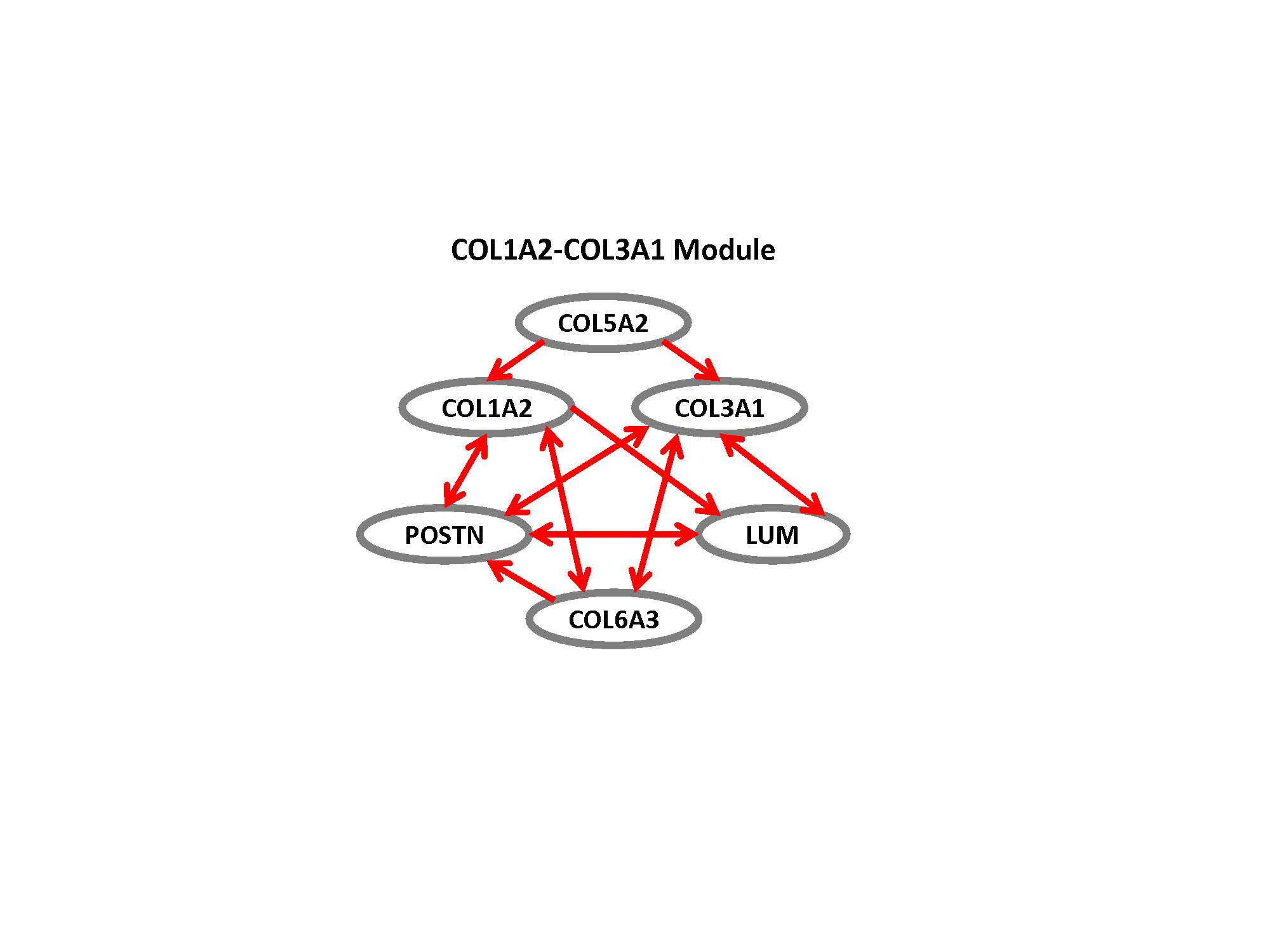

Supplement: Supplementary Figure 1 — Encoded chemosensitive (pCR) network from top 200 gene pairs of absolute association scores. Major biological processes from Gene Ontology are highlighted with their respective colors. [file DataSheet_2.zip › ANNE_Supplementary Figures S1-S7_Page_54.jpg]

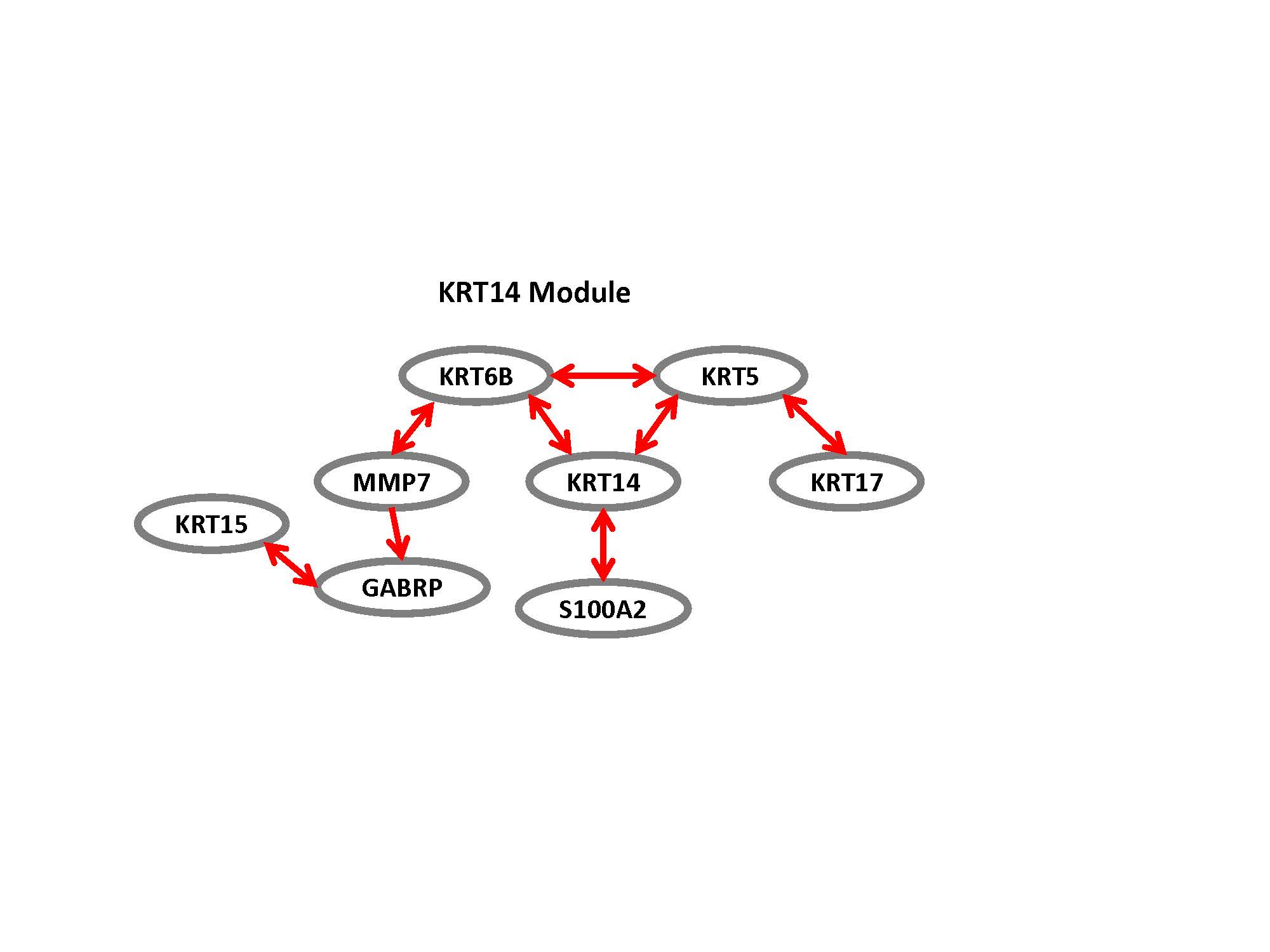

Supplement: Supplementary Figure 1 — Encoded chemosensitive (pCR) network from top 200 gene pairs of absolute association scores. Major biological processes from Gene Ontology are highlighted with their respective colors. [file DataSheet_2.zip › ANNE_Supplementary Figures S1-S7_Page_53.jpg]

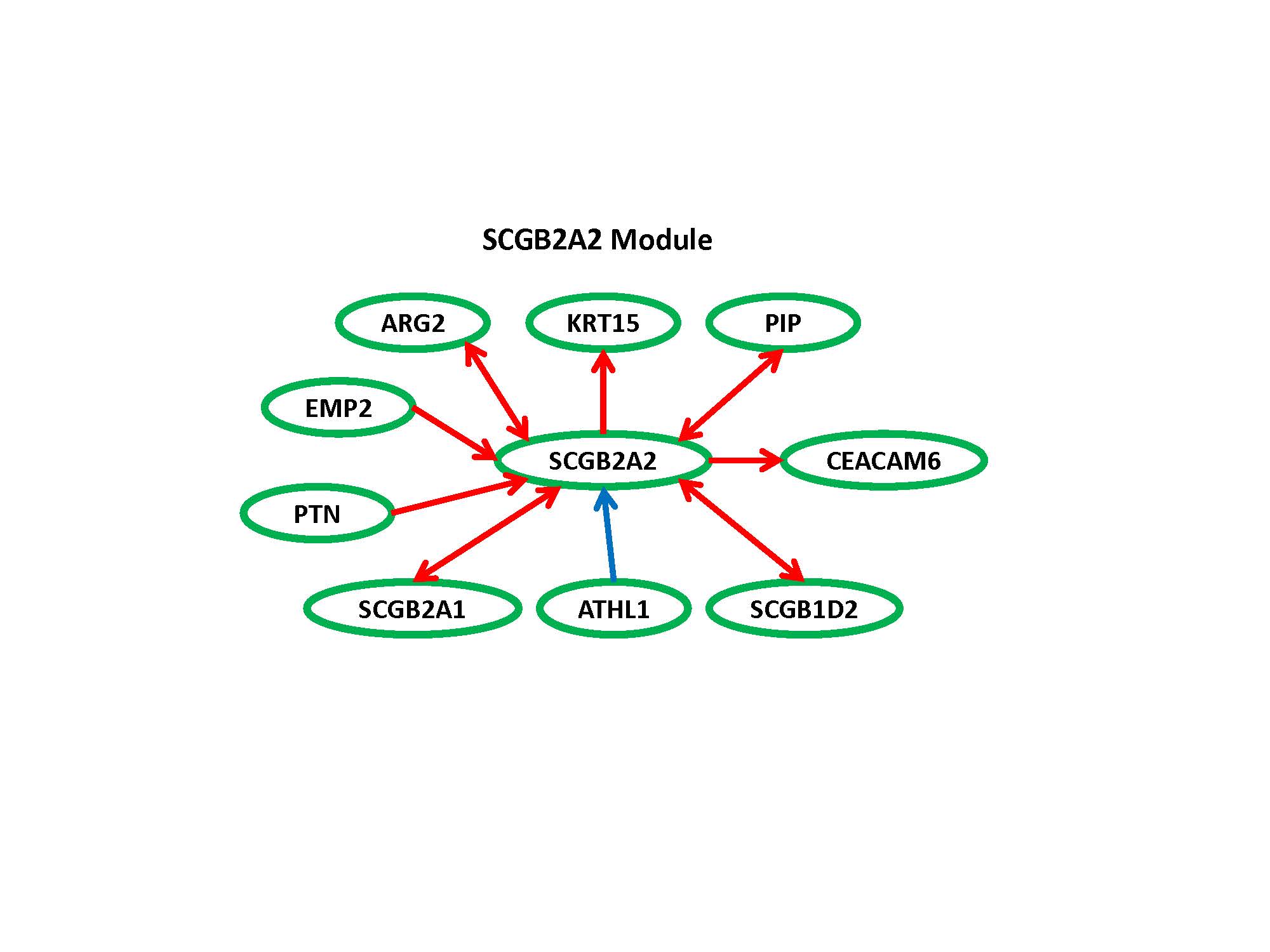

Supplement: Supplementary Figure 1 — Encoded chemosensitive (pCR) network from top 200 gene pairs of absolute association scores. Major biological processes from Gene Ontology are highlighted with their respective colors. [file DataSheet_2.zip › ANNE_Supplementary Figures S1-S7_Page_52.jpg]

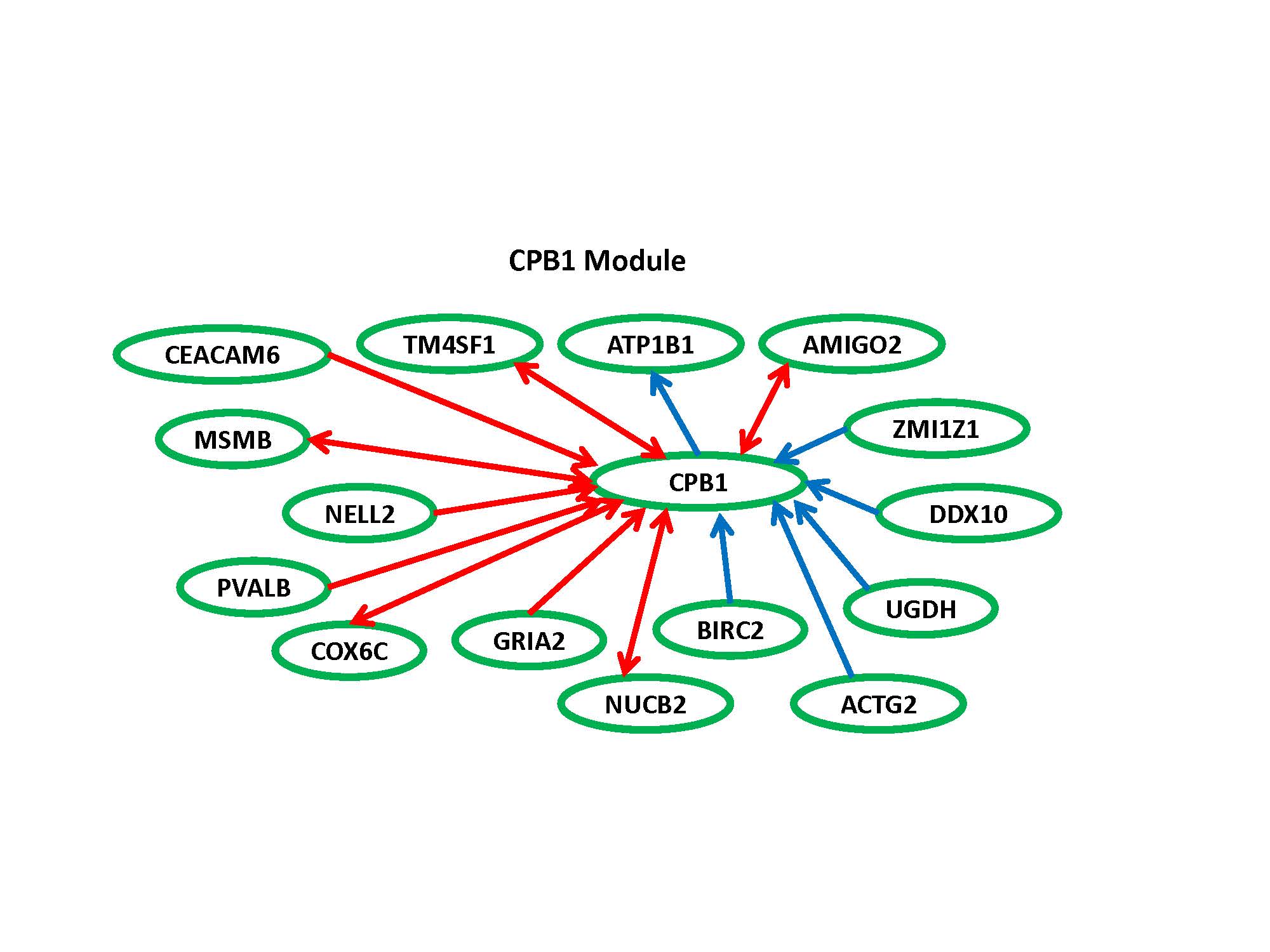

Supplement: Supplementary Figure 1 — Encoded chemosensitive (pCR) network from top 200 gene pairs of absolute association scores. Major biological processes from Gene Ontology are highlighted with their respective colors. [file DataSheet_2.zip › ANNE_Supplementary Figures S1-S7_Page_51.jpg]

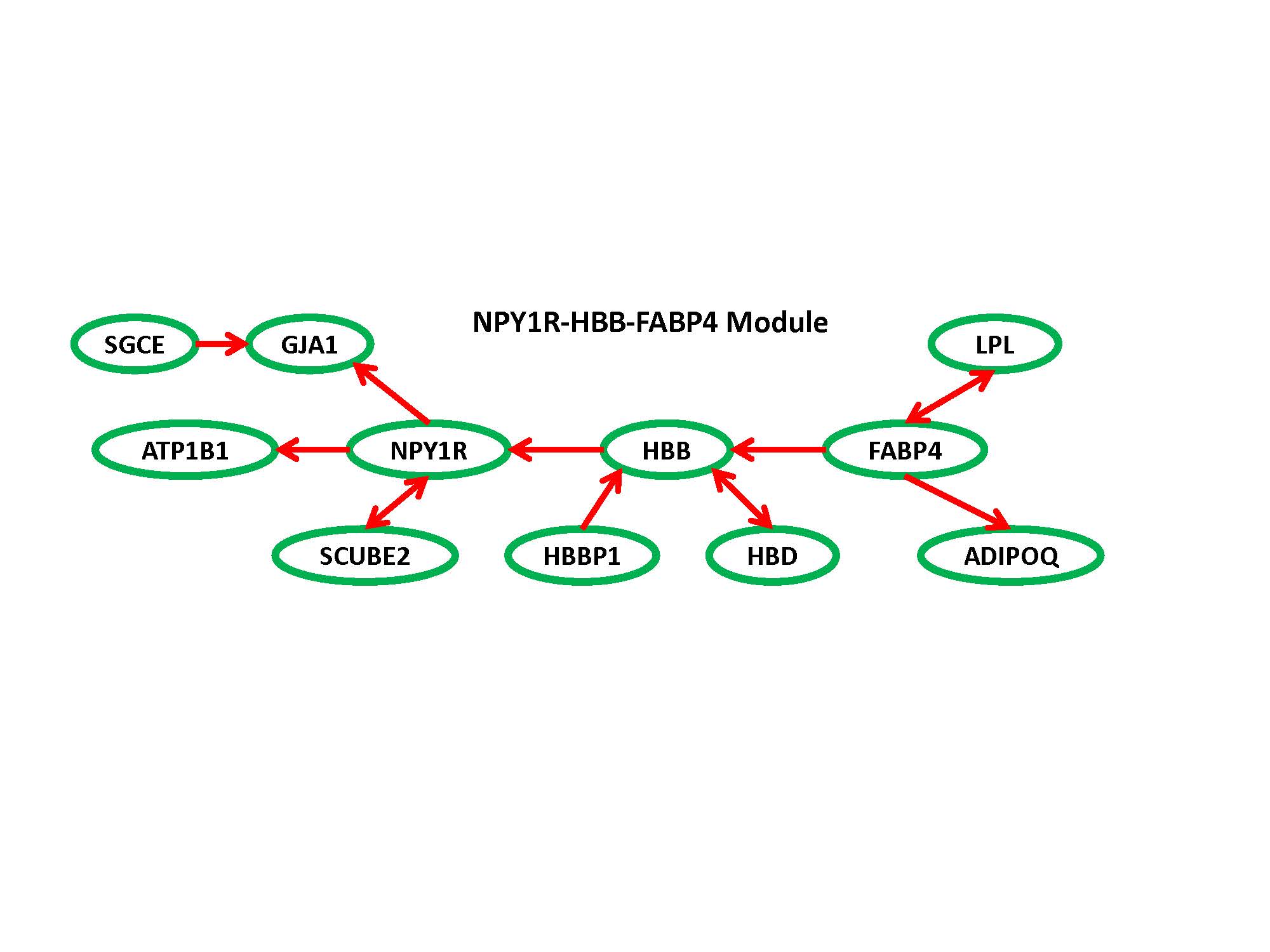

Supplement: Supplementary Figure 1 — Encoded chemosensitive (pCR) network from top 200 gene pairs of absolute association scores. Major biological processes from Gene Ontology are highlighted with their respective colors. [file DataSheet_2.zip › ANNE_Supplementary Figures S1-S7_Page_50.jpg]

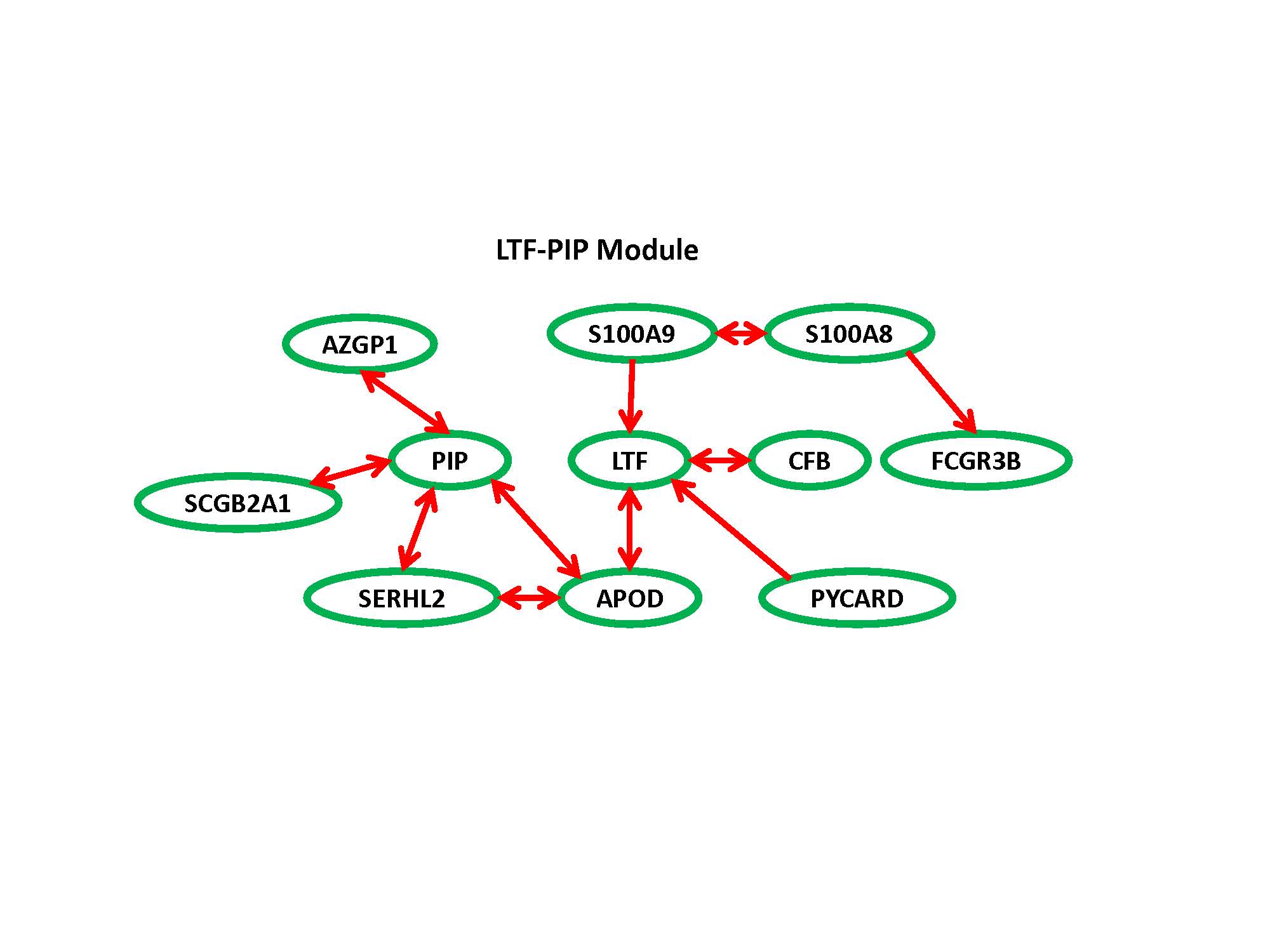

Supplement: Supplementary Figure 1 — Encoded chemosensitive (pCR) network from top 200 gene pairs of absolute association scores. Major biological processes from Gene Ontology are highlighted with their respective colors. [file DataSheet_2.zip › ANNE_Supplementary Figures S1-S7_Page_49.jpg]

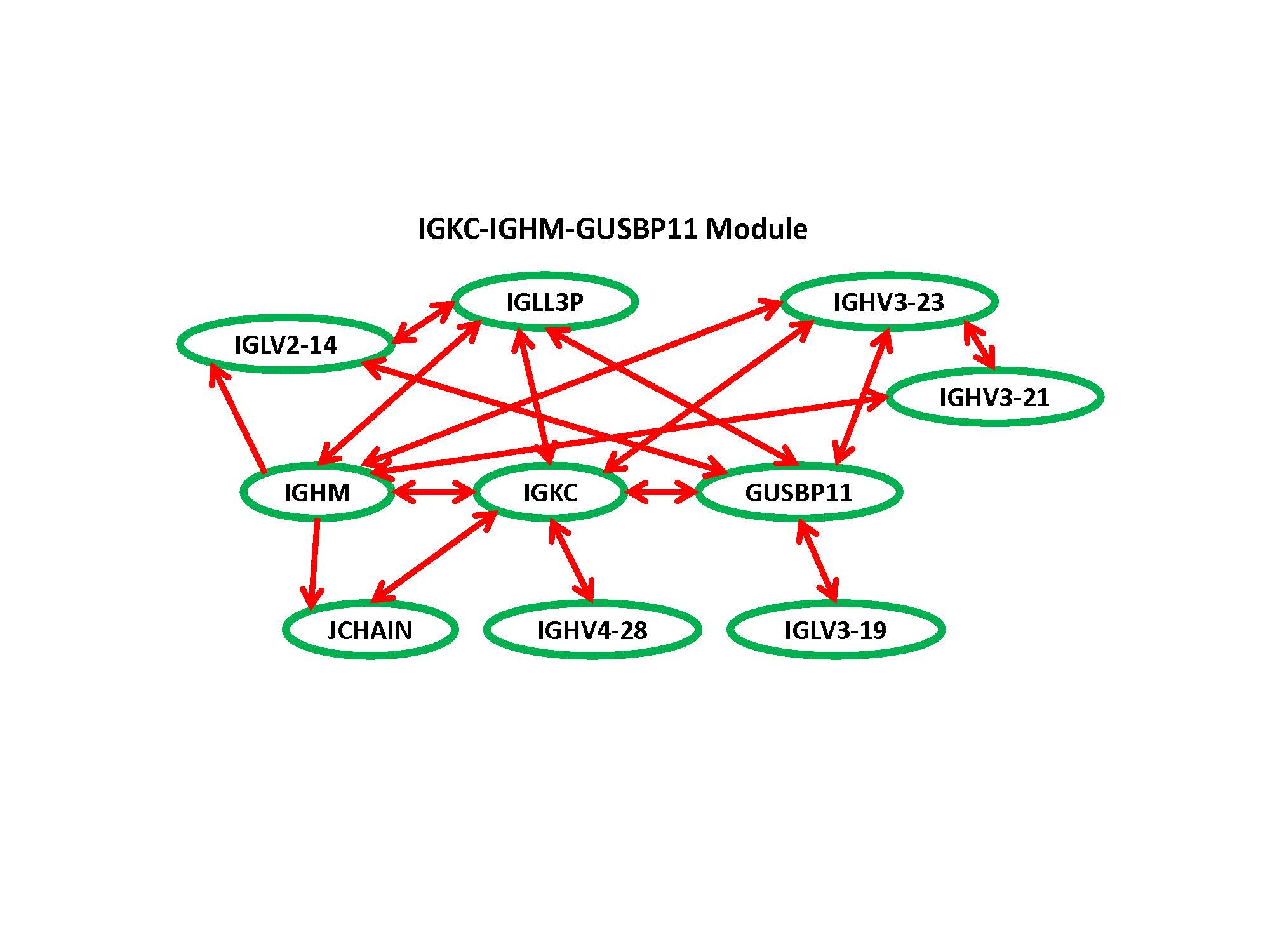

Supplement: Supplementary Figure 1 — Encoded chemosensitive (pCR) network from top 200 gene pairs of absolute association scores. Major biological processes from Gene Ontology are highlighted with their respective colors. [file DataSheet_2.zip › ANNE_Supplementary Figures S1-S7_Page_48.jpg]

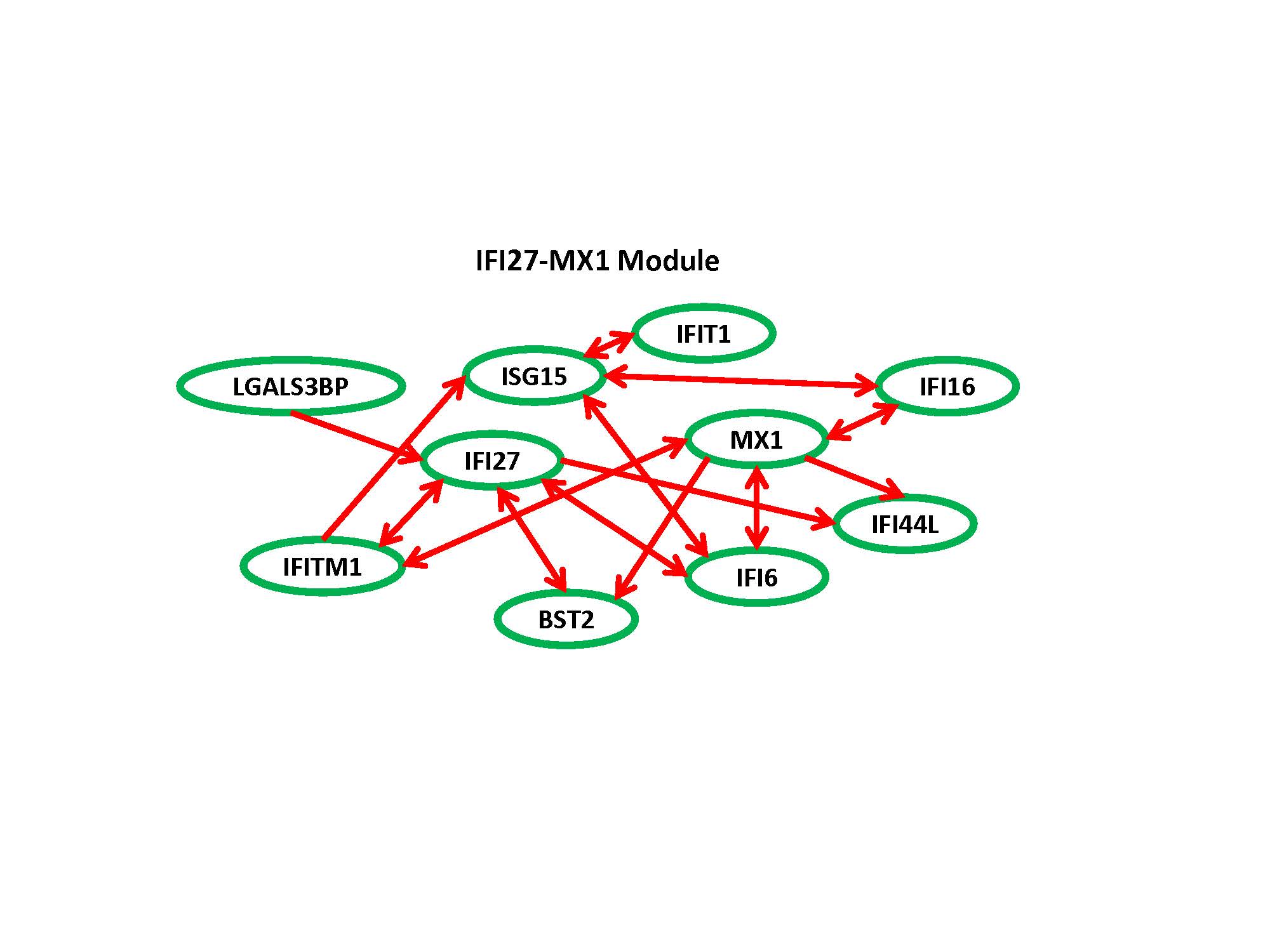

Supplement: Supplementary Figure 1 — Encoded chemosensitive (pCR) network from top 200 gene pairs of absolute association scores. Major biological processes from Gene Ontology are highlighted with their respective colors. [file DataSheet_2.zip › ANNE_Supplementary Figures S1-S7_Page_47.jpg]

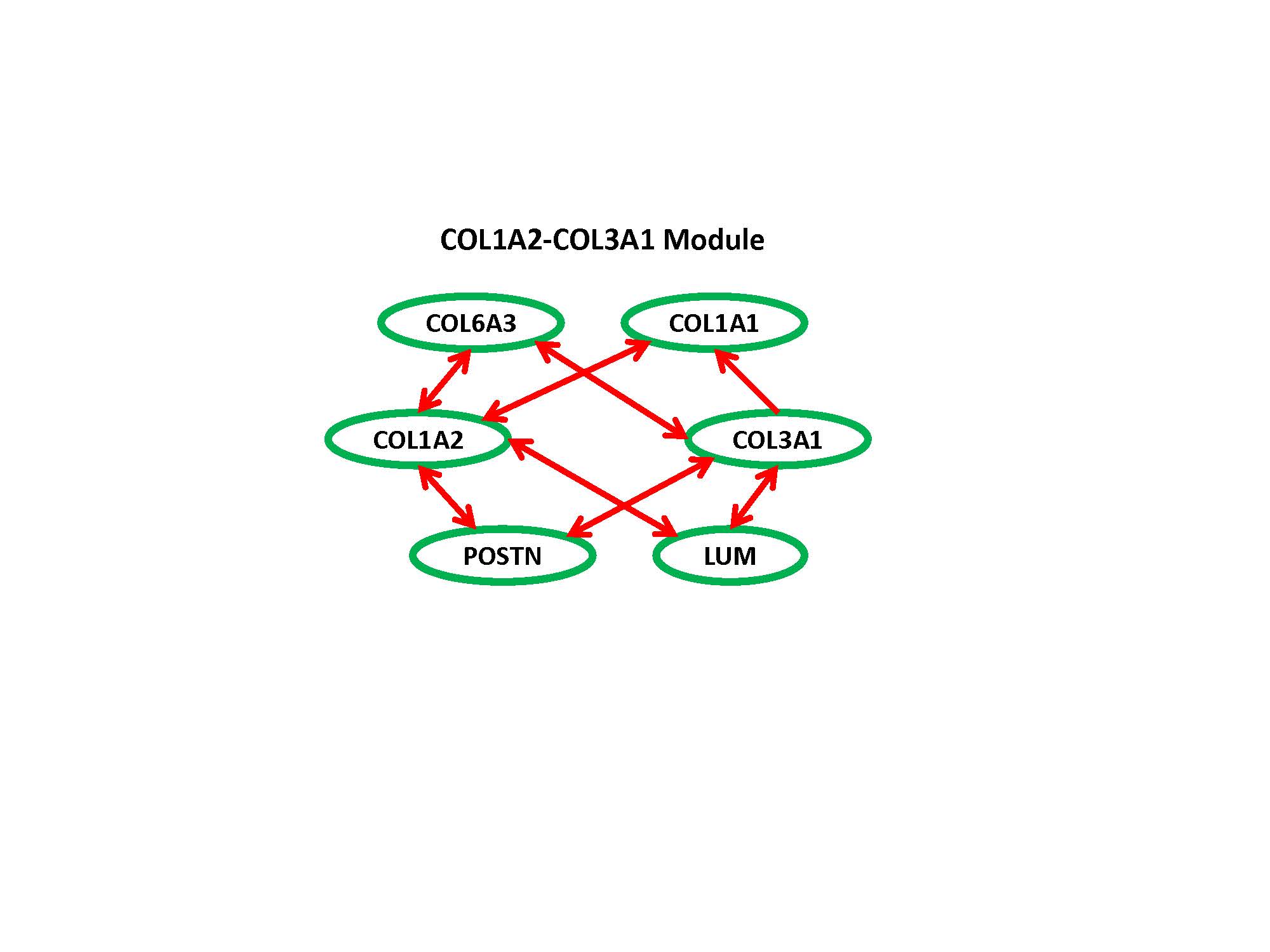

Supplement: Supplementary Figure 1 — Encoded chemosensitive (pCR) network from top 200 gene pairs of absolute association scores. Major biological processes from Gene Ontology are highlighted with their respective colors. [file DataSheet_2.zip › ANNE_Supplementary Figures S1-S7_Page_46.jpg]

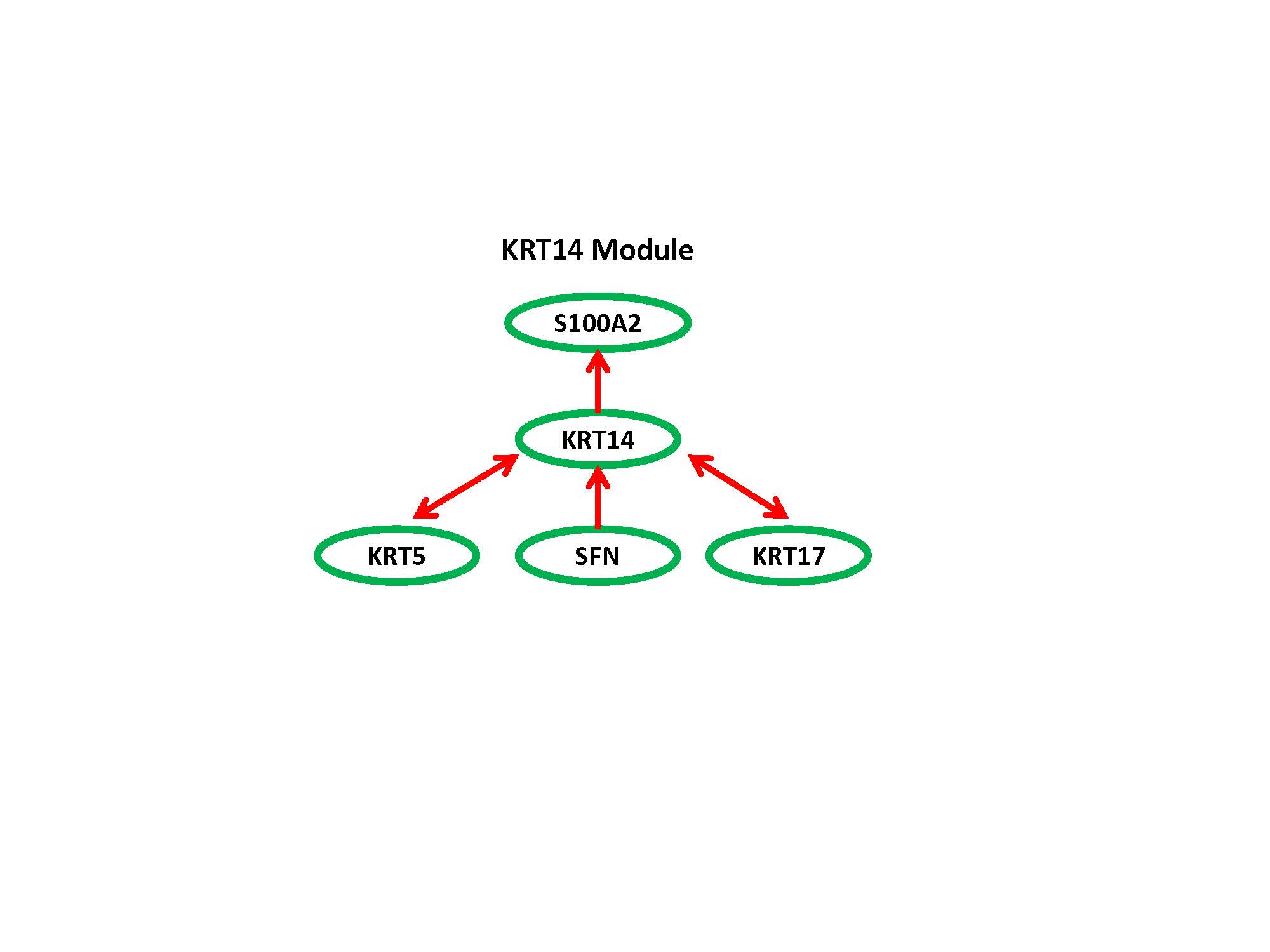

Supplement: Supplementary Figure 1 — Encoded chemosensitive (pCR) network from top 200 gene pairs of absolute association scores. Major biological processes from Gene Ontology are highlighted with their respective colors. [file DataSheet_2.zip › ANNE_Supplementary Figures S1-S7_Page_45.jpg]

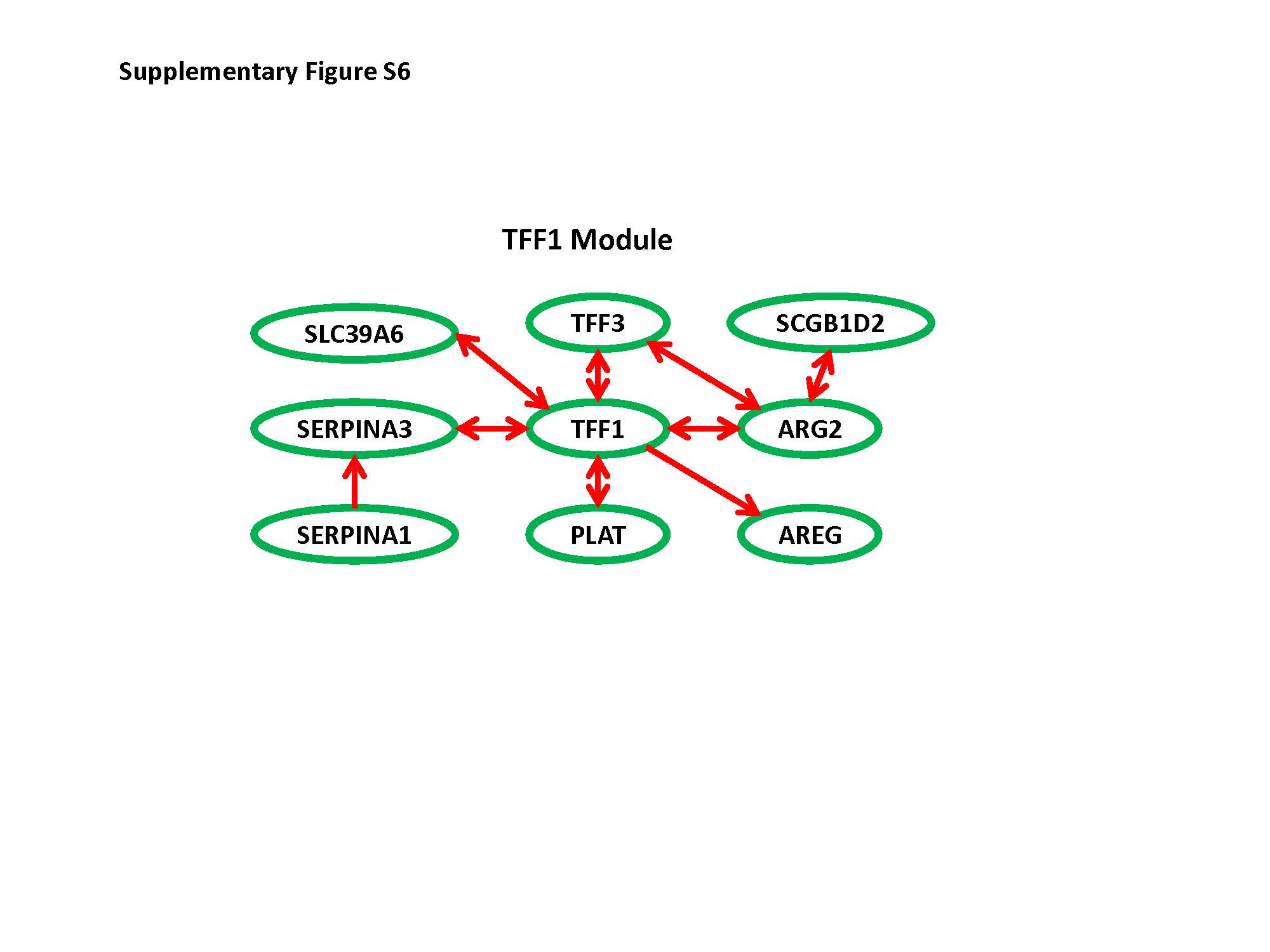

Supplement: Supplementary Figure 1 — Encoded chemosensitive (pCR) network from top 200 gene pairs of absolute association scores. Major biological processes from Gene Ontology are highlighted with their respective colors. [file DataSheet_2.zip › ANNE_Supplementary Figures S1-S7_Page_44.jpg]

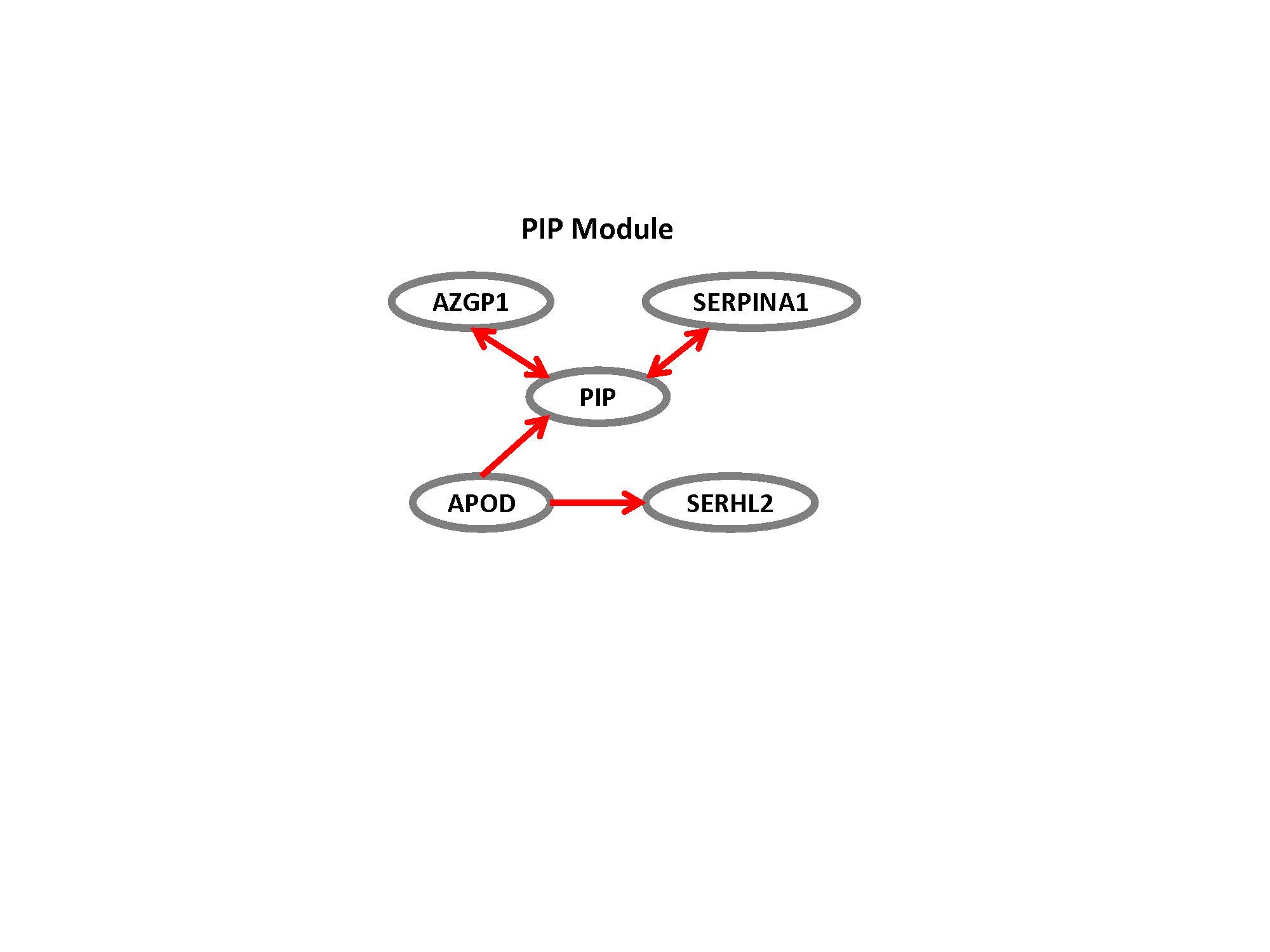

Supplement: Supplementary Figure 1 — Encoded chemosensitive (pCR) network from top 200 gene pairs of absolute association scores. Major biological processes from Gene Ontology are highlighted with their respective colors. [file DataSheet_2.zip › ANNE_Supplementary Figures S1-S7_Page_43.jpg]

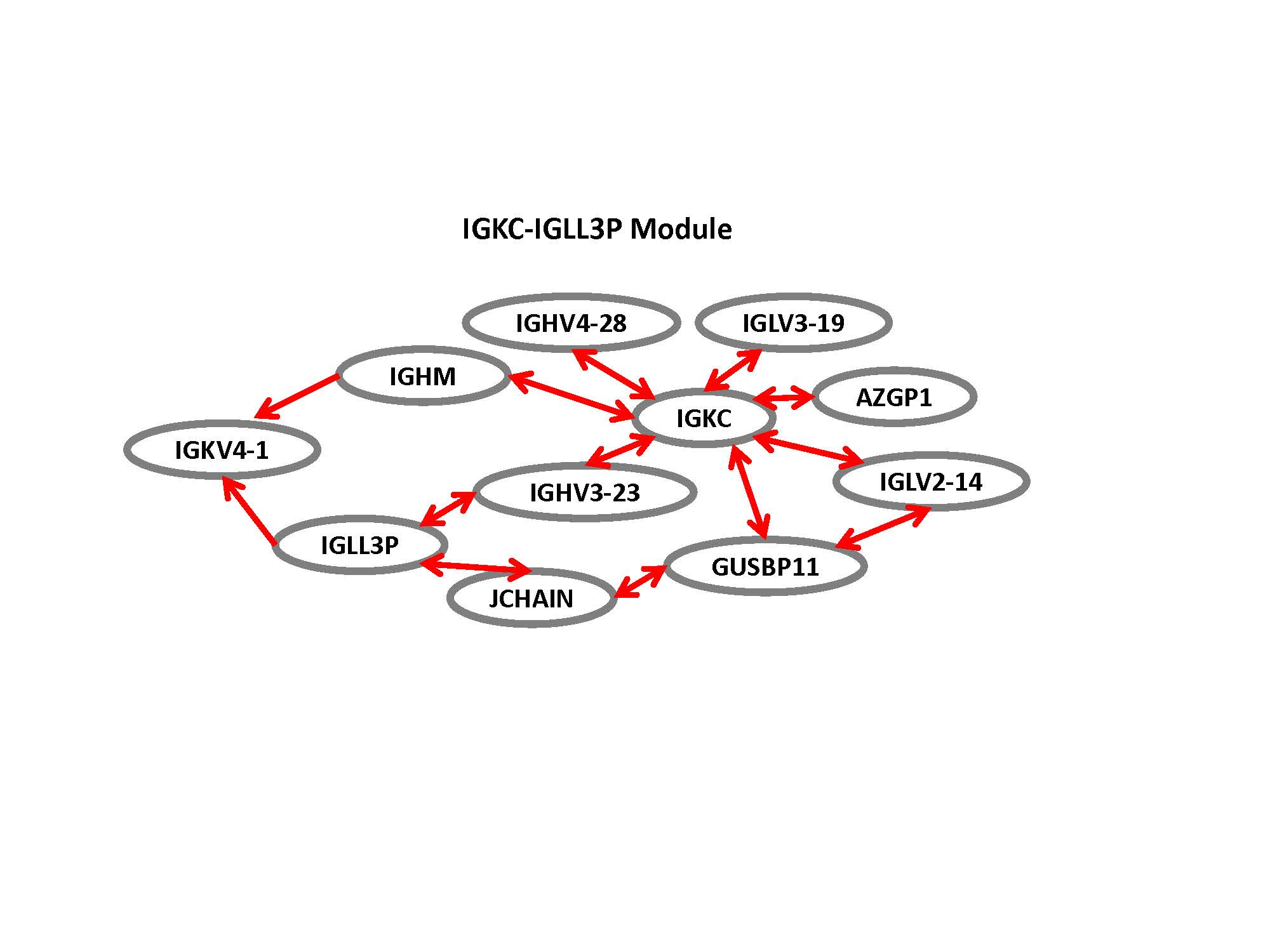

Supplement: Supplementary Figure 1 — Encoded chemosensitive (pCR) network from top 200 gene pairs of absolute association scores. Major biological processes from Gene Ontology are highlighted with their respective colors. [file DataSheet_2.zip › ANNE_Supplementary Figures S1-S7_Page_42.jpg]

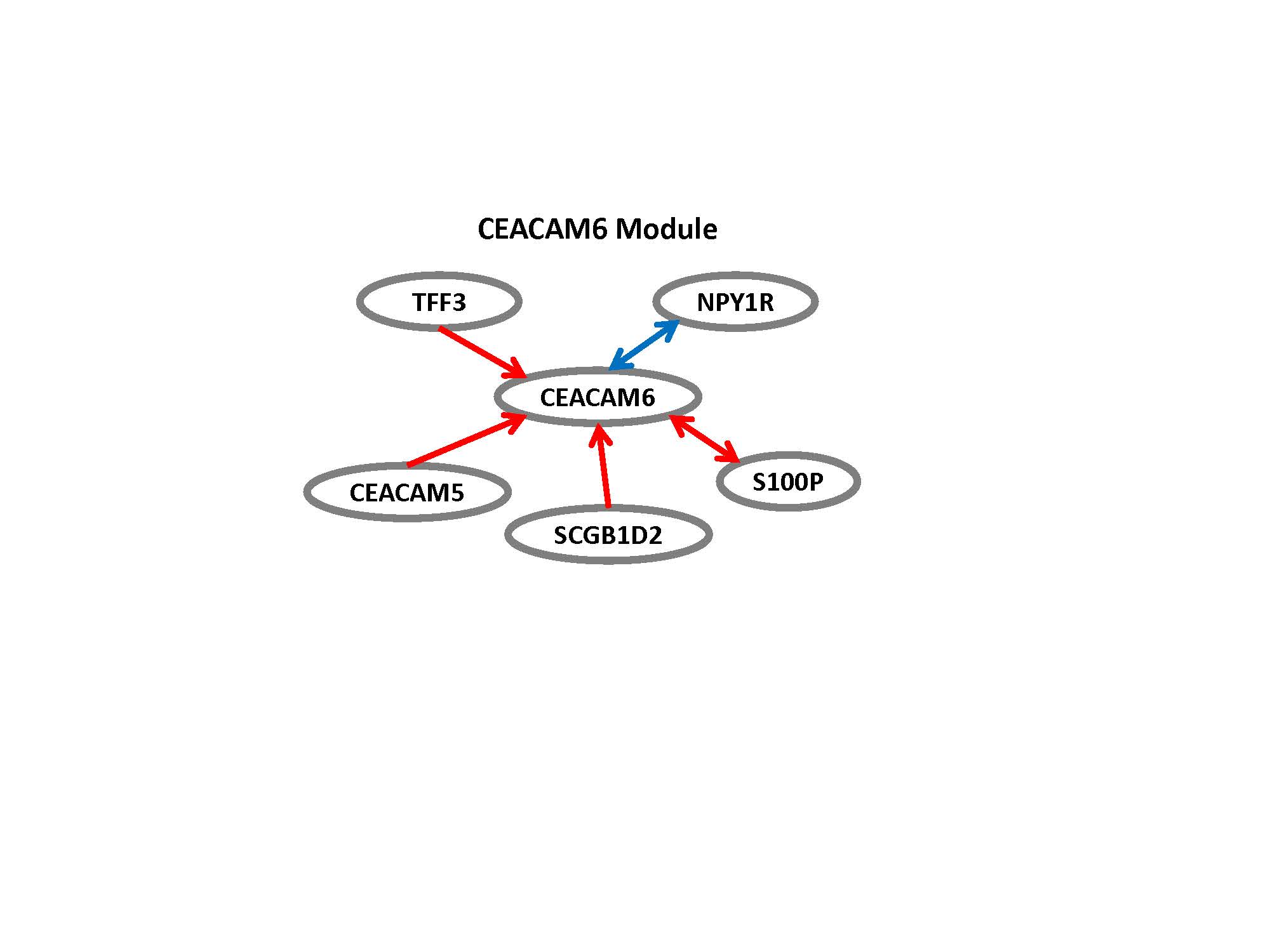

Supplement: Supplementary Figure 1 — Encoded chemosensitive (pCR) network from top 200 gene pairs of absolute association scores. Major biological processes from Gene Ontology are highlighted with their respective colors. [file DataSheet_2.zip › ANNE_Supplementary Figures S1-S7_Page_41.jpg]

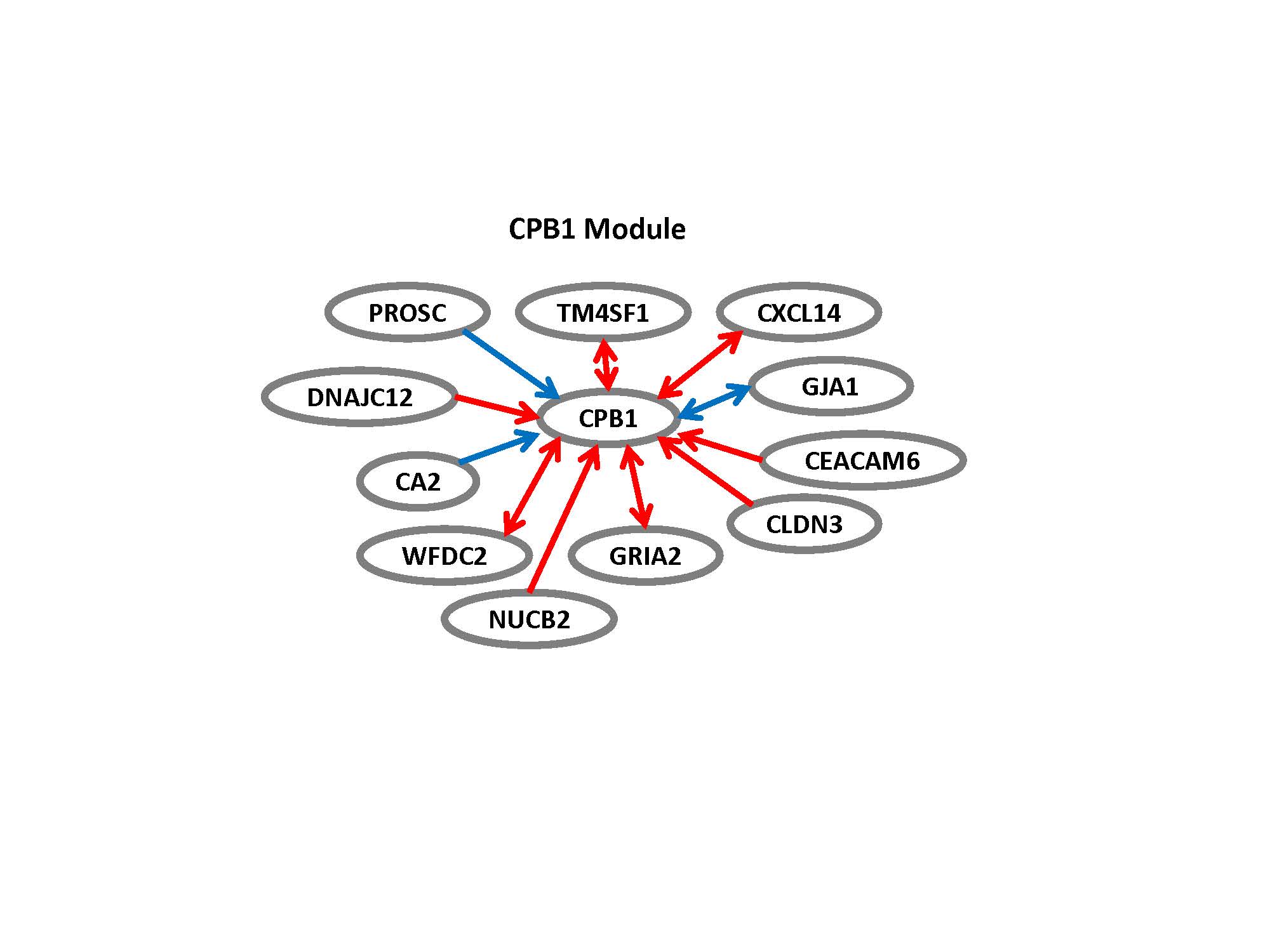

Supplement: Supplementary Figure 1 — Encoded chemosensitive (pCR) network from top 200 gene pairs of absolute association scores. Major biological processes from Gene Ontology are highlighted with their respective colors. [file DataSheet_2.zip › ANNE_Supplementary Figures S1-S7_Page_40.jpg]

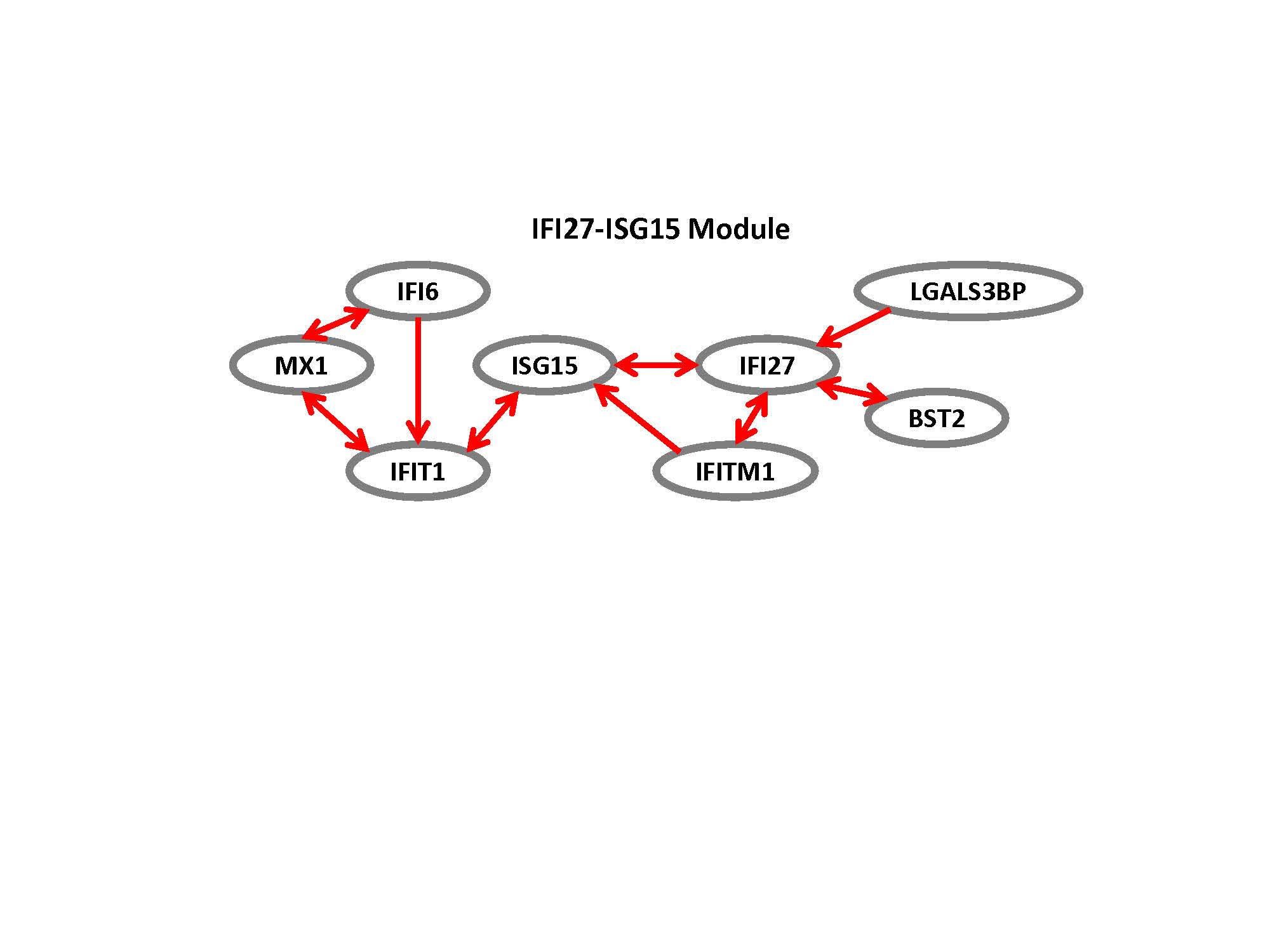

Supplement: Supplementary Figure 1 — Encoded chemosensitive (pCR) network from top 200 gene pairs of absolute association scores. Major biological processes from Gene Ontology are highlighted with their respective colors. [file DataSheet_2.zip › ANNE_Supplementary Figures S1-S7_Page_39.jpg]

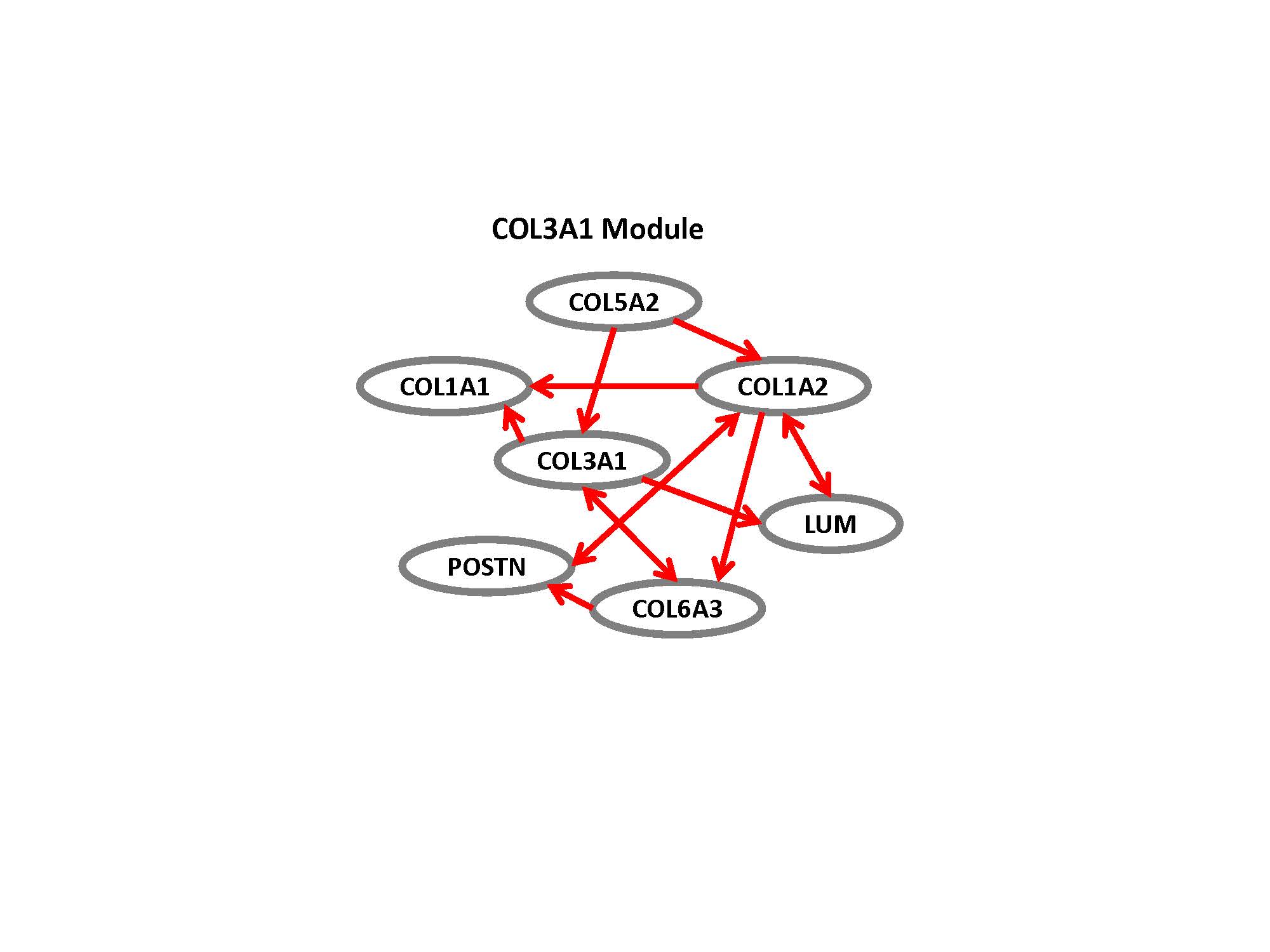

Supplement: Supplementary Figure 1 — Encoded chemosensitive (pCR) network from top 200 gene pairs of absolute association scores. Major biological processes from Gene Ontology are highlighted with their respective colors. [file DataSheet_2.zip › ANNE_Supplementary Figures S1-S7_Page_38.jpg]

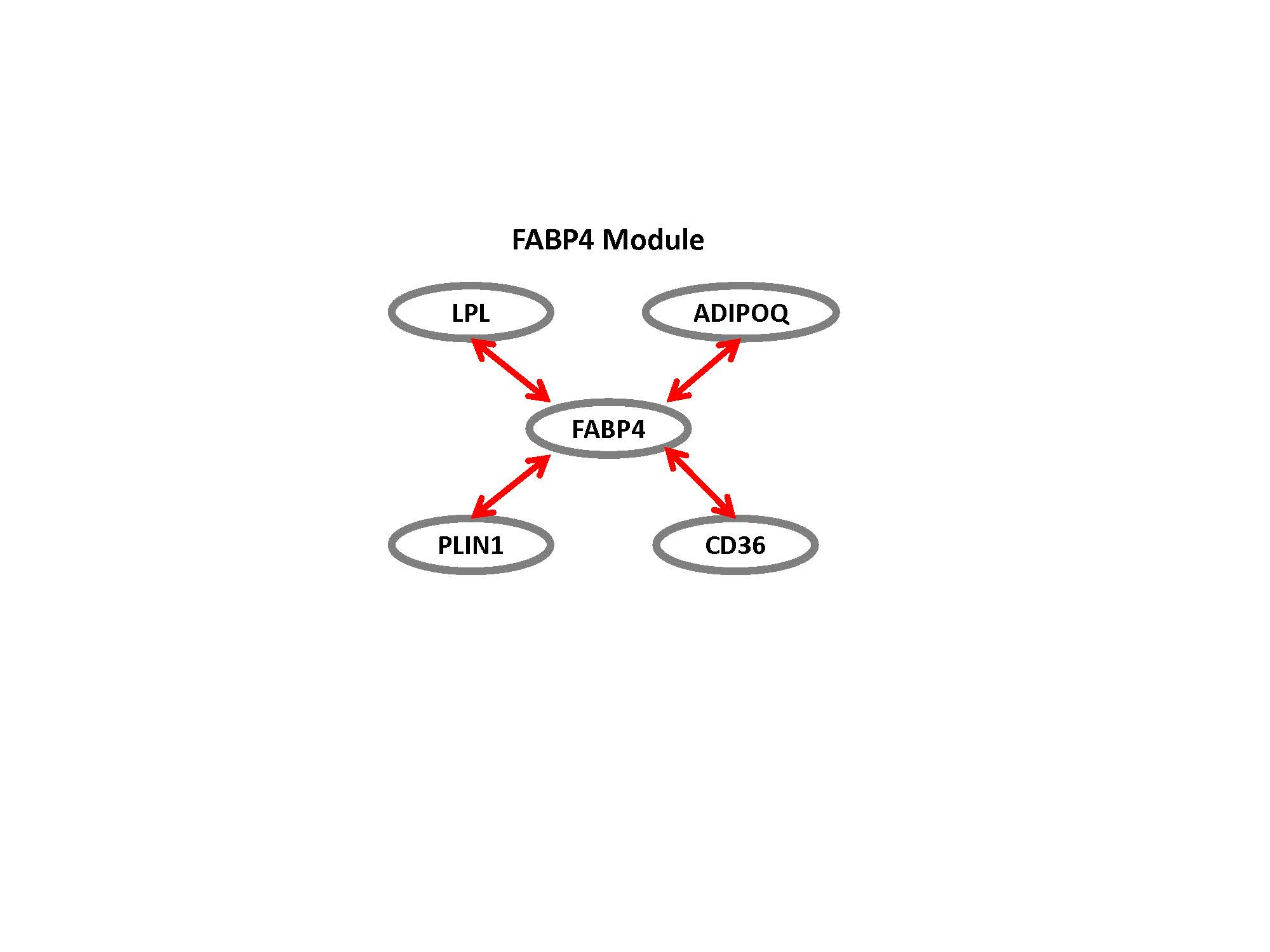

Supplement: Supplementary Figure 1 — Encoded chemosensitive (pCR) network from top 200 gene pairs of absolute association scores. Major biological processes from Gene Ontology are highlighted with their respective colors. [file DataSheet_2.zip › ANNE_Supplementary Figures S1-S7_Page_37.jpg]

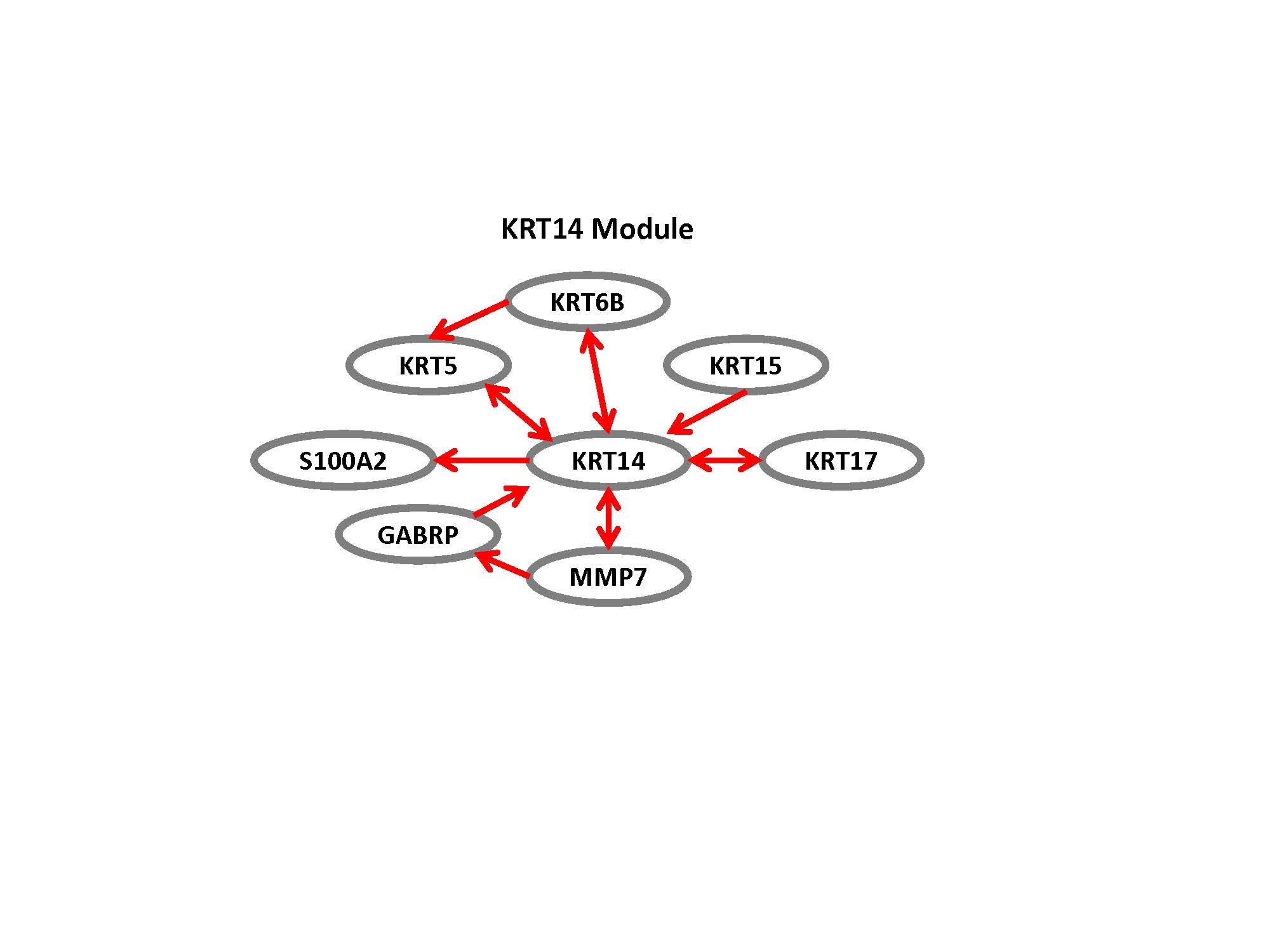

Supplement: Supplementary Figure 1 — Encoded chemosensitive (pCR) network from top 200 gene pairs of absolute association scores. Major biological processes from Gene Ontology are highlighted with their respective colors. [file DataSheet_2.zip › ANNE_Supplementary Figures S1-S7_Page_36.jpg]

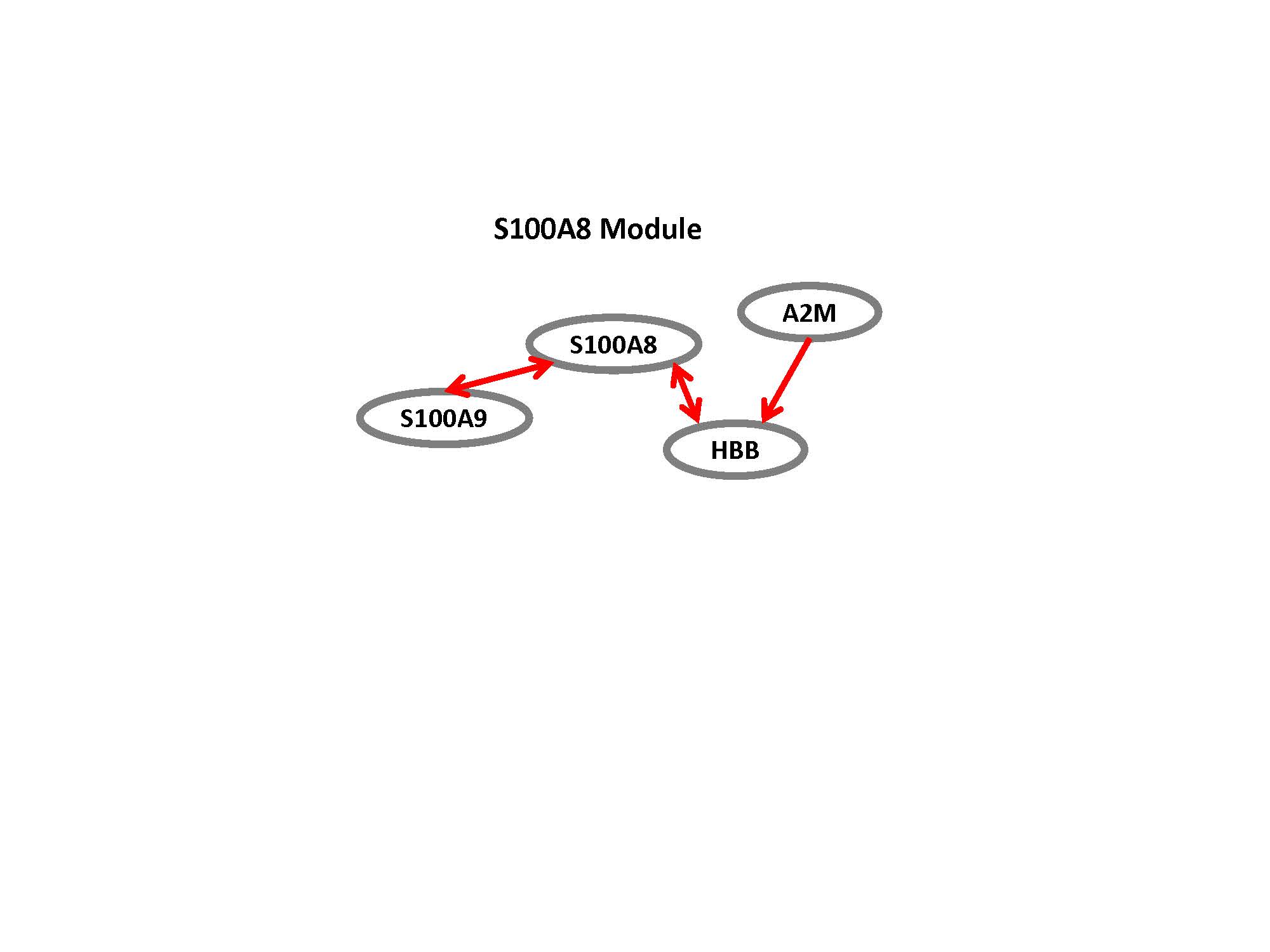

Supplement: Supplementary Figure 1 — Encoded chemosensitive (pCR) network from top 200 gene pairs of absolute association scores. Major biological processes from Gene Ontology are highlighted with their respective colors. [file DataSheet_2.zip › ANNE_Supplementary Figures S1-S7_Page_35.jpg]

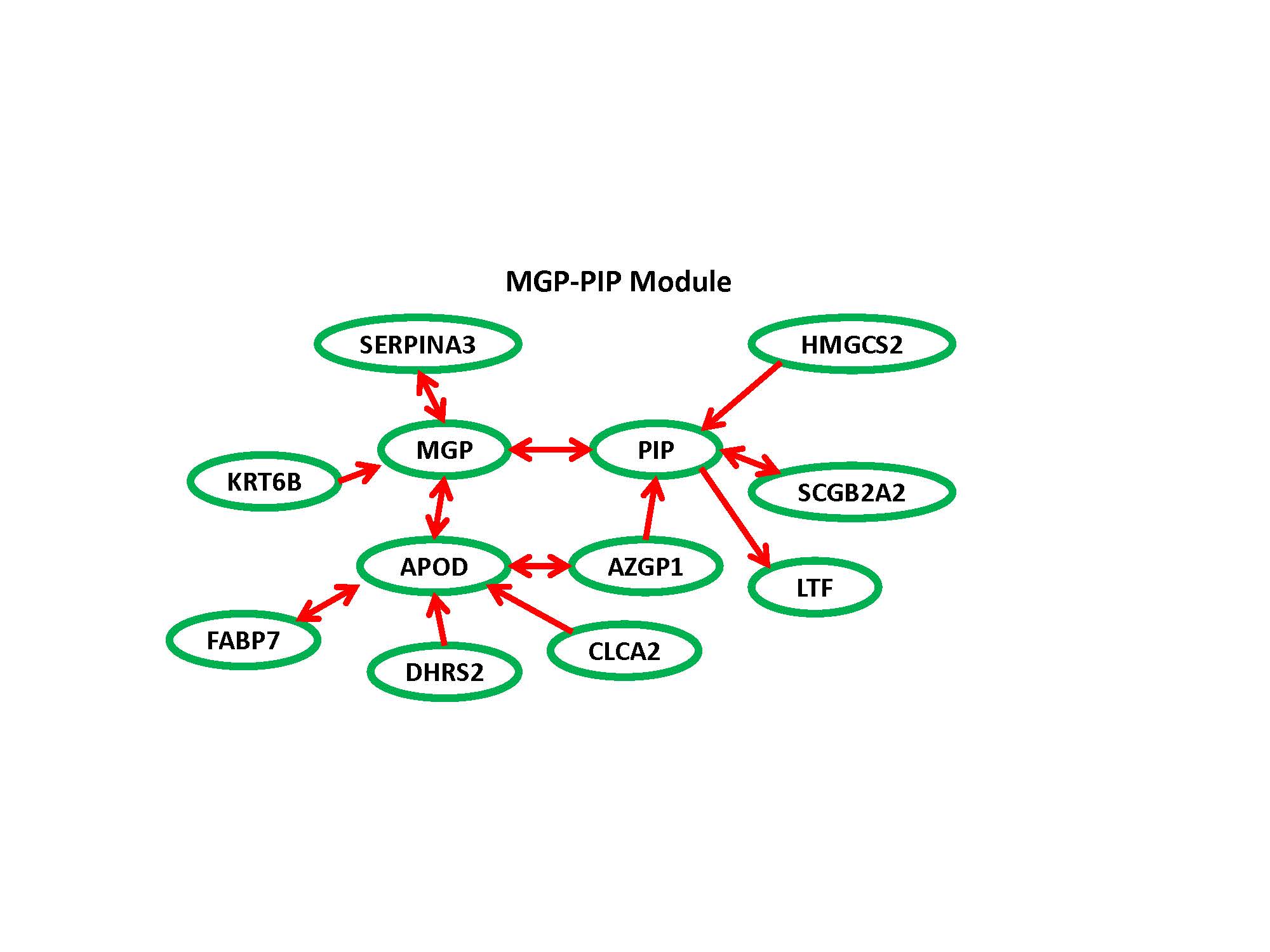

Supplement: Supplementary Figure 1 — Encoded chemosensitive (pCR) network from top 200 gene pairs of absolute association scores. Major biological processes from Gene Ontology are highlighted with their respective colors. [file DataSheet_2.zip › ANNE_Supplementary Figures S1-S7_Page_34.jpg]

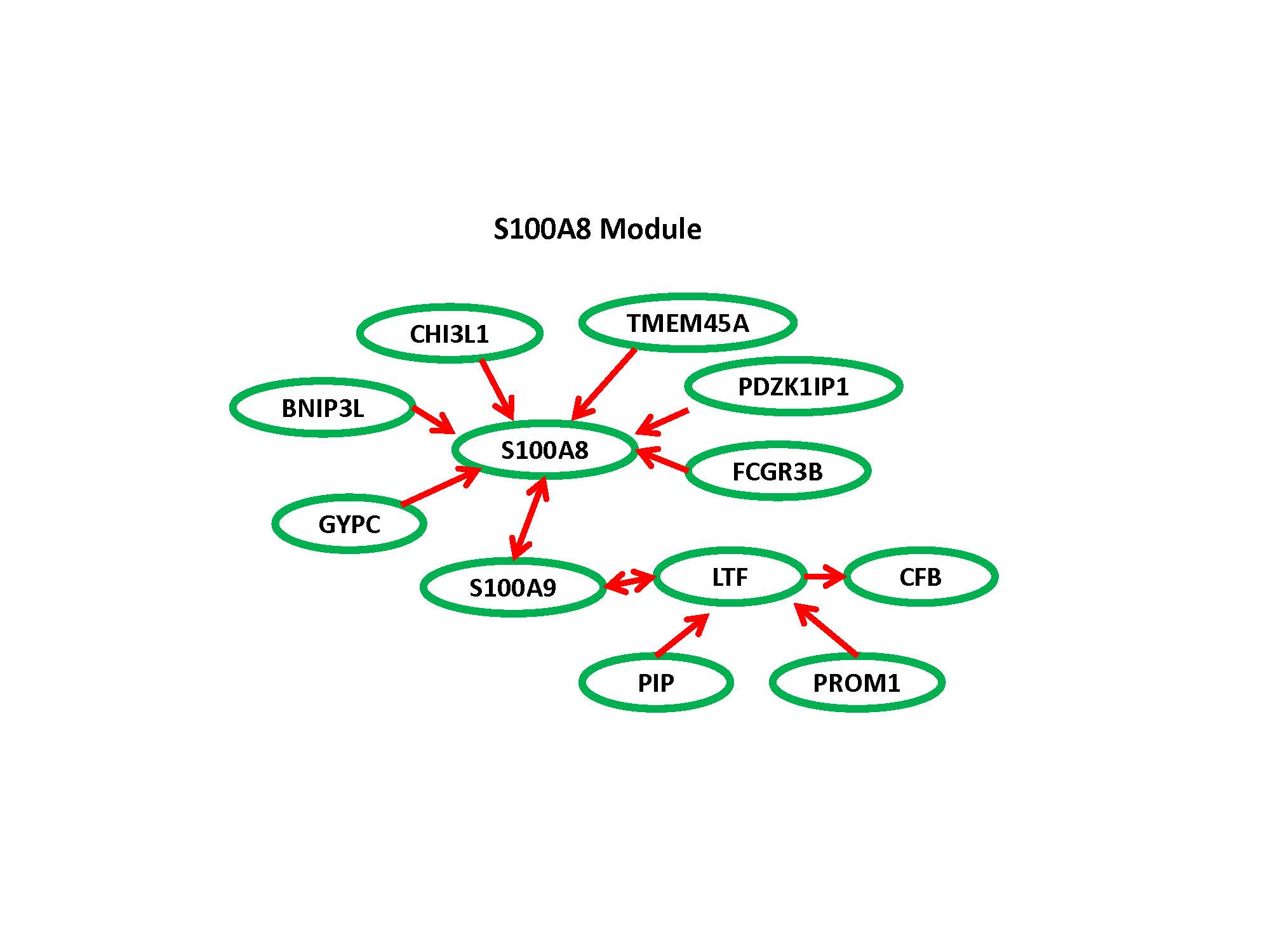

Supplement: Supplementary Figure 1 — Encoded chemosensitive (pCR) network from top 200 gene pairs of absolute association scores. Major biological processes from Gene Ontology are highlighted with their respective colors. [file DataSheet_2.zip › ANNE_Supplementary Figures S1-S7_Page_33.jpg]

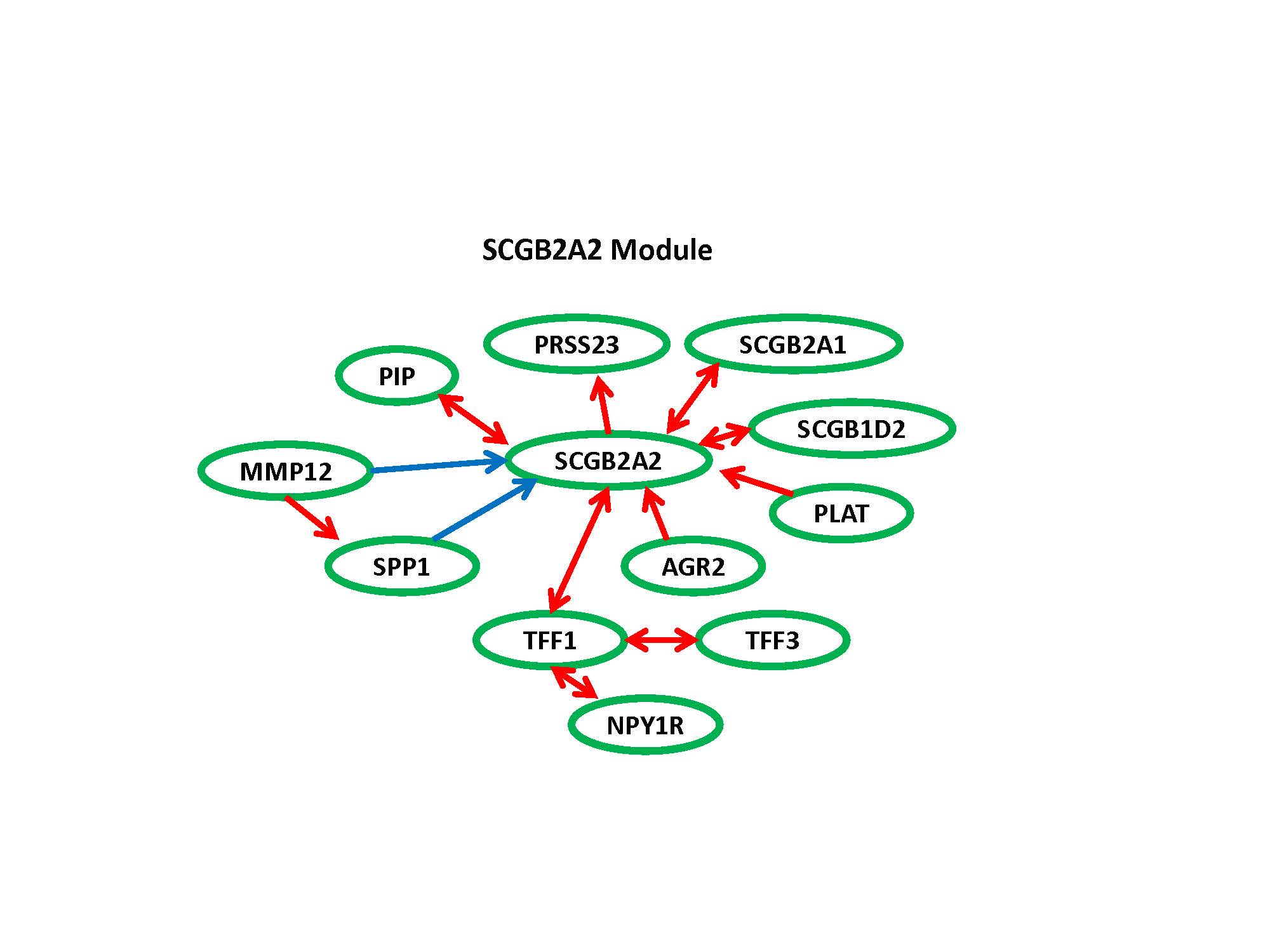

Supplement: Supplementary Figure 1 — Encoded chemosensitive (pCR) network from top 200 gene pairs of absolute association scores. Major biological processes from Gene Ontology are highlighted with their respective colors. [file DataSheet_2.zip › ANNE_Supplementary Figures S1-S7_Page_32.jpg]

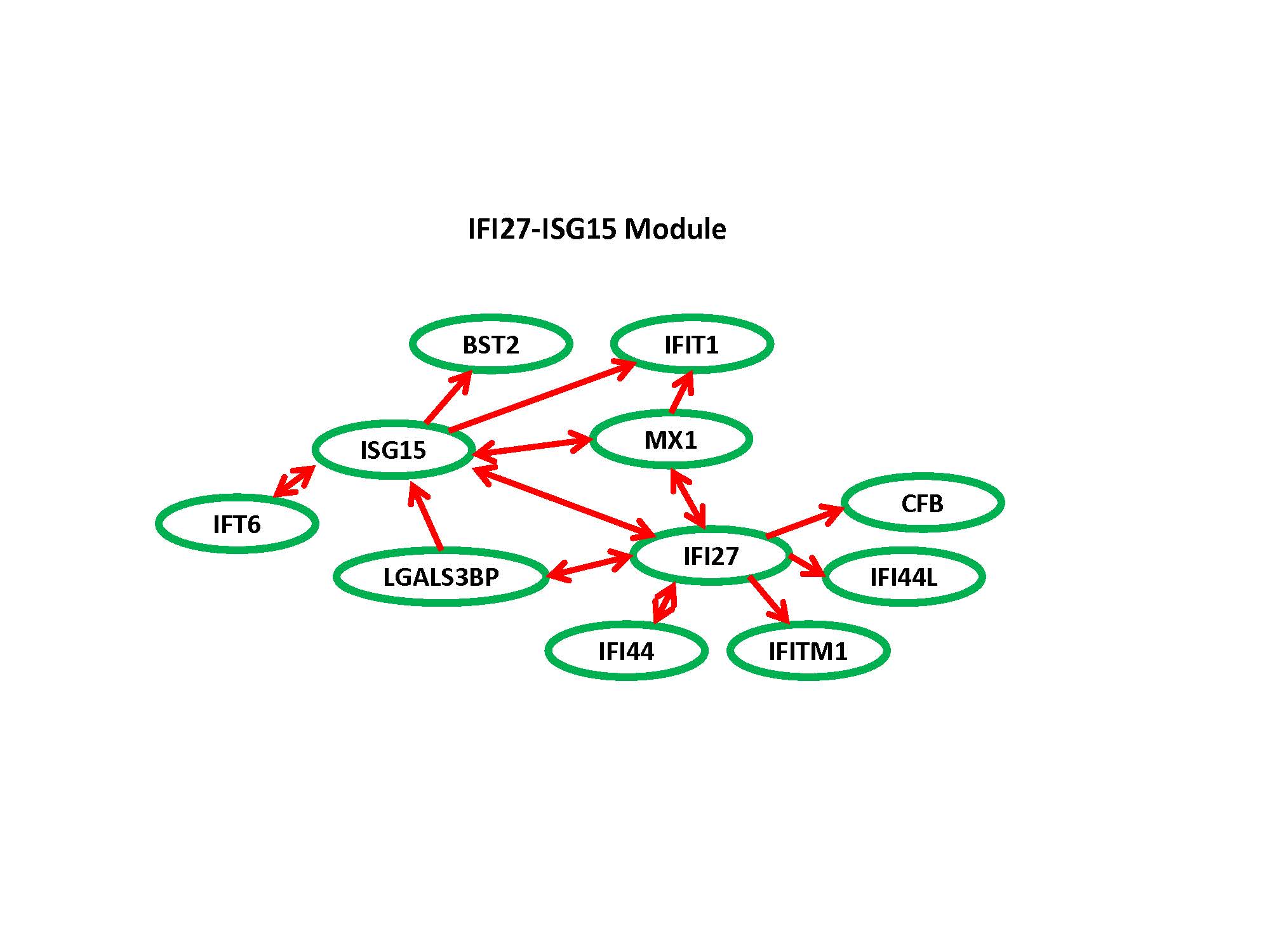

Supplement: Supplementary Figure 1 — Encoded chemosensitive (pCR) network from top 200 gene pairs of absolute association scores. Major biological processes from Gene Ontology are highlighted with their respective colors. [file DataSheet_2.zip › ANNE_Supplementary Figures S1-S7_Page_31.jpg]

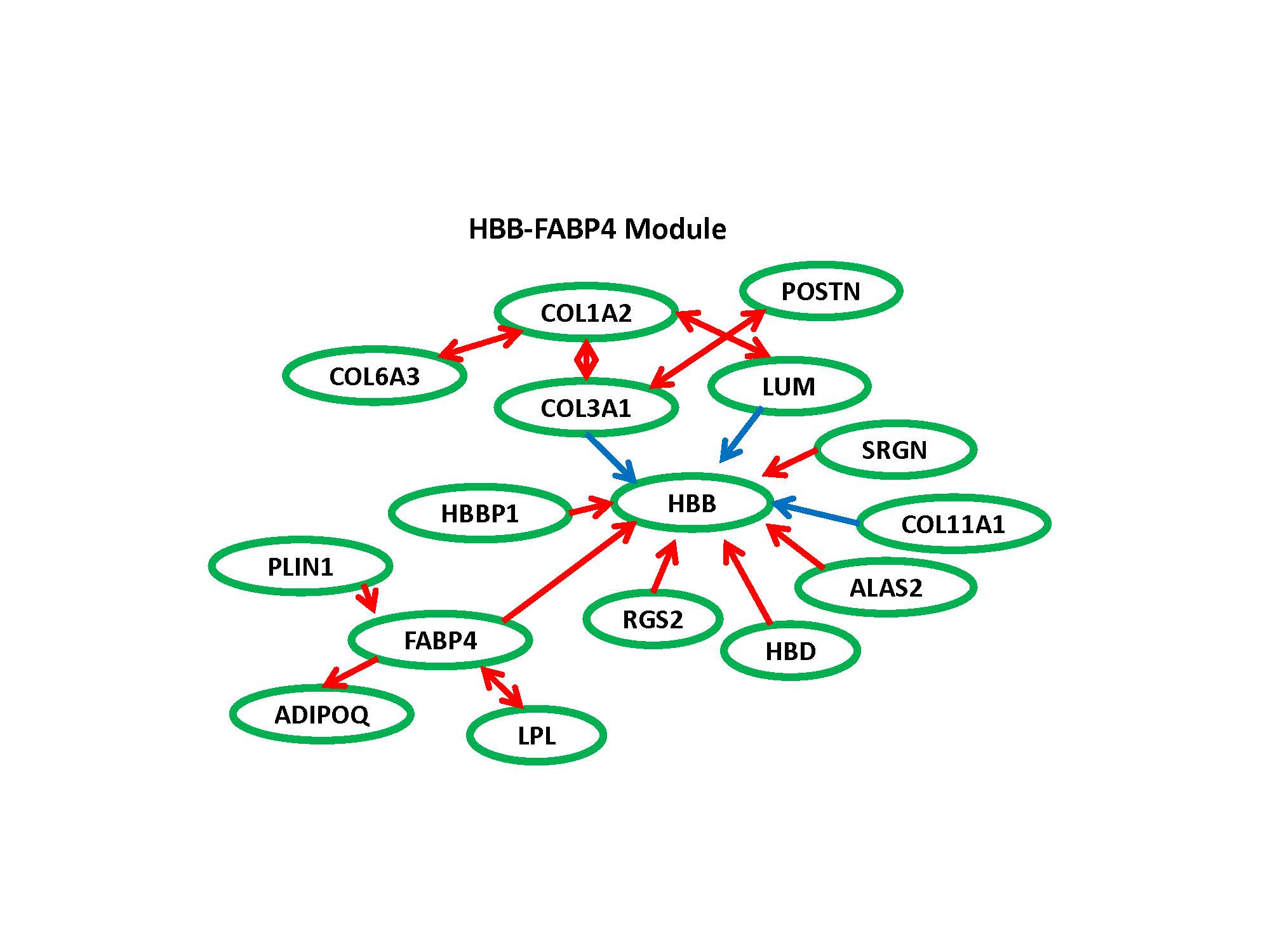

Supplement: Supplementary Figure 1 — Encoded chemosensitive (pCR) network from top 200 gene pairs of absolute association scores. Major biological processes from Gene Ontology are highlighted with their respective colors. [file DataSheet_2.zip › ANNE_Supplementary Figures S1-S7_Page_30.jpg]

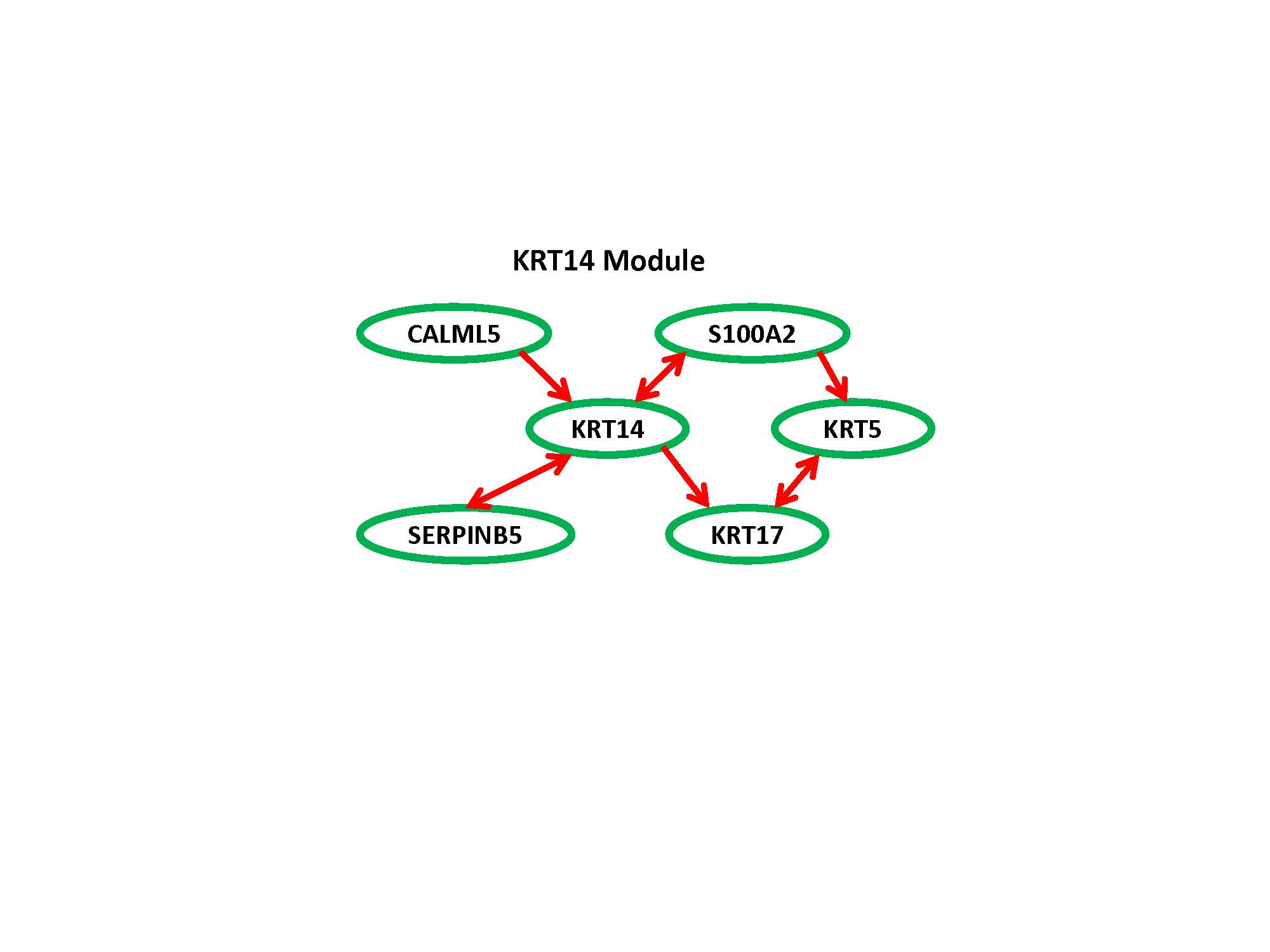

Supplement: Supplementary Figure 1 — Encoded chemosensitive (pCR) network from top 200 gene pairs of absolute association scores. Major biological processes from Gene Ontology are highlighted with their respective colors. [file DataSheet_2.zip › ANNE_Supplementary Figures S1-S7_Page_29.jpg]

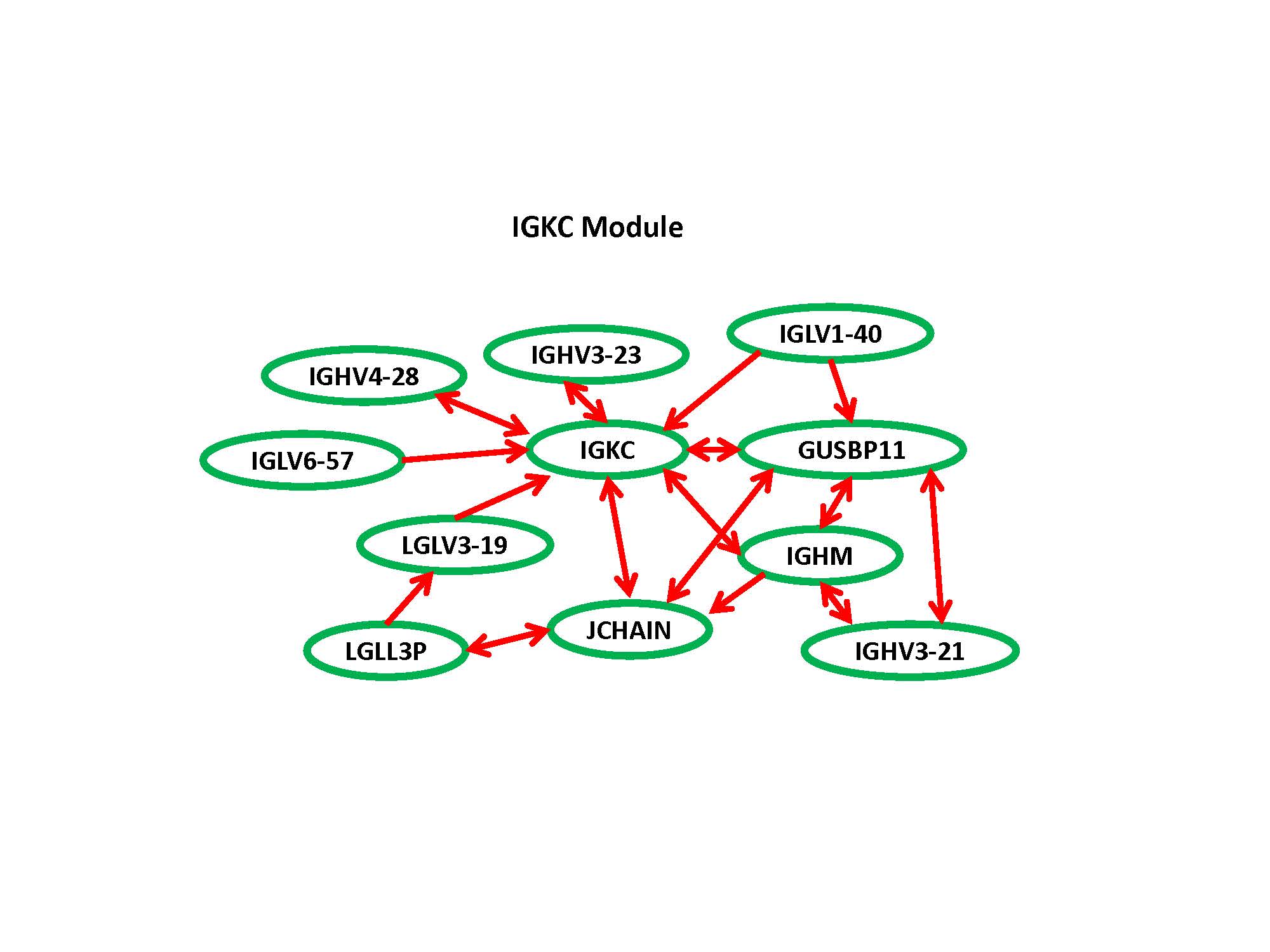

Supplement: Supplementary Figure 1 — Encoded chemosensitive (pCR) network from top 200 gene pairs of absolute association scores. Major biological processes from Gene Ontology are highlighted with their respective colors. [file DataSheet_2.zip › ANNE_Supplementary Figures S1-S7_Page_28.jpg]

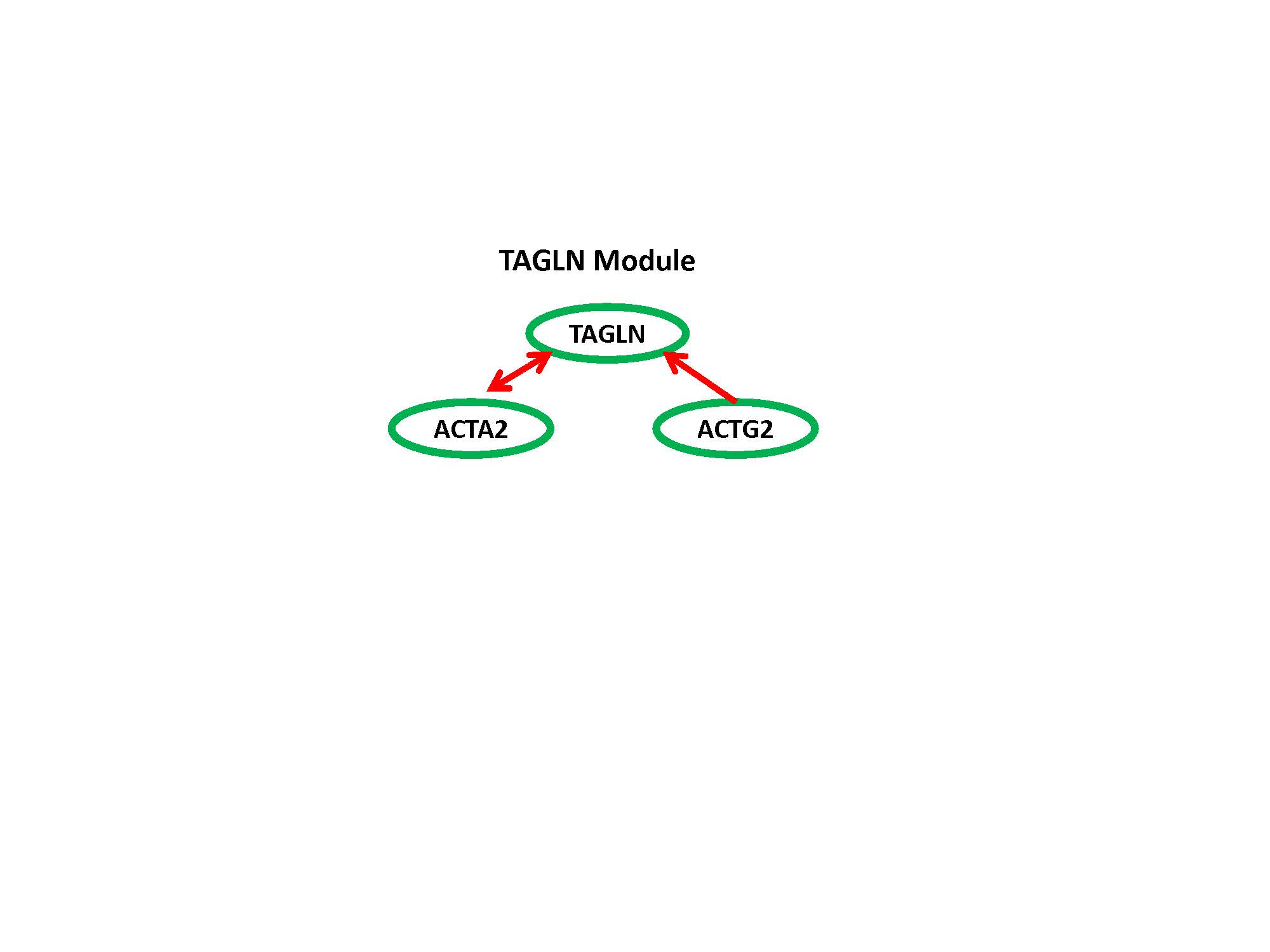

Supplement: Supplementary Figure 1 — Encoded chemosensitive (pCR) network from top 200 gene pairs of absolute association scores. Major biological processes from Gene Ontology are highlighted with their respective colors. [file DataSheet_2.zip › ANNE_Supplementary Figures S1-S7_Page_27.jpg]

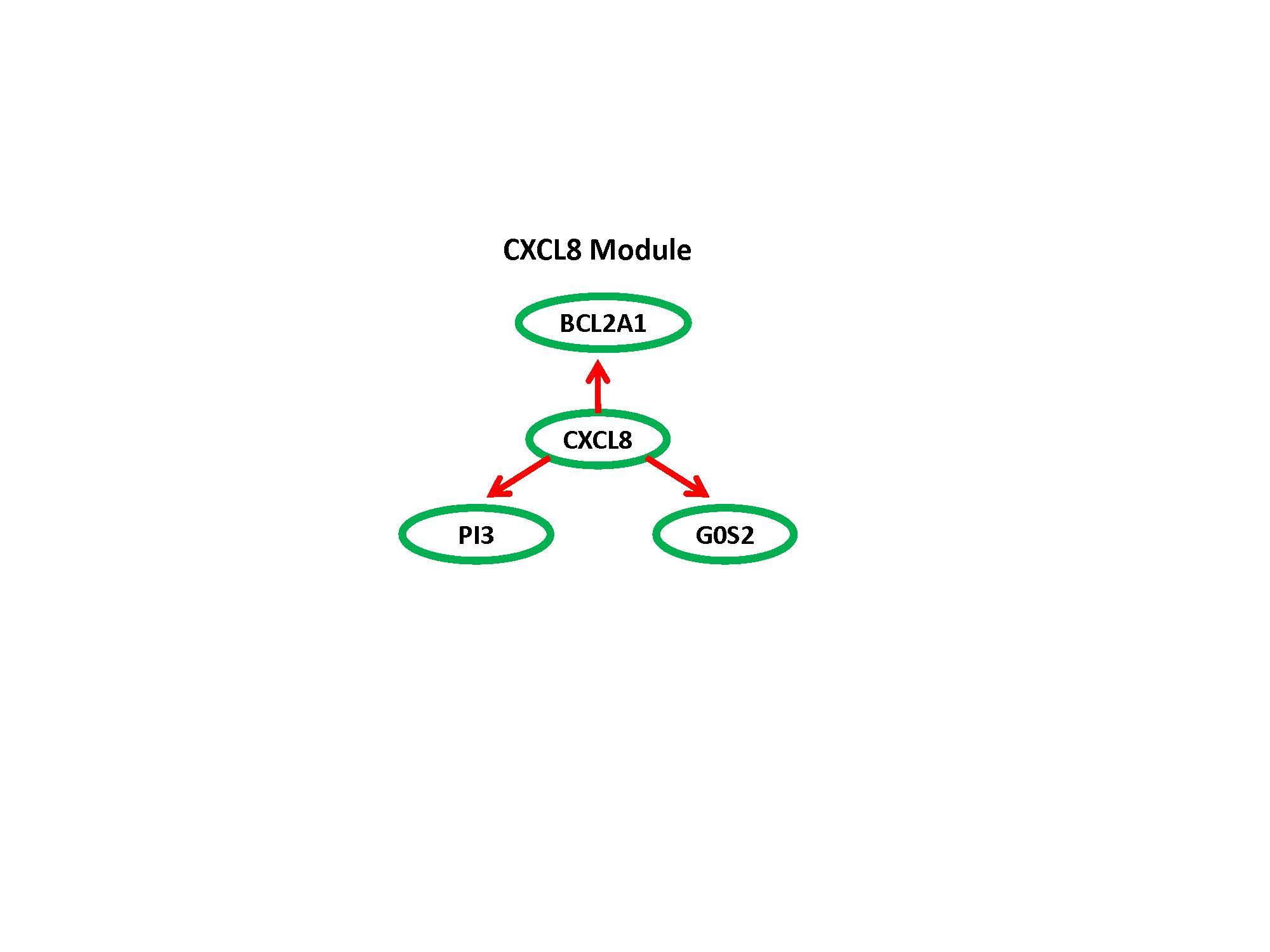

Supplement: Supplementary Figure 1 — Encoded chemosensitive (pCR) network from top 200 gene pairs of absolute association scores. Major biological processes from Gene Ontology are highlighted with their respective colors. [file DataSheet_2.zip › ANNE_Supplementary Figures S1-S7_Page_26.jpg]

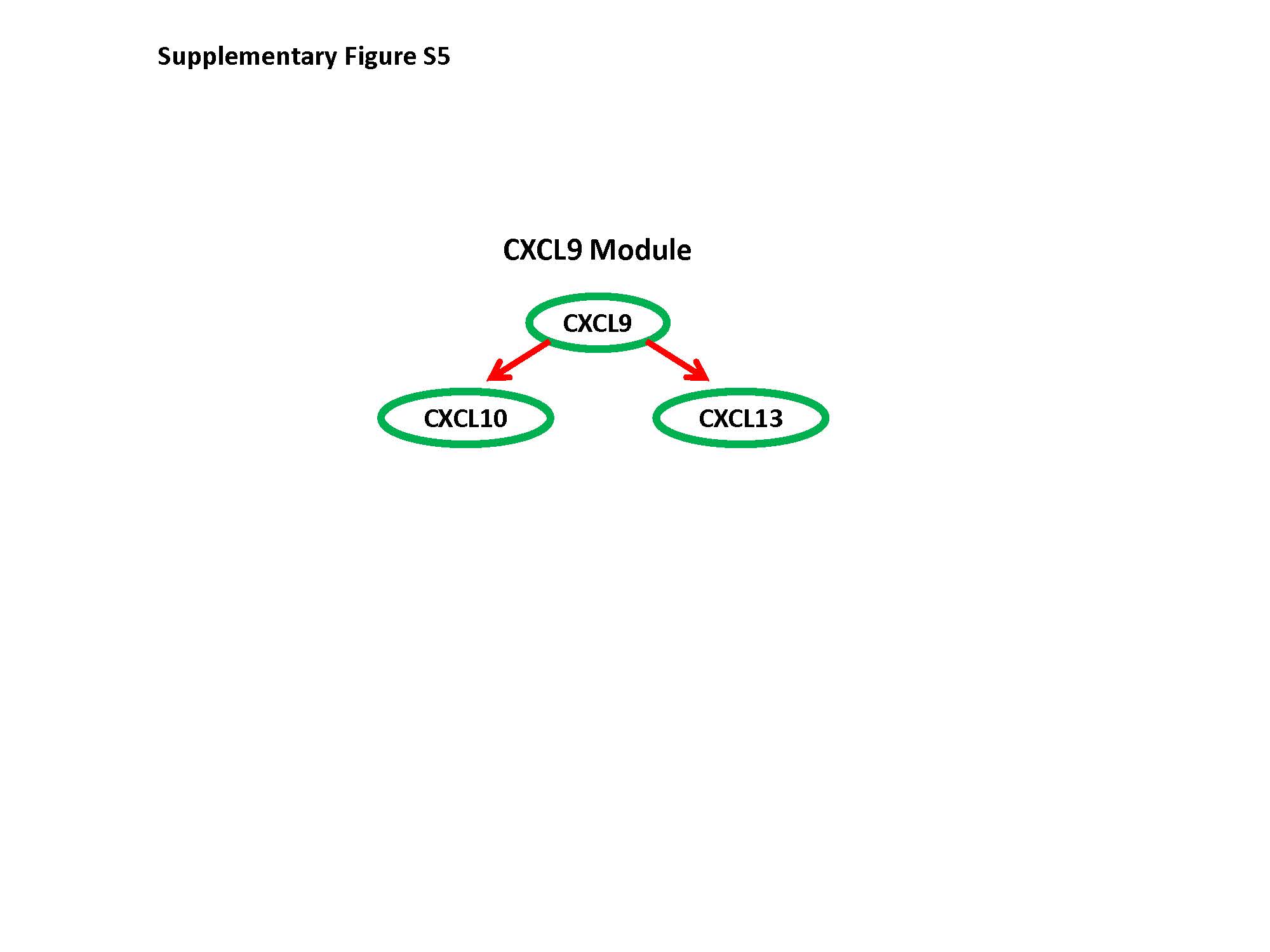

Supplement: Supplementary Figure 1 — Encoded chemosensitive (pCR) network from top 200 gene pairs of absolute association scores. Major biological processes from Gene Ontology are highlighted with their respective colors. [file DataSheet_2.zip › ANNE_Supplementary Figures S1-S7_Page_25.jpg]

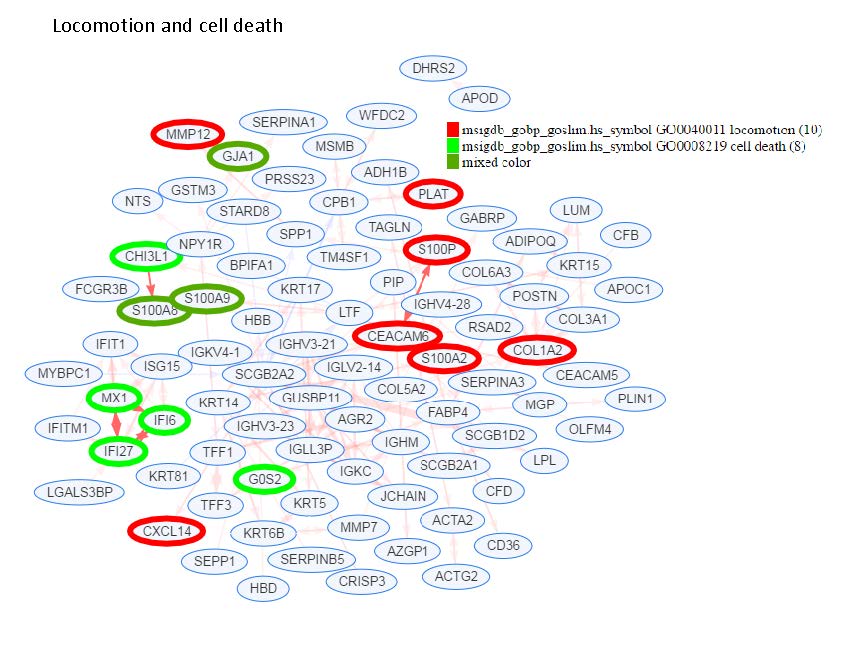

Supplement: Supplementary Figure 1 — Encoded chemosensitive (pCR) network from top 200 gene pairs of absolute association scores. Major biological processes from Gene Ontology are highlighted with their respective colors. [file DataSheet_2.zip › ANNE_Supplementary Figures S1-S7_Page_24.jpg]

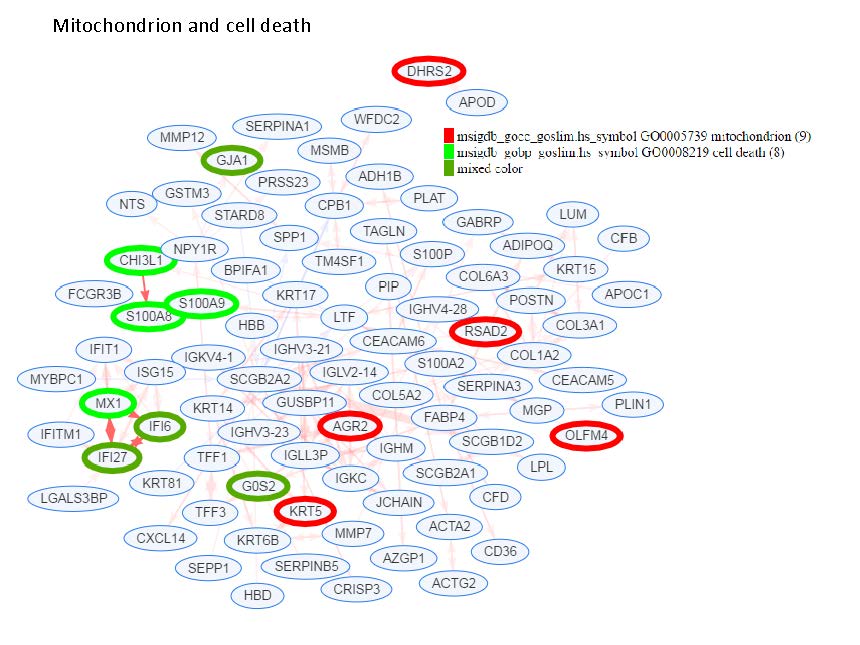

Supplement: Supplementary Figure 1 — Encoded chemosensitive (pCR) network from top 200 gene pairs of absolute association scores. Major biological processes from Gene Ontology are highlighted with their respective colors. [file DataSheet_2.zip › ANNE_Supplementary Figures S1-S7_Page_23.jpg]

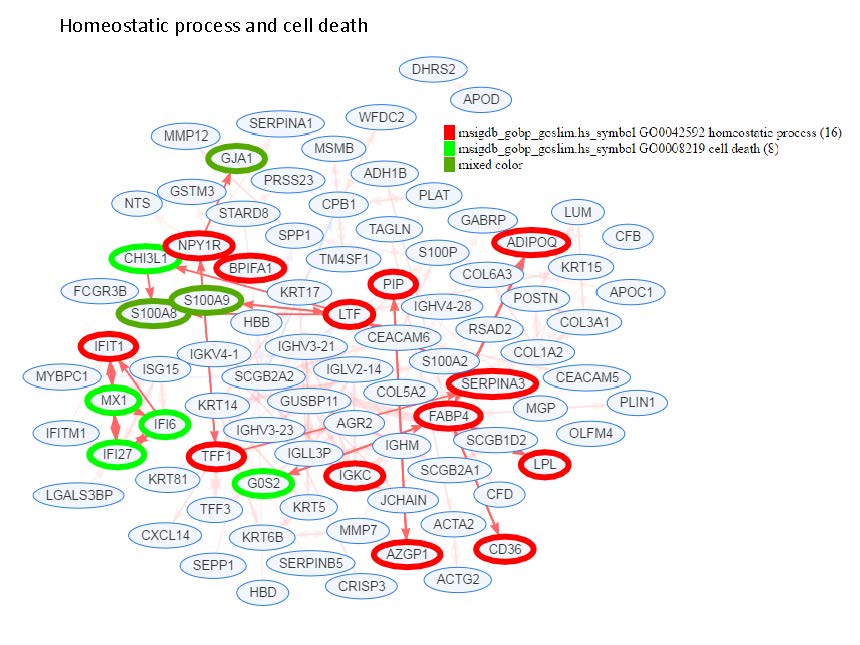

Supplement: Supplementary Figure 1 — Encoded chemosensitive (pCR) network from top 200 gene pairs of absolute association scores. Major biological processes from Gene Ontology are highlighted with their respective colors. [file DataSheet_2.zip › ANNE_Supplementary Figures S1-S7_Page_22.jpg]

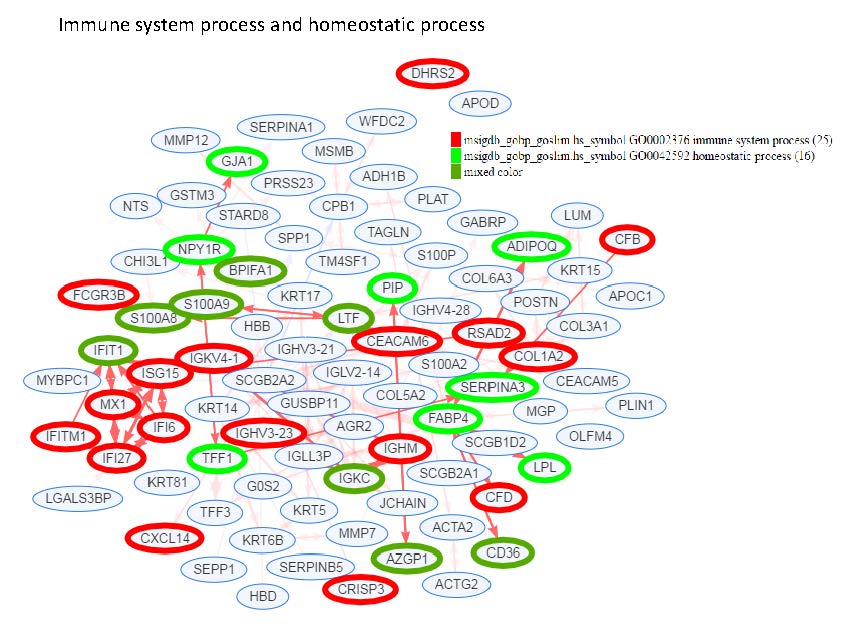

Supplement: Supplementary Figure 1 — Encoded chemosensitive (pCR) network from top 200 gene pairs of absolute association scores. Major biological processes from Gene Ontology are highlighted with their respective colors. [file DataSheet_2.zip › ANNE_Supplementary Figures S1-S7_Page_21.jpg]

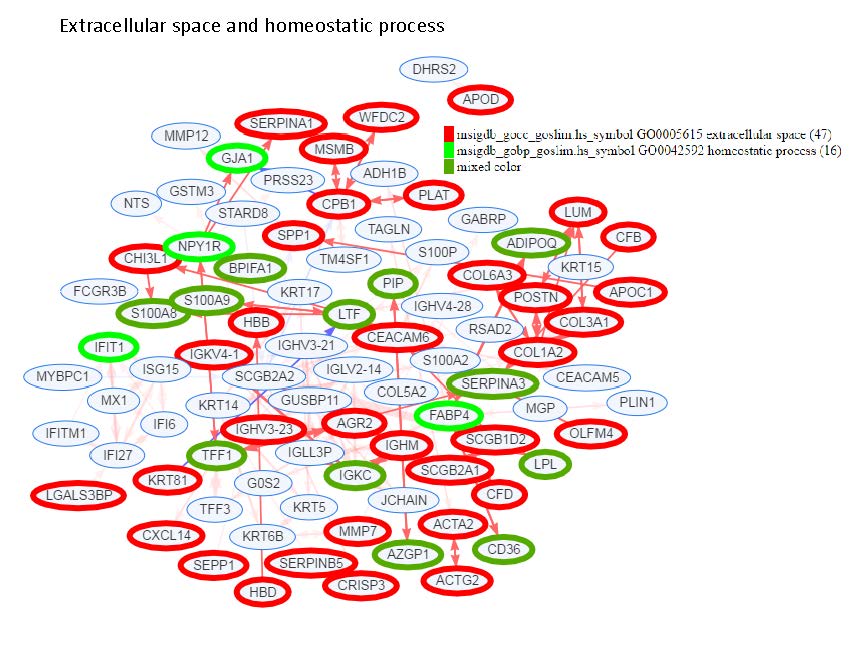

Supplement: Supplementary Figure 1 — Encoded chemosensitive (pCR) network from top 200 gene pairs of absolute association scores. Major biological processes from Gene Ontology are highlighted with their respective colors. [file DataSheet_2.zip › ANNE_Supplementary Figures S1-S7_Page_20.jpg]

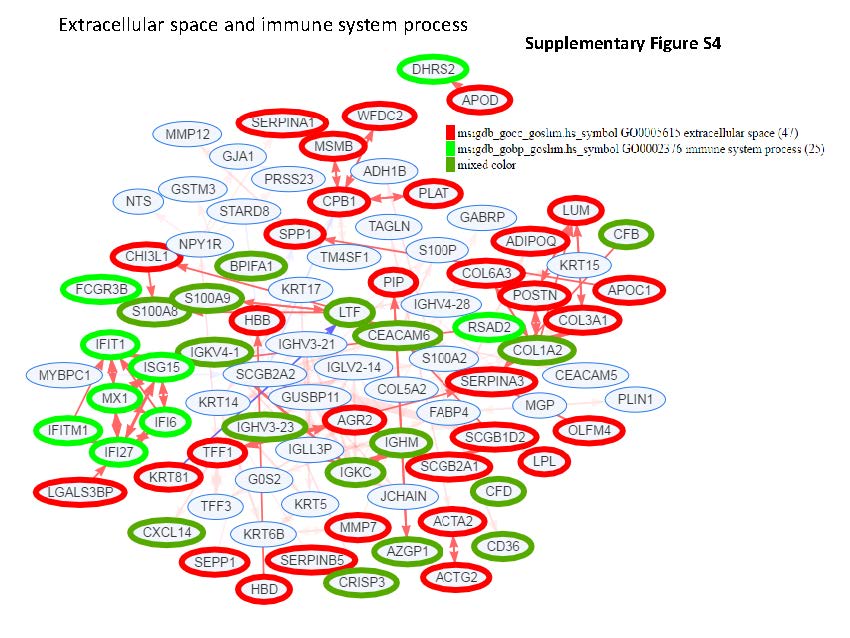

Supplement: Supplementary Figure 1 — Encoded chemosensitive (pCR) network from top 200 gene pairs of absolute association scores. Major biological processes from Gene Ontology are highlighted with their respective colors. [file DataSheet_2.zip › ANNE_Supplementary Figures S1-S7_Page_19.jpg]

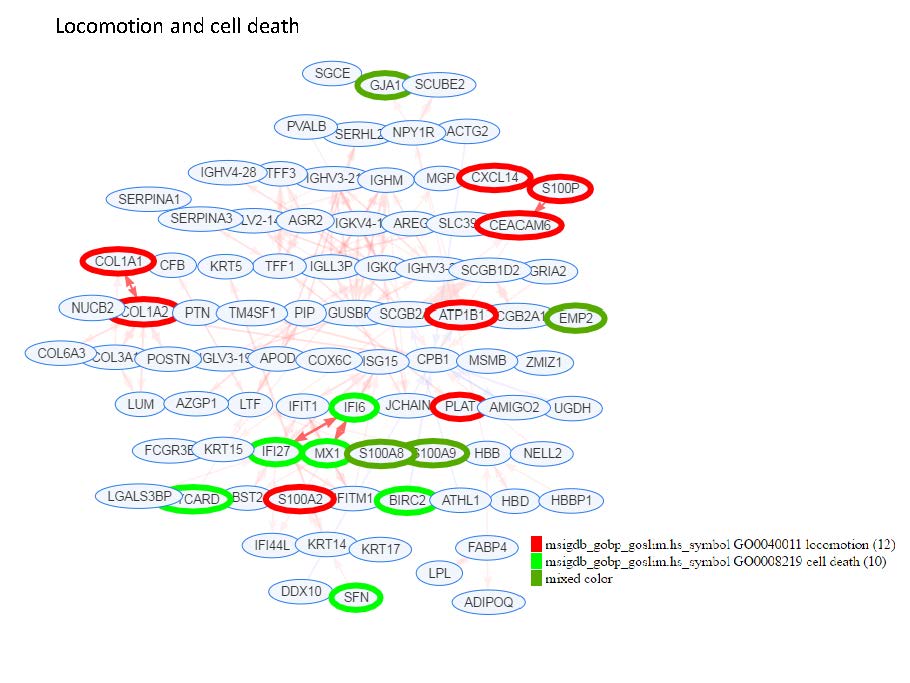

Supplement: Supplementary Figure 1 — Encoded chemosensitive (pCR) network from top 200 gene pairs of absolute association scores. Major biological processes from Gene Ontology are highlighted with their respective colors. [file DataSheet_2.zip › ANNE_Supplementary Figures S1-S7_Page_18.jpg]

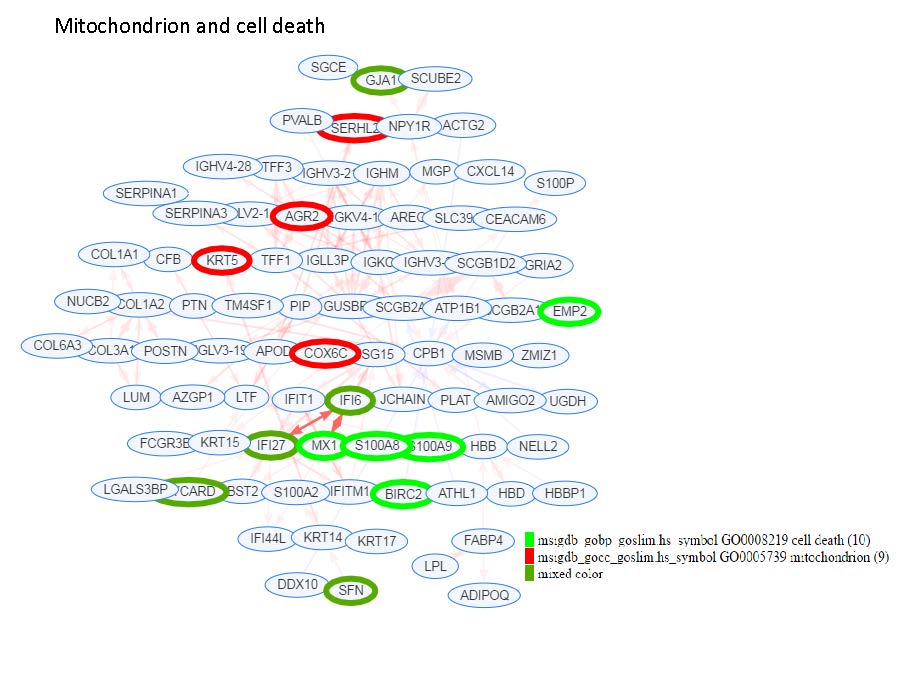

Supplement: Supplementary Figure 1 — Encoded chemosensitive (pCR) network from top 200 gene pairs of absolute association scores. Major biological processes from Gene Ontology are highlighted with their respective colors. [file DataSheet_2.zip › ANNE_Supplementary Figures S1-S7_Page_17.jpg]

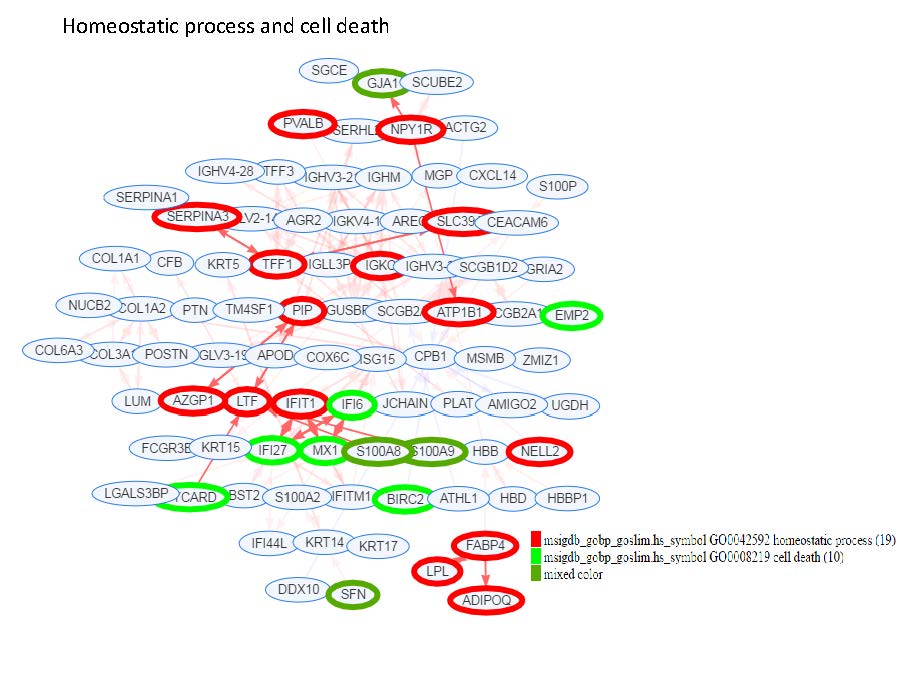

Supplement: Supplementary Figure 1 — Encoded chemosensitive (pCR) network from top 200 gene pairs of absolute association scores. Major biological processes from Gene Ontology are highlighted with their respective colors. [file DataSheet_2.zip › ANNE_Supplementary Figures S1-S7_Page_16.jpg]

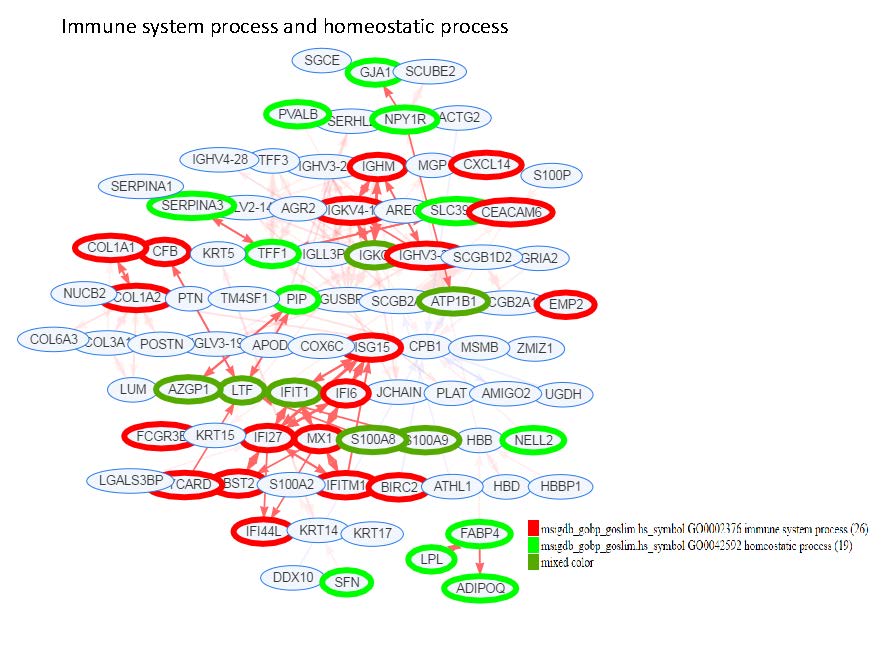

Supplement: Supplementary Figure 1 — Encoded chemosensitive (pCR) network from top 200 gene pairs of absolute association scores. Major biological processes from Gene Ontology are highlighted with their respective colors. [file DataSheet_2.zip › ANNE_Supplementary Figures S1-S7_Page_15.jpg]

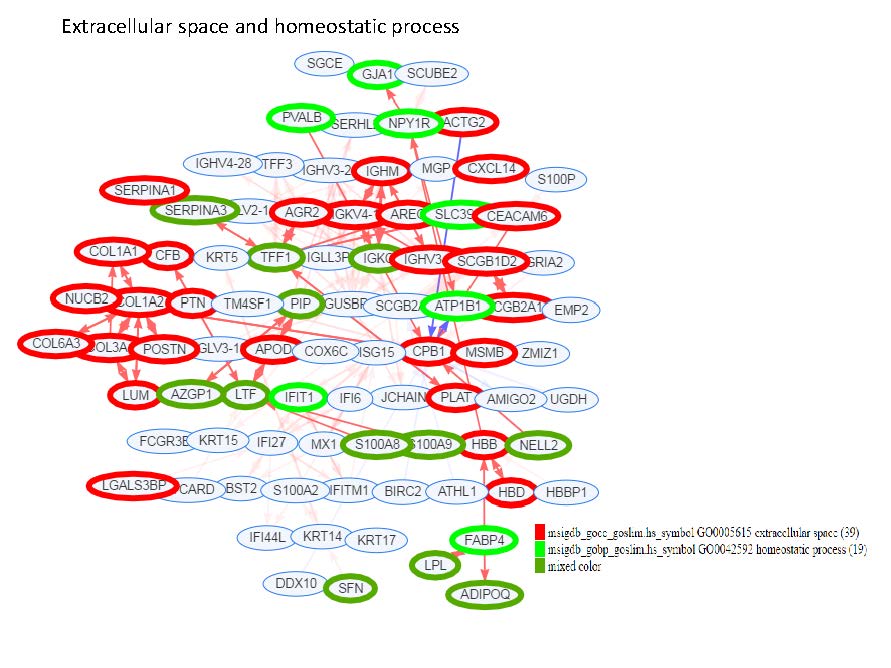

Supplement: Supplementary Figure 1 — Encoded chemosensitive (pCR) network from top 200 gene pairs of absolute association scores. Major biological processes from Gene Ontology are highlighted with their respective colors. [file DataSheet_2.zip › ANNE_Supplementary Figures S1-S7_Page_14.jpg]

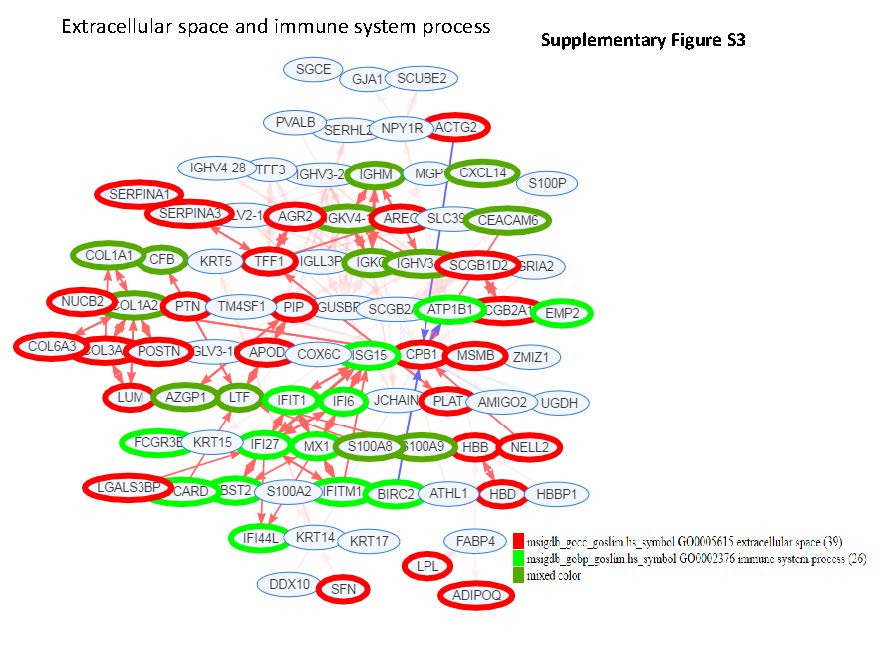

Supplement: Supplementary Figure 1 — Encoded chemosensitive (pCR) network from top 200 gene pairs of absolute association scores. Major biological processes from Gene Ontology are highlighted with their respective colors. [file DataSheet_2.zip › ANNE_Supplementary Figures S1-S7_Page_13.jpg]

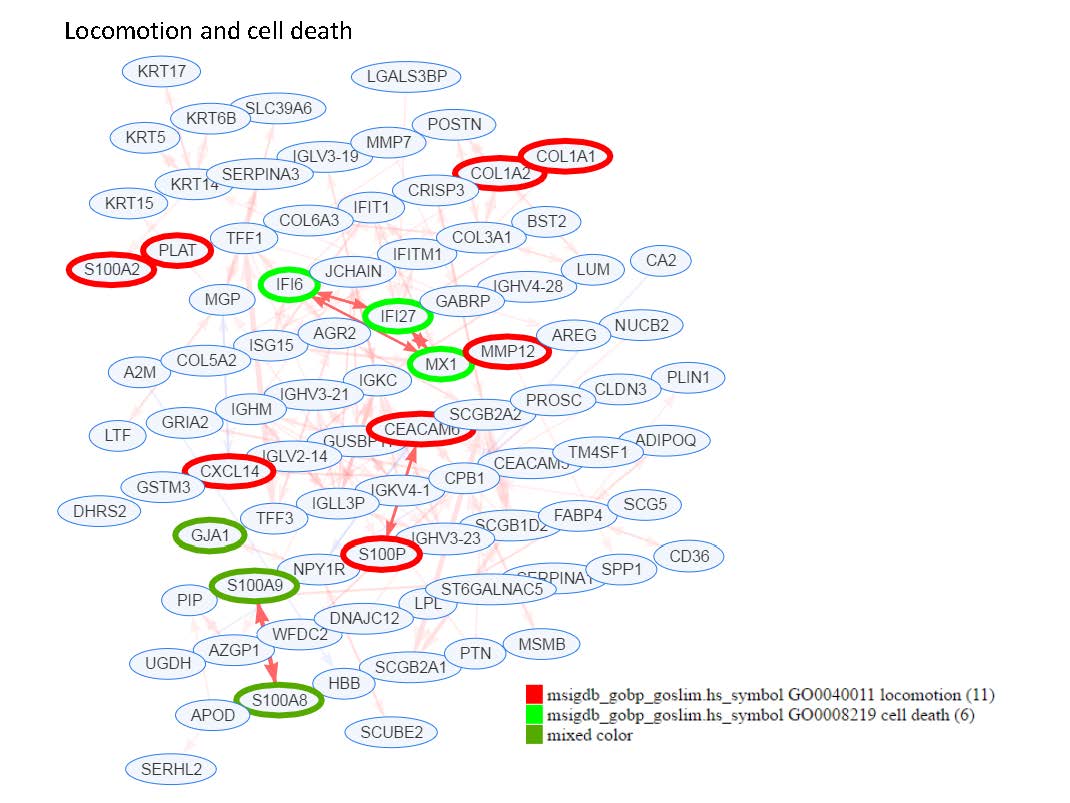

Supplement: Supplementary Figure 1 — Encoded chemosensitive (pCR) network from top 200 gene pairs of absolute association scores. Major biological processes from Gene Ontology are highlighted with their respective colors. [file DataSheet_2.zip › ANNE_Supplementary Figures S1-S7_Page_12.jpg]

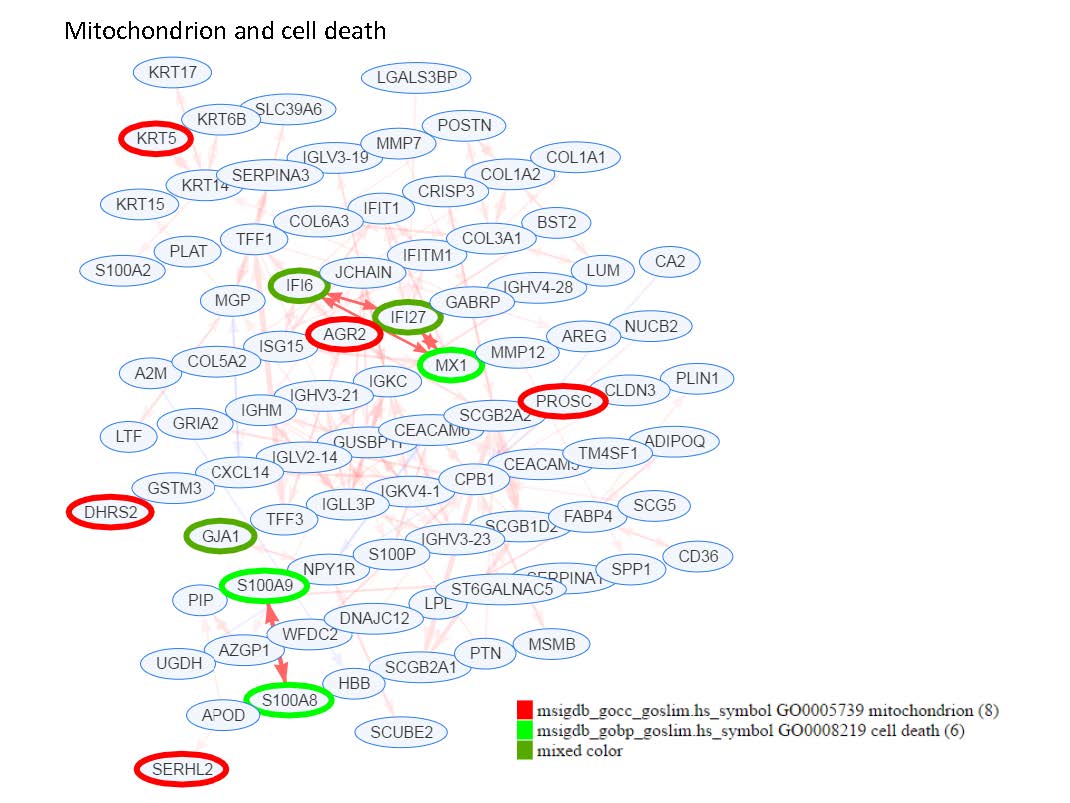

Supplement: Supplementary Figure 1 — Encoded chemosensitive (pCR) network from top 200 gene pairs of absolute association scores. Major biological processes from Gene Ontology are highlighted with their respective colors. [file DataSheet_2.zip › ANNE_Supplementary Figures S1-S7_Page_11.jpg]

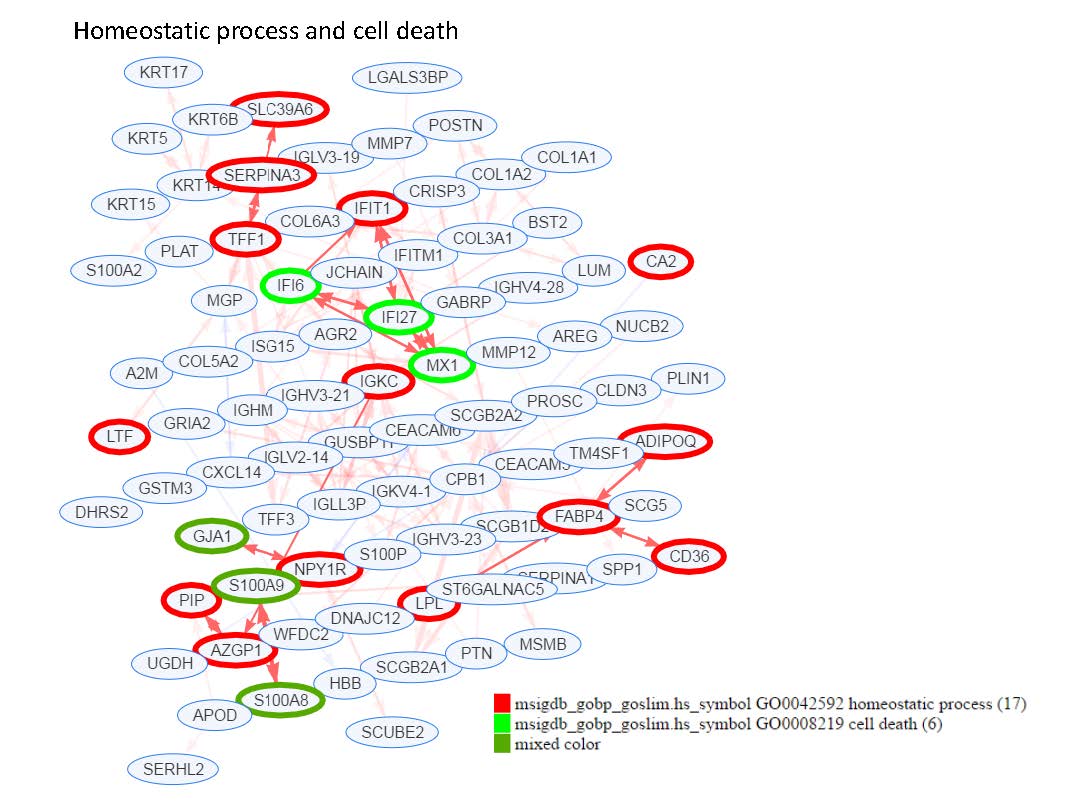

Supplement: Supplementary Figure 1 — Encoded chemosensitive (pCR) network from top 200 gene pairs of absolute association scores. Major biological processes from Gene Ontology are highlighted with their respective colors. [file DataSheet_2.zip › ANNE_Supplementary Figures S1-S7_Page_10.jpg]

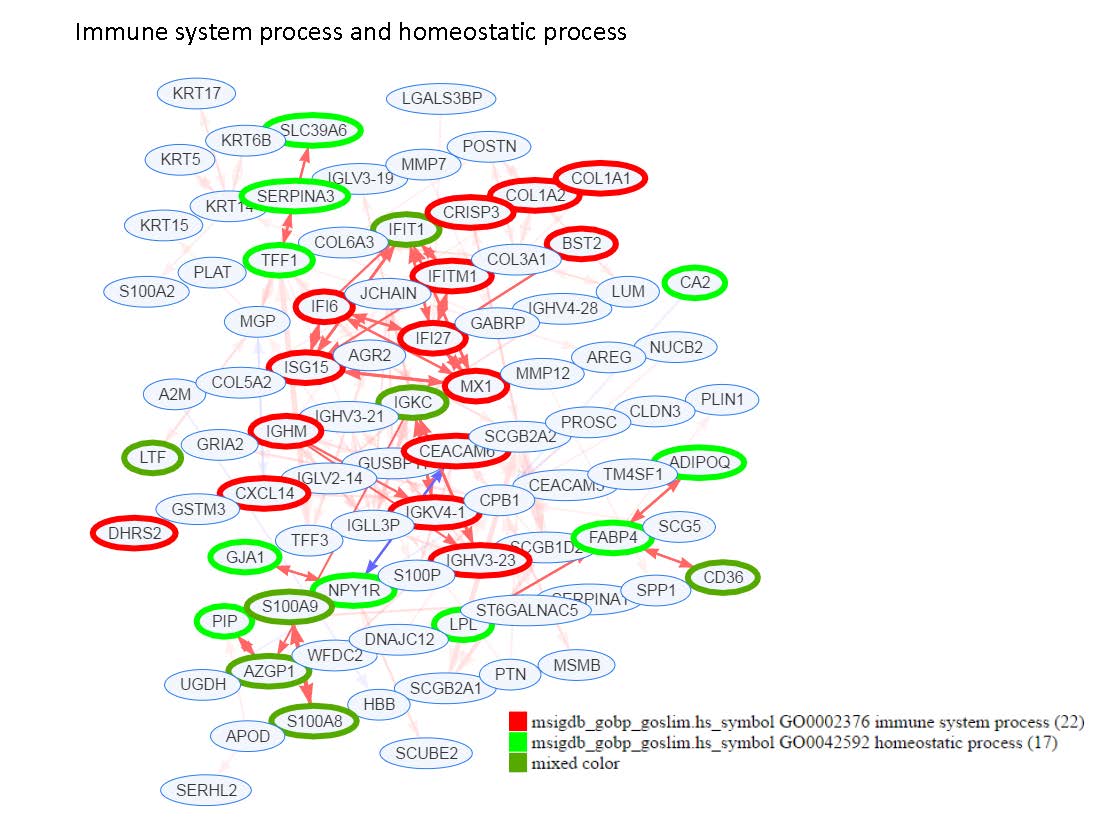

Supplement: Supplementary Figure 1 — Encoded chemosensitive (pCR) network from top 200 gene pairs of absolute association scores. Major biological processes from Gene Ontology are highlighted with their respective colors. [file DataSheet_2.zip › ANNE_Supplementary Figures S1-S7_Page_09.jpg]

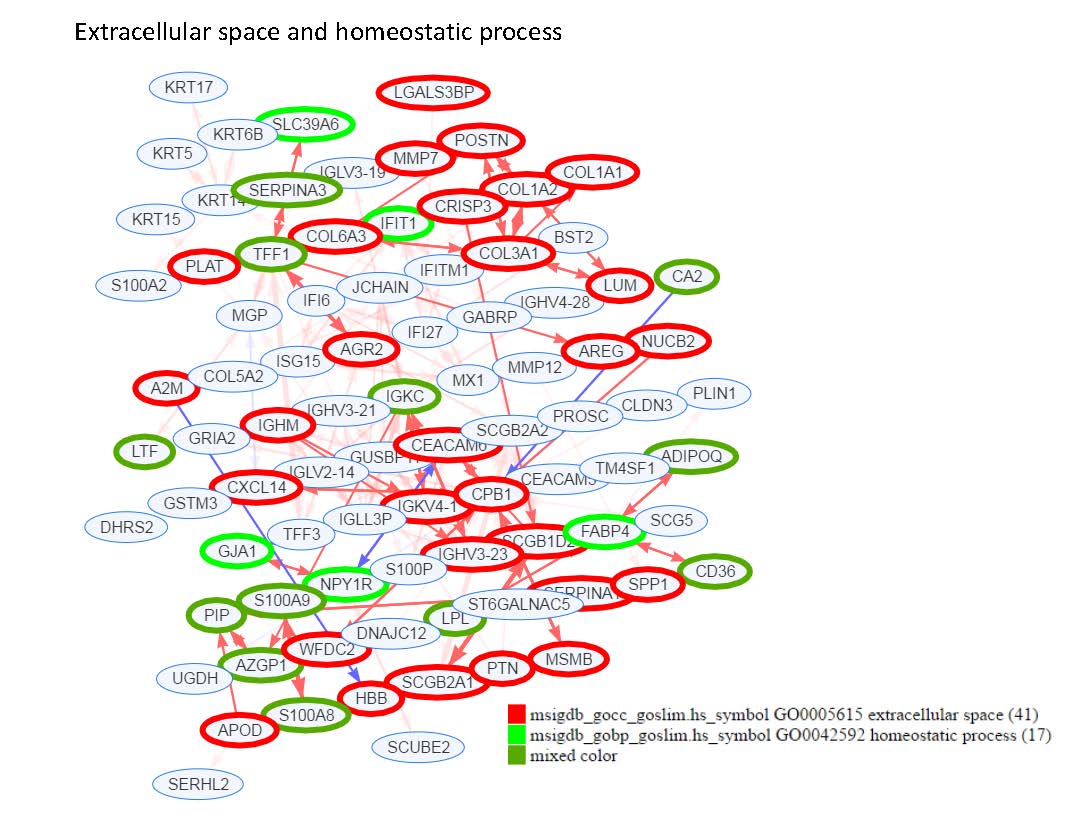

Supplement: Supplementary Figure 1 — Encoded chemosensitive (pCR) network from top 200 gene pairs of absolute association scores. Major biological processes from Gene Ontology are highlighted with their respective colors. [file DataSheet_2.zip › ANNE_Supplementary Figures S1-S7_Page_08.jpg]

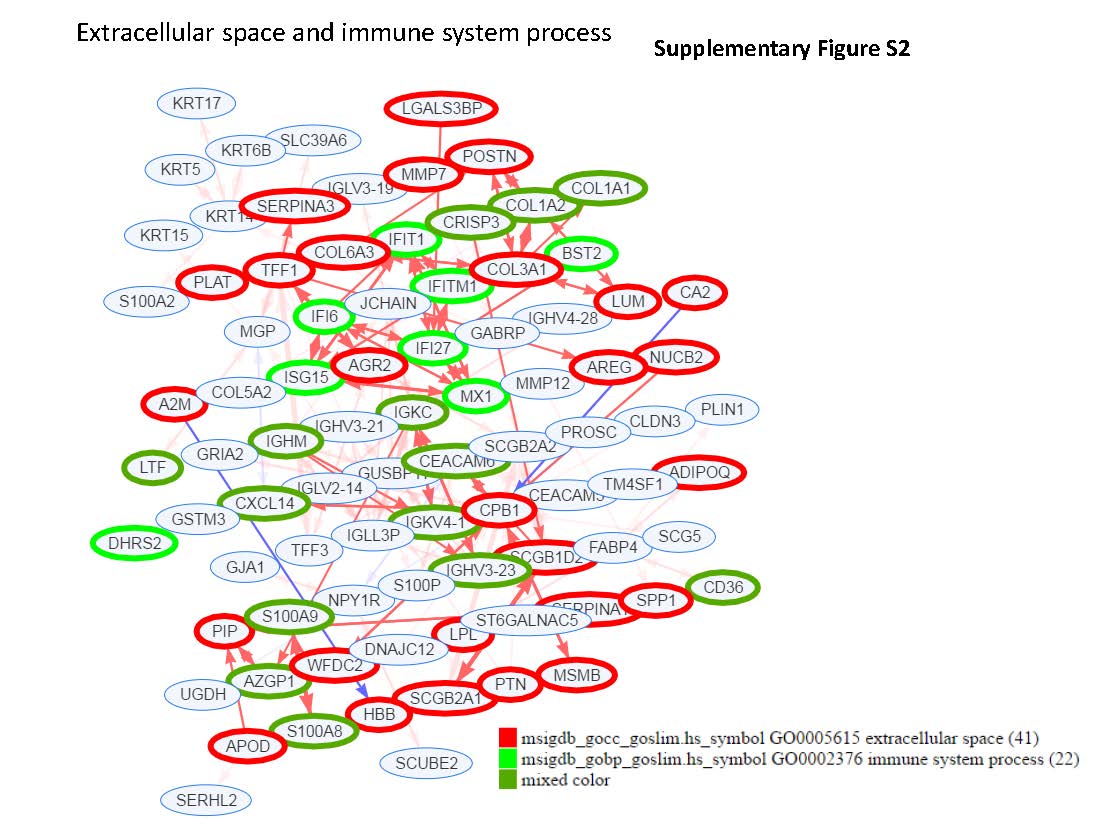

Supplement: Supplementary Figure 1 — Encoded chemosensitive (pCR) network from top 200 gene pairs of absolute association scores. Major biological processes from Gene Ontology are highlighted with their respective colors. [file DataSheet_2.zip › ANNE_Supplementary Figures S1-S7_Page_07.jpg]

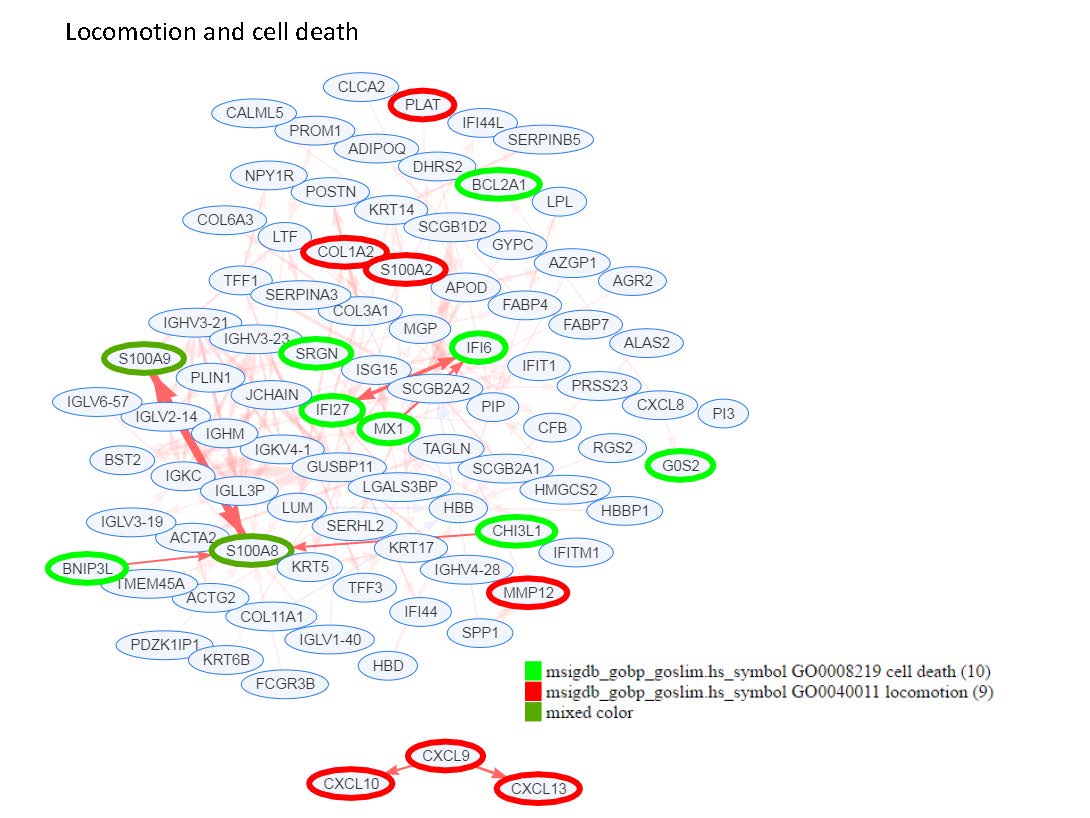

Supplement: Supplementary Figure 1 — Encoded chemosensitive (pCR) network from top 200 gene pairs of absolute association scores. Major biological processes from Gene Ontology are highlighted with their respective colors. [file DataSheet_2.zip › ANNE_Supplementary Figures S1-S7_Page_06.jpg]

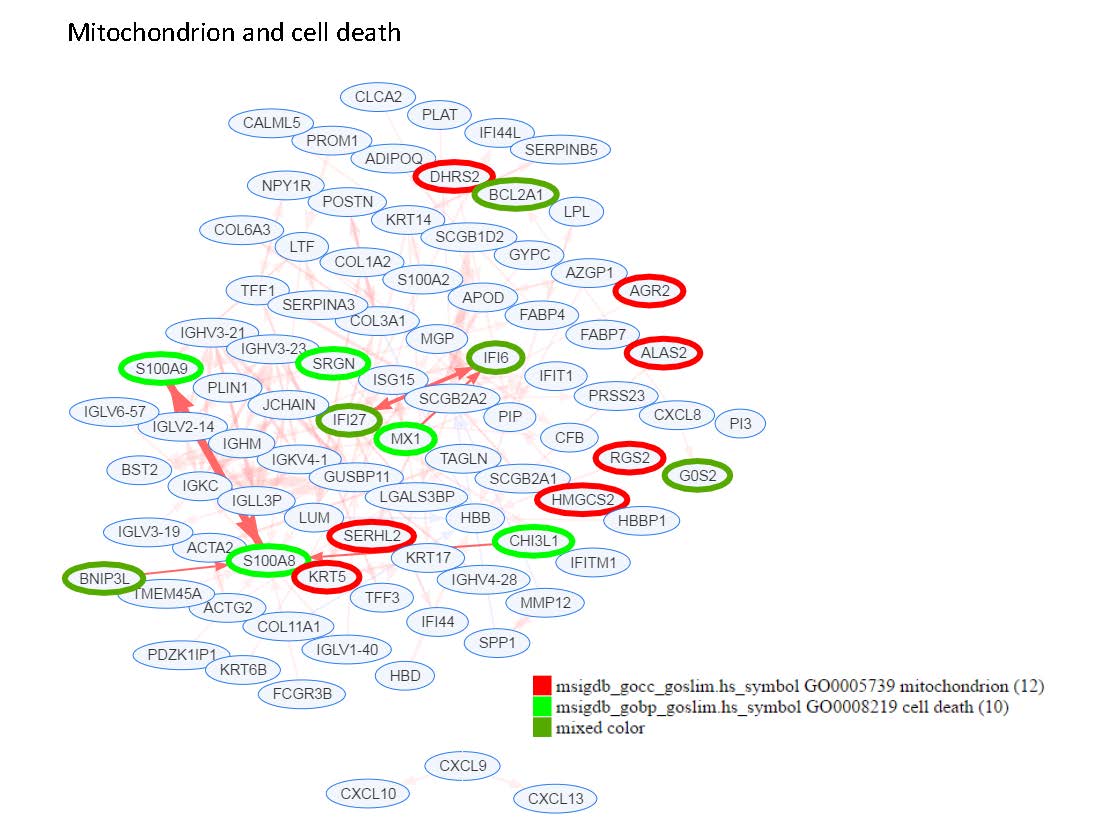

Supplement: Supplementary Figure 1 — Encoded chemosensitive (pCR) network from top 200 gene pairs of absolute association scores. Major biological processes from Gene Ontology are highlighted with their respective colors. [file DataSheet_2.zip › ANNE_Supplementary Figures S1-S7_Page_05.jpg]

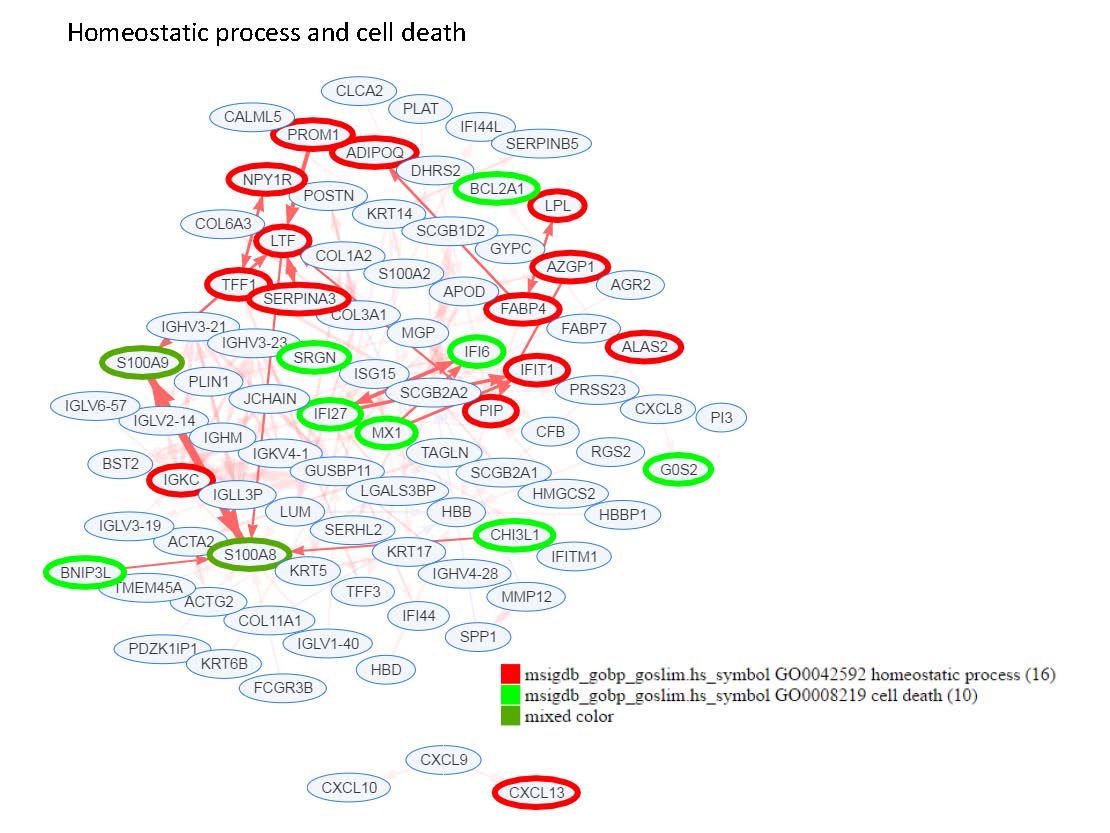

Supplement: Supplementary Figure 1 — Encoded chemosensitive (pCR) network from top 200 gene pairs of absolute association scores. Major biological processes from Gene Ontology are highlighted with their respective colors. [file DataSheet_2.zip › ANNE_Supplementary Figures S1-S7_Page_04.jpg]

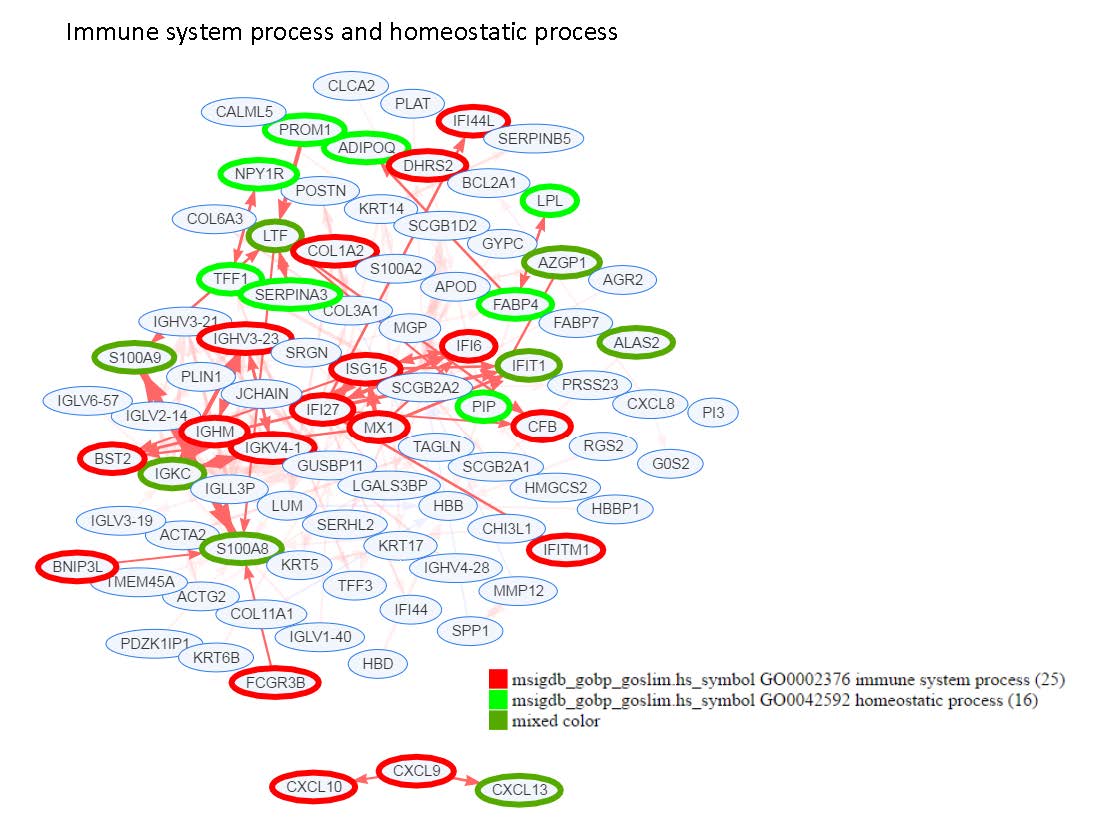

Supplement: Supplementary Figure 1 — Encoded chemosensitive (pCR) network from top 200 gene pairs of absolute association scores. Major biological processes from Gene Ontology are highlighted with their respective colors. [file DataSheet_2.zip › ANNE_Supplementary Figures S1-S7_Page_03.jpg]

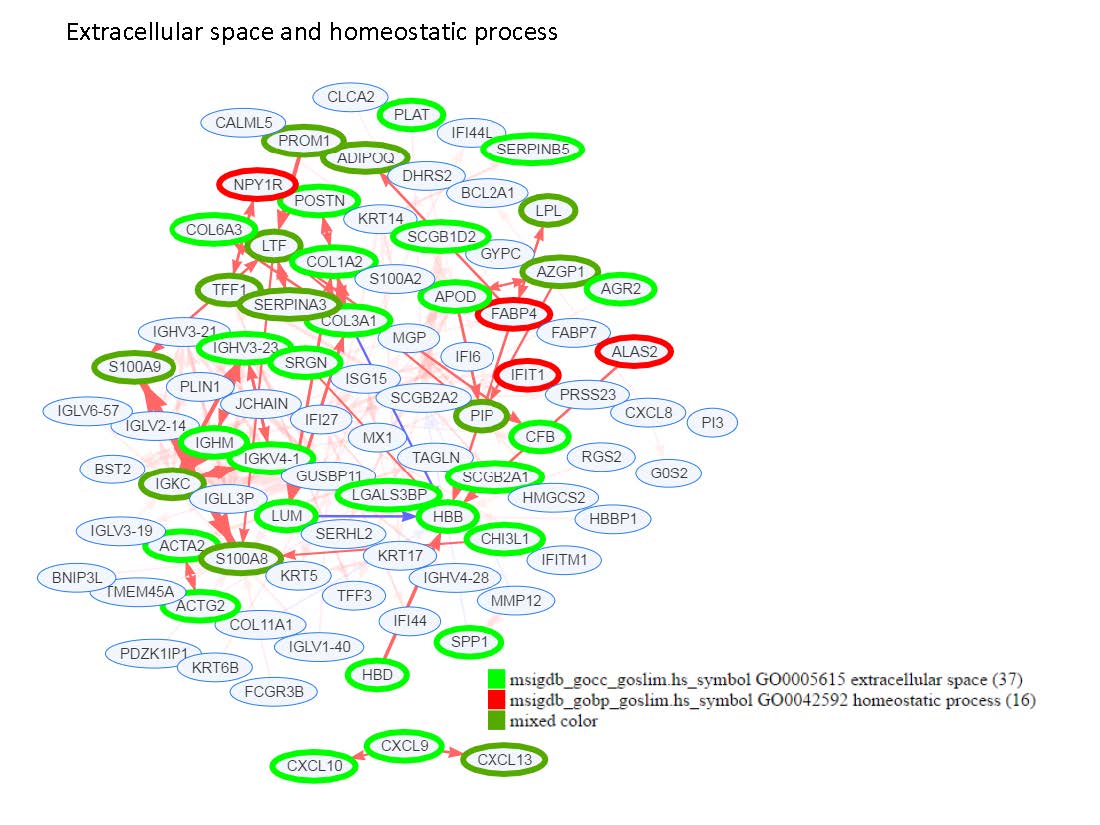

Supplement: Supplementary Figure 1 — Encoded chemosensitive (pCR) network from top 200 gene pairs of absolute association scores. Major biological processes from Gene Ontology are highlighted with their respective colors. [file DataSheet_2.zip › ANNE_Supplementary Figures S1-S7_Page_02.jpg]
